# Ruthenafuran Complexes Supported by the Bipyridine-Bis(diphenylphosphino)methane Ligand Set: Synthesis and Cytotoxicity Studies

**DOI:** 10.3390/molecules27051709

**Published:** 2022-03-05

**Authors:** Chi-Fung Yeung, Sik-Him Tang, Zhe Yang, Tsun-Yin Li, Ka-Kit Li, Yuen-Man Chan, Hau-Lam Shek, Kai-Wa Io, King-Ting Tam, Shek-Man Yiu, Man-Kit Tse, Chun-Yuen Wong

**Affiliations:** 1 Department of Chemistry, City University of Hong Kong, Tat Chee Avenue, Kowloon, Hong Kong; chifyeung3-c@my.cityu.edu.hk (C.-F.Y.); sikhtang3-c@my.cityu.edu.hk (S.-H.T.); zyang36-c@my.cityu.edu.hk (Z.Y.); tsunyinli7-c@my.cityu.edu.hk (T.-Y.L.); kakitli8-c@my.cityu.edu.hk (K.-K.L.); symchan@cityu.edu.hk (Y.-M.C.); hlshek5-c@my.cityu.edu.hk (H.-L.S.); kaiio2-c@my.cityu.edu.hk (K.-W.I.); kingttam3-c@my.cityu.edu.hk (K.-T.T.); kensmyiu@cityu.edu.hk (S.-M.Y.); manktse@cityu.edu.hk (M.-K.T.); 2 State Key Laboratory of Terahertz and Millimeter Waves, City University of Hong Kong, Tat Chee Avenue, Kowloon, Hong Kong

**Keywords:** ruthenium, alkyne activation, metallafuran, cytotoxicity

## Abstract

Mononuclear and dinuclear Ru(II) complexes *cis*-[Ru(κ^2^-dppm)(bpy)Cl_2_] (**1**), *cis*-[Ru(κ^2^-dppe)(bpy)Cl_2_] (**2**) and [Ru_2_(bpy)_2_(μ-dpam)_2_(μ-Cl)_2_](Cl)_2_ ([**3**](Cl)_2_) were prepared from the reactions between *cis*(Cl), *cis*(S)-[Ru(bpy)(dmso-*S*)_2_Cl_2_] and diphosphine/diarsine ligands (bpy = 2,2′-bipyridine; dppm = 1,1-bis(diphenylphosphino)methane; dppe = 1,2-bis(diphenylphosphino)ethane; dpam = 1,1-bis(diphenylarsino)methane). While methoxy-substituted ruthenafuran [Ru(bpy)(κ^2^-dppe)(C^O)]^+^ ([**7**]^+^; C^O = anionic bidentate [*C*(OMe)CHC(Ph)*O*]^−^ chelate) was obtained as the only product in the reaction between **2** and phenyl ynone HC≡C(C=O)Ph in MeOH, replacing **2** with **1** led to the formation of both methoxy-substituted ruthenafuran [Ru(bpy)(κ^2^-dppm)(C^O)]^+^ ([**4**]^+^) and phosphonium-ring-fused bicyclic ruthenafuran [Ru(bpy)(P^C^O)Cl]^+^ ([**5**]^+^; P^C^O = neutral tridentate [(Ph)_2_*P*CH_2_P(Ph)_2_*C*CHC(Ph)*O*] chelate). All of these aforementioned metallafuran complexes were derived from Ru(II)–vinylidene intermediates. The potential applications of these metallafuran complexes as anticancer agents were evaluated by in vitro cytotoxicity studies against cervical carcinoma (HeLa) cancer cell line. All the ruthenafuran complexes were found to be one order of magnitude more cytotoxic than cisplatin, which is one of the metal-based anticancer agents being widely used currently.

## 1. Introduction

**Scheme 1:**
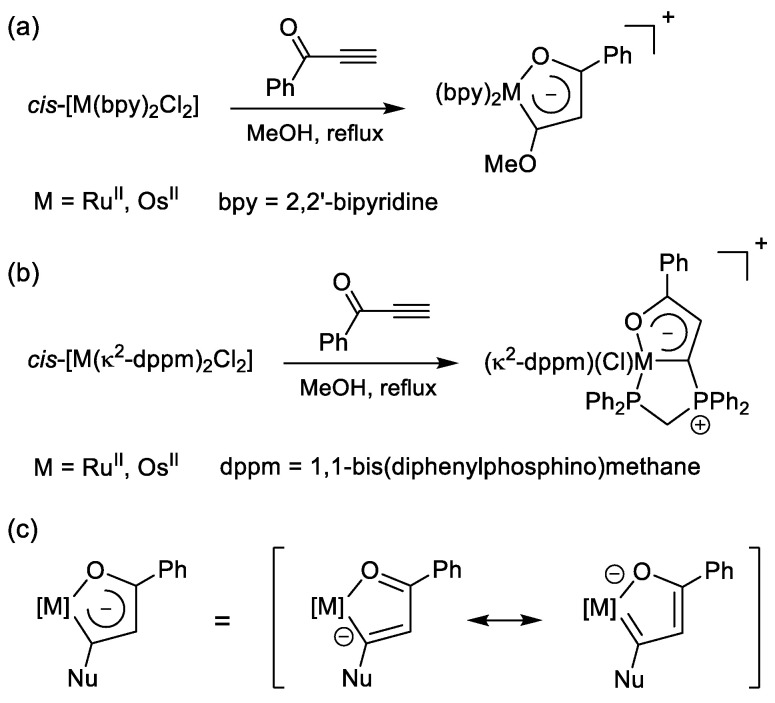
Formation of different metallafuran complexes from the reactions between phenyl ynone and (**a**) *cis*-[Ru/Os(bpy)_2_Cl_2_], (**b**) *cis*-[Ru/Os(κ^2^-dppm)_2_Cl_2_]. (**c**) Mesomeric structures for the metallafuran moieties depicted in (**a**,**b**).

Activation of alkynes by transition-metal complexes has recently gained increasing research interest in the field of organometallic chemistry [1,2,3,4,5,6,7,8,9,10,11,12,13,14,15,16,17,18,19,20,21,22,23,24,25,26,27,28,29,30]. Although the formation of metal–vinylidene species via alkyne–vinylidene rearrangement has been regarded as a key step in Ru(II)-induced alkyne transformations, a number of our synthetic studies revealed that Ru(II) can also activate alkynes via “non-vinylidene” pathways [31,32]. With the aim to gain control on the modes of alkyne activation, we initiated research activities on probing and isolating intermediates and products from the reactions between functionalized alkynes and a variety of Fe(II), Ru(II) and Os(II) complexes [31,32,33,34,35,36,37,38,39,40,41,42,43,44,45,46,47]. In 2015, we reported the synthesis of metallafuran complexes in the form of [M(bpy)_2_(C^O)]^+^ from the reactions between *cis*-[M(bpy)_2_Cl_2_] (M = Ru, Os; bpy = 2,2′-bipyridine) and ynone HC≡C(C=O)Ph in MeOH (Scheme 1a; C^O represents an anionic bidentate [*C*(OMe)CHC(Ph)*O*]^−^ chelate, coordinating atoms in italics) [38]. Later in 2019, we discovered that reactions between *cis*-[M(κ^2^-dppm)_2_Cl_2_] (M = Ru, Os; dppm = 1,1-bis(diphenylphosphino)methane) and HC≡C(C=O)Ph in MeOH gave the phosphonium-ring-fused bicyclic metallafuran complexes in the form of [M(κ^2^-dppm)(P^C^O)Cl]^+^, but not [M(κ^2^-dppm)_2_(C^O)]^+^ (Scheme 1b; P^C^O represents a neutral tridentate [(Ph)_2_*P*CH_2_P(Ph)_2_*C*CHC(Ph)*O*] chelate) [44]. This striking difference in reactivity suggested the participation of dppm in the reaction and led us to examine the reactions between HC≡C(C=O)Ph and [Ru(κ^2^-L^L)(bpy)Cl_2_], where L^L represents bidentate phosphine or arsine ligands. We, in this paper, report (1) our attempts to synthesize various Ru(II) precursors in the form of [Ru(κ^2^-L^L)(bpy)Cl_2_], (2) their reactivity towards ynone HC≡C(C=O)Ph, and (3) the cytotoxicity of the resultant complexes.

## 2. Results and Discussion

### 2.1. Synthesis of Metal Precursors

**Scheme 2:**
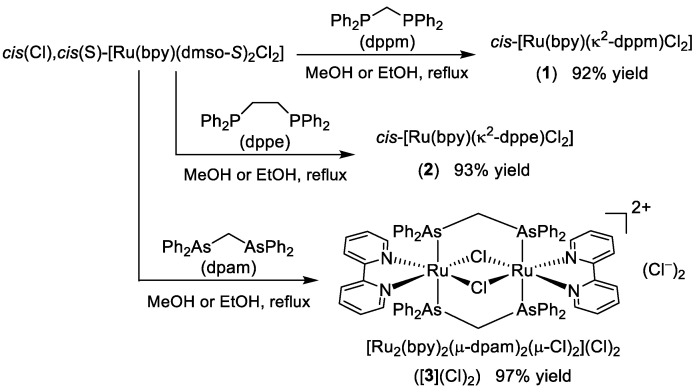
Reactions between *cis*(Cl), *cis*(S)-[Ru(bpy)(dmso-*S*)_2_Cl_2_] and bidentate phosphine/arsine ligands.

**Figure 1:**
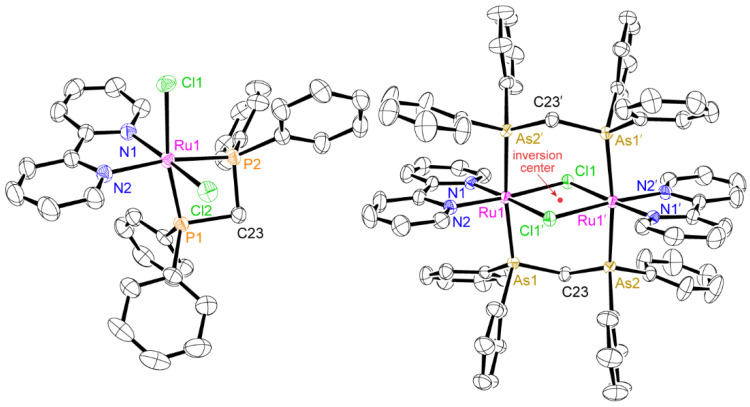
Perspective views of **1** (left) and [**3**]^2+^ (right) as represented by 50% probability ellipsoids (hydrogen atoms are omitted for clarity). Selected bond lengths (Å) and angles (°): **1**: Ru1–Cl1 2.5059(6), Ru1–Cl2 2.4387(7), Ru1–N1 2.063(2), Ru1–N2 2.115(2), Ru1–P1 2.2654(7), Ru1–P2 2.2966(7), P1–Ru1–P 2 72.11(2), P1–C23–P2 93.25(11); [**3**]^2+^: Ru1–Cl1 2.4418(8), Ru1–Cl1′ 2.4383(8), Ru1–N1 2.031(3), Ru1–N2 2.046(3), Ru1–As1 2.4491(4), Ru1–As2 2.4570(4), Cl1–Ru1–Cl1′ 83.00(3), Ru1–Cl1–Ru1′ 97.00(3), As1–C23–As2 118.16(17).

Attempts were made to prepare Ru(II) precursors in the form of [Ru(κ^2^-L^L)(bpy)Cl_2_] by reacting *cis*(Cl),*cis*(S)-[Ru(bpy)(dmso-*S*)_2_Cl_2_] with one equivalent of dppm, dppe (1,2-bis(diphenylphosphino)ethane) or dpam (1,1-bis(diphenylarsino)methane) in alcoholic solvents (Scheme 2). While mononuclear Ru(II) precursors *cis*-[Ru(κ^2^-dppm)(bpy)Cl_2_] (**1**) and *cis*-[Ru(κ^2^-dppe)(bpy)Cl_2_] (**2**) were obtained as expected, the reaction involving dpam did not lead to any analogous mononuclear precursor but a dinuclear Ru(II) complex [Ru_2_(bpy)_2_(μ-dpam)_2_(μ-Cl)_2_](Cl)_2_ ([**3**](Cl)_2_). The molecular structures for **1**⋅CH_2_Cl_2_ and [**3**](OTf)_2_⋅2CH_2_Cl_2_ were determined by X-ray crystallography (Figure 1). While the solid-state structure of [**3**](OTf)_2_ revealed the existence of an inversion center at the center of the [Ru_2_Cl_2_] rhomboid in [**3**]^2+^, both the ^1^H and ^13^C spectra signified that [**3**]^2+^ possessed a pseudo D_2h_ symmetry in solution on the NMR time scale at room temperature (see Supplementary Materials). 

### 2.2. Reactions between the Metal Precursors and Phenyl Ynone

**Scheme 3:**
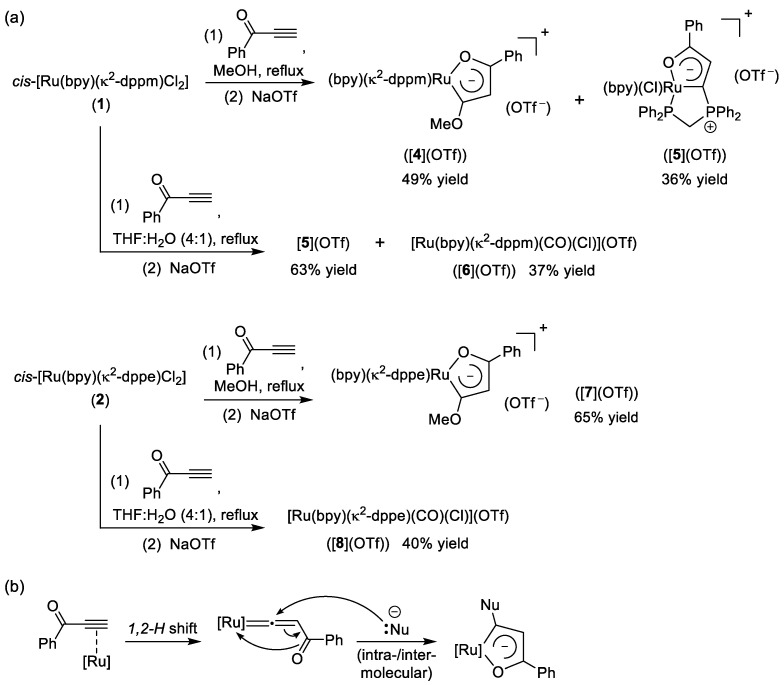
(**a**) Reactions between phenyl ynone HC≡C(C=O)Ph and metal precursors **1** and **2**, and (**b**) a plausible mechanism for the formation of metallafuran complexes in this work.

Reactions between phenyl ynone HC≡C(C=O)Ph and metal precursors **1**, **2**, and [**3**](Cl)_2_ were investigated. While the ynone did not react with [**3**](Cl)_2_, the reactions between HC≡C(C=O)Ph and metal precursors **1** and **2** led to different products, depending on the reaction conditions (Scheme 3a). A mixture of methoxy-substituted ruthenafuran [Ru(bpy)(κ^2^-dppm)(C^O)]^+^ ([**4**]^+^; C^O = anionic bidentate [*C*(OMe)CHC(Ph)*O*]^−^ chelate) and phosphonium-ring-fused bicyclic ruthenafuran [Ru(bpy)(P^C^O)Cl]^+^ ([**5**]^+^; P^C^O = neutral tridentate [(Ph)_2_*P*CH_2_P(Ph)_2_*C*CHC(Ph)*O*] chelate) was obtained when the reaction between **1** and HC≡C(C=O)Ph was performed in MeOH. Separation of this mixture could be conveniently performed by column chromatography. 

**Figure 2:**
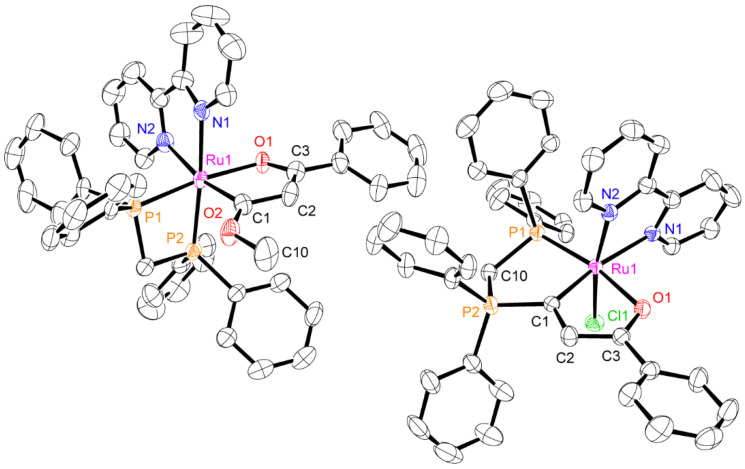
Perspective views of [**4**]^+^ (left) and [**5**]^+^ (right) as represented by 50% probability ellipsoids (hydrogen atoms are omitted for clarity). Selected bond lengths (Å) and angles (°): [**4**]^+^: Ru1–C1 1.964(6), C1–C2 1.414(8), C2–C3 1.413(8), C3–O1 1.271(7), Ru1–O1 2.127(4), C1–O2 1.339(7), C1–Ru1–O1 79.4(2), P1–Ru1–P2 72.07(5); [**5**]^+^: Ru1–O1 2.166(2), Ru1–C1 1.967(4), Ru1–P1 2.2311(9), C1–C2 1.375(5), C2–C3 1.441(5), C3–O1 1.267(4), C1–P2 1.794(4), P2–C10 1.802(4), P1–C10 1.858(4), P1–Ru1–C1 89.31(10), C1–Ru1–O1 77.10(12).

The molecular structures for [**4**](ClO_4_) and [**5**](OTf)⋅CH_2_Cl_2_ were determined by X-ray crystallography (Figure 2). The α-metallafuran structure (metal fragment at the α-carbon atom of the original furan) in [**4**]^+^ is apparently a result of a rearranged [HC≡C(C=O)Ph + ^−^OMe] structure on Ru center, therefore the formation of [**4**]^+^ was due to a combination of an alkyne–vinylidene rearrangement of HC≡C(C=O)Ph on the Ru center, followed by a nucleophilic attack by ^−^OMe (originated from the solvent MeOH) and O_ynone_ coordination to Ru (Scheme 3b). The five-membered metallacycle is essentially planar with the sum of interior angle close to 540° (539.5°). The Ru–C and C–C distances in the metallacycle (1.964(6) and 1.413(8)–1.414(8) Å, respectively) revealed the partial double bond character in the Ru–C and C–C bonds and supported the resonance representation (see Scheme 1c). [**5**]^+^ features a neutral tridentate P^C^O pincer ligand [(Ph)_2_*P*CH_2_P(Ph)_2_*C*CHC(Ph)*O*], which could be formed as a result of an alkyne–vinylidene rearrangement of HC≡C(C=O)Ph on the Ru center, followed by a nucleophilic attack by a P_dppm_ atom and O_ynone_ coordination to Ru (Scheme 3b). The [Ru(P^C^O)] moiety is a bicyclic system comprising a planar α-metallafuran (sum of interior angle = 539.9°) fused with a five-membered C^P-chelate ring adopting an envelope conformation with the CH_2_ unit on the dppm as the flap. Again, the Ru–C and C–C distances in the metallacycle (1.967(4) and 1.375(5)–1.441(5) Å, respectively) revealed the partial double bond character in the Ru–C and C–C bonds. 

**Figure 3:**
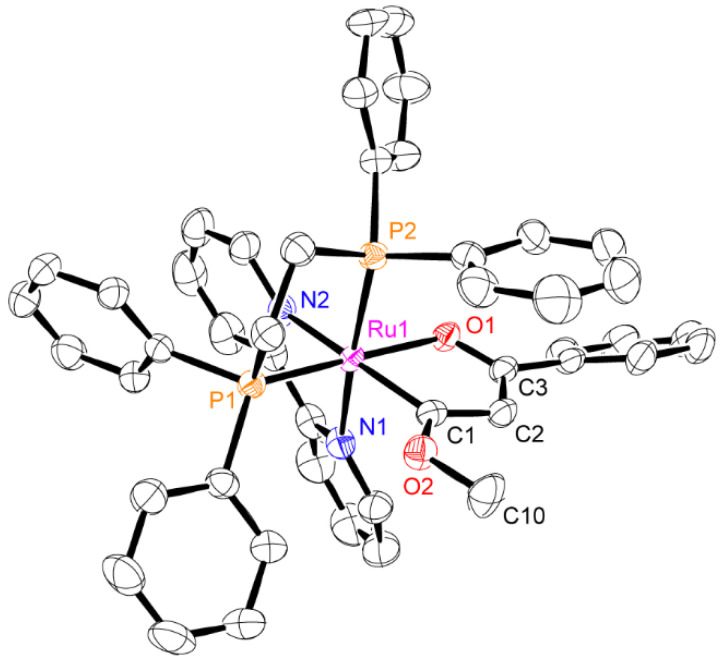
Perspective view of [**7**]^+^ as represented by 50% probability ellipsoids (hydrogen atoms are omitted for clarity). Selected bond lengths (Å) and angles (°): Ru1–C1 1.982(3), C1–C2 1.393(4), C2–C3 1.399(4), C3–O1 1.276(3), Ru1–O1 2.139(2), C1–O2 1.344(4), C1–Ru1–O1 79.20(10), P1–Ru1–P2 84.01(3).

The formation of [**4**]^+^ and [**5**]^+^ in the reaction between HC≡C(C=O)Ph and **1** revealed that there are two competing reactions for the Ru–vinylidene intermediate, namely intermolecular nucleophilic attack by ^−^OMe originated from the solvent MeOH, and intramolecular nucleophilic attack by the auxiliary ligand dppm. However, such competition was not observed in the reaction between HC≡C(C=O)Ph and **2** in MeOH, where methoxy-substituted ruthenafuran [Ru(bpy)(κ^2^-dppe)(C^O)]^+^ ([**7**]^+^) was found to be the only product (Scheme 3a). The difference in reactivity between **1** and **2** may be attributed to their difference in natural bite angle (72° for dppm; 85° for dppe) [48], as bidentate ligands are known to be more labile due to the ring strain. The molecular structure for [**7**](OTf)⋅CH_2_Cl_2_ was also determined by X-ray crystallography (Figure 3); the structural parameters on the metallafuran moiety (Ru–C, C–C, and C–O distances are 1.982(3), 1.393(4)–1.399(4), and 1.276(3) Å, respectively; sum of interior angle = 539.8°) were found to be very similar to those in [**4**]^+^.

Attempts were made to improve the yield of the phosphonium-ring-fused bicyclic ruthenafuran [**5**]^+^ by changing the reaction solvent from MeOH to THF. While no reaction was observed between HC≡C(C=O)Ph and **1** in dry THF, performing the reaction in a mixture of THF and H_2_O led to the formation of a mixture of [**5**]^+^ and carbonyl complex [Ru(bpy)(κ^2^-dppm)(CO)(Cl)]^+^ ([**6**]^+^, Scheme 3a). The role of H_2_O in this reaction is unknown, and the carbonyl complex is likely a result of an oxidative cleavage of a vinylidene species [49,50]. The reaction between HC≡C(C=O)Ph and **2** in the THF/H_2_O mixture gave an analogous carbonyl complex [Ru(bpy)(κ^2^-dppe)(CO)(Cl)]^+^ ([**8**]^+^) as the sole product. Both [**6**]^+^ and [**8**]^+^ featured ν_CO_ (1994 and 1983 cm^–1^, respectively) and ^13^C NMR signal (202 ppm) in the range typical for terminal carbonyl ligands.

### 2.3. Cytotoxicity Study

The serendipitous discovery of the platinum-based anticancer complex cisplatin (*cis*-[Pt(NH_3_)_2_Cl_2_]) and its successful approval from FDA for the treatment of cancer initiated the exploration of new metal-based chemotherapeutics. Among many metal-based anticancer agents being studied, Ru-based complexes have been proven to be promising alternatives to platinum-based drugs [51,52,53,54,55,56,57,58,59,60,61,62,63]. Unlike cisplatin and its derivatives, the six coordination sites of Ru complexes allow more flexibility in the design of coordination sphere for maximizing the overall efficacy of the drugs. In addition, Ru-based anticancer agents have shown far less drug resistance against different types of cancer cells than their Pt counterparts. Motivated by these reasons, the in vitro cytotoxicity of the ruthenafuran [**4**](OTf), [**5**](OTf) and [**7**](OTf) against cervical carcinoma (HeLa) cancer cell line was evaluated by CCK8 assay and benchmarked with cisplatin and the previously reported phosphonium-ring-fused bicyclic ruthenafuran complex [Ru(κ^2^-dppm)(P^C^O)Cl](OTf) ([**9**](OTf)). Interestingly, all the ruthenafuran complexes exhibited moderate to strong cytotoxicity, with IC_50_ values ranging from 0.8 to 2.8 μM (Table 1). Compared with cisplatin (IC_50_ = 21.8 μM), the ruthenafuran complexes are one order of magnitude more cytotoxic. It is worth noting that phosphonium-ring-fused ruthenafurans ([**5**]^+^ and [**9**]^+^) showed stronger cytotoxicity than those without phosphonium moiety. 

## 3. Materials and Methods

### 3.1. General Procedures

**Figure 4:**
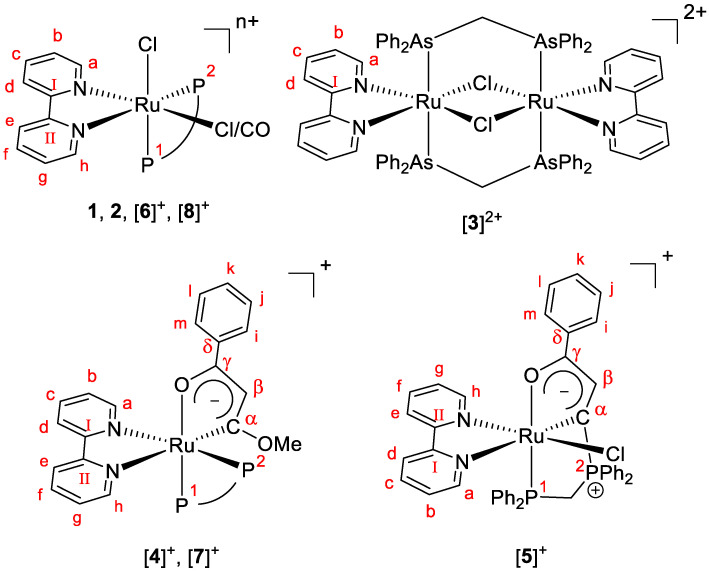
Labeling scheme for H, C and P atoms in this work.

All reactions were performed under an argon atmosphere using standard Schlenk techniques unless otherwise stated. All reagents were used as received, and solvents for reactions were purified by a PureSolv MD5 solvent purification system. *cis*(Cl), *cis*(S)-[Ru(bpy)(dmso-*S*)_2_Cl_2_] (bpy = 2,2′-bipyridine; dmso = dimethyl sulfoxide) were prepared in accordance with the literature methods [64]. ^1^H, ^1^H{^31^P}, ^31^P{^1^H}, ^13^C{^1^H}, ^1^H–^1^H COSY, ^1^H–^1^H NOESY, ^1^H–^13^C HSQC, ^1^H–^31^P HMBC and ^1^H–^13^C HMBC NMR spectra were recorded on Bruker 600 AVANCE III FT-NMR spectrometer. Peak positions were calibrated with solvent residue peaks as internal standard. The ^31^P{^1^H} NMR spectra were referenced to external P(C_6_H_5_)_3_ (−4.7 ppm) [65]. Labeling scheme for H, C and P atoms in the NMR assignments is shown in Figure 4. Electrospray mass spectrometry was performed on a PE-SCIEX API 3200 triple quadrupole mass spectrometer, and the reported or simulated mass values correspond to the most abundant isotopic peaks in the experimental or simulated spectra, respectively. Elemental analyses were performed on an Elementar Vario Micro Cube carbon–hydrogen–nitrogen elemental microanalyzer. Fourier transform infrared (FTIR) spectra were recorded at room temperature using Pekin Elmer “Spectrum 100” FTIR Spectrometer. HeLa (human cervical carcinoma) cell line was preserved by our laboratory. Fetal bovine serum (FBS), phosphate-buffered saline (PBS), penicillinstreptomycin (PS), trypsin-EDTA and Dulbecco’s modified Eagle’s medium (DMEM) were purchased from Gibco BRL (Gaithersburg, MD, U.S.A.). Dimethyl sulfoxide (DMSO, >99.8%) was obtained from Acros Organics and the cell counting kit-8 (CCK8) containing the active chemical WST-8 ([2-(2-methoxy-4-nitrophenyl)-3-(4-nitrophenyl)-5-(2,4-disulfophenyl)-2H-tetrazolium] sodium salt) was purchased from Beyotime.

### 3.2. Synthesis

#### 3.2.1. Synthesis of **1**, **2** and [**3**]^2+^

Method 1: A mixture of *cis*(Cl), *cis*(S)-[Ru(bpy)(dmso-S)_2_Cl_2_] (0.825 mmol) and 1,1-bis(diphenylphosphino)methane (dppm)/1,2-bis(diphenylphosphino)ethane (dppe)/1,1-bis(diphenylarsino)methane (dpam) (0.825 mmol) was refluxed in EtOH (36 mL) under argon for 3 h. During refluxing, the color of the solution changed from orange to red, accompanied with the formation of red precipitates. Upon cooling to room temperature, the resultant red precipitate was collected by suction filtration, washed with cold EtOH (5 mL × 3) and finally Et_2_O (10 mL × 3). These precipitates were pure enough for further reactions. Analytically pure **1**, **2** and [**3**](Cl)_2_ red crystals were obtained by the recrystallization of the precipitates via layering of *n*-hexane onto the CH_2_Cl_2_ solutions of the complexes. 

Method 2: A mixture of *cis*(Cl), *cis*(S)-[Ru(bpy)(dmso-S)_2_Cl_2_] (0.825 mmol) and dppm/dppe/dpam (0.825 mmol) was refluxed in MeOH (50 mL) under argon for 16 h. During refluxing, the color of the solution changed from orange to deep red. Upon cooling to room temperature and the removal of all solvent by reduced pressure, the reaction mixture was dissolved in a minimum amount of CH_2_Cl_2_ and added to Et_2_O (300 mL) to yield red precipitates. These precipitates were pure enough for further reactions and could further be recrystallized as mentioned in Method 1.

**1**. Yields: 92%. Anal. Calcd for C_35_H_30_Cl_2_N_2_P_2_Ru: C, 59.00; H, 4.24; N, 3.93. Found: C, 59.12; H, 4.25; N, 3.94. ^1^H{^31^P} NMR (600 MHz, CD_2_Cl_2_): δ 4.58–4.60, 5.28–5.31 (m, 2H, *CH*_2_ on P_1_^P_2_), 6.26–6.28 (m, 1H, H_b_), 7.48–7.50 (m, 1H, H_c_), 7.50–7.51 (m, 1H, H_a_), 7.54–7.56 (m, 1H, H_g_), 7.95–7.97 (m, 1H, H_f_), 7.99–8.01 (m, 1H, H_d_), 8.19–8.21 (m, 1H, H_e_), 9.94–9.95 (m, 1H, H_h_), 7.34–7.36, 7.35–7.37, 7.38–7.41, 7.75–7.76, 8.19–8.21 (m, 10H, protons of Ph rings on P_2_), 6.75–6.76, 6.93–6.96, 7.09–7.11, 7.21–7.24, 7.28–7.31 (m, 10H, protons of Ph rings on P_1_). ^13^C{^1^H} NMR (151 MHz, CD_2_Cl_2_): δ 44.23 (*C*H_2_ on P_1_^P_2_), 122.03 (C_e_), 122.47 (C_d_), 124.50 (C_b_), 125.23 (C_g_), 134.91 (C_c_ ), 136.64 (C_f_), 152.39 (C_h_), 155.99 (C_a_), 156.72 (C_II_), 158.93 (C_I_), 128.04, 129.82, 130.04, 131.78, 133.66, 134.23, 136.20 (12C of Ph rings on P_2_), 128.33, 128.63, 129.60, 129.94, 130.75, 132.14, 134.50 (12C of Ph rings on P_1_). ^31^P{^1^H} NMR (243 MHz, CD_2_Cl_2_): δ 2.66 (d, *J*_P2P1_ = 67.2 Hz, P_2_), 10.11 (d, *J*_P1P2_ = 67.2 Hz, P_1_), ESI-MS *m*/*z* calcd. for C_35_H_30_ClN_2_P_2_Ru ([**1** − Cl]^+^): 677.1, Found: 677.0.

**2**. Yields: 93%. Anal. Calcd for C_36_H_32_Cl_2_N_2_P_2_Ru: C, 59.51; H, 4.44; N, 3.86. Found: C, 58.98; H, 4.42; N, 3.86. ^1^H{^31^P} NMR (600 MHz, CD_2_Cl_2_): δ 2.48–2.53, 2.65–2.70, 3.03–3.06, 3.32–3.35 (m, 4H, (*CH*_2_)_2_ on P_1_^P_2_), 6.05–6.07 (m, 1H, H_b_), 7.20–7.21 (m, 1H, H_a_), 7.37–7.40 (m, 1H, H_c_), 7.53–7.56 (m, 1H, H_g_), 7.92–7.93 (m, 1H, H_d_), 7.98–8.01 (m, 1H, H_f_), 8.19–8.23 (m, 1H, H_e_), 9.82–9.83 (m, 1H, H_h_), 7.24–7.29, 7.31–7.34, 8.03–8.04, 8.23–8.24 (m, 10H, protons of Ph rings on P_2_), 6.51–6.52, 6.81–6.84, 6.97–6.99, 7.18–7.21, 7.53–7.55 (m, 10H, protons of Ph rings on P_1_). ^13^C{^1^H} NMR (151 MHz, CD_2_Cl_2_): δ 24.35, 26.33 ((*C*H_2_)_2_ on P_1_^P_2_), 121.61 (C_e_), 122.31 (C_d_), 123.50 (C_b_), 125.06 (C_g_), 134.24 (C_c_ ), 136.63 (C_f_), 152.29 (C_h_), 156.73 (C_II_), 156.80 (C_a_), 159.07 (C_I_), 127.31, 128.58, 129.36, 129.78, 132.35, 132.71, 135.32 (12C of Ph rings on P_2_), 127.66, 128.13, 128.63, 129.72, 132.77, 133.57, 135.32 (12C of Ph rings on P_1_). ^31^P{^1^H} NMR (243 MHz, CD_2_Cl_2_): δ 61.38 (d, *J*_P2P1_ = 22.4 Hz, P_2_), 68.42 (d, *J*_P1P2_ = 22.4 Hz, P_1_). ESI-MS m/z calcd. for C_36_H_32_ClN_2_P_2_Ru ([**2** − Cl]^+^): 691.1, Found: 691.2.

[**3**](OTf)_2_. Yields: 97%. Anal. Calcd for C_72_H_60_Cl_2_F_6_N_4_O_6_S_2_As_4_Ru_2_: C, 47.30; H, 3.31; N, 3.06. Found: C, 47.24; H, 3.30; N, 3.05. ^1^H NMR (600 MHz, CD_3_CN): δ 4.00 (s, 4H, *CH*_2_ on dpam), 7.01–7.03 (m, 4H, H_b_), 7.46–7.49 (m, 4H, H_c_), 7.68–7.70 (m, 4H, H_d_), 8.90–8.91 (m, 4H, H_a_), 6.91–6.97, 7.11–7.14 (m, 40H, protons of Ph rings on dpam). ^13^C{^1^H} NMR (151 MHz, CD_3_CN): δ 16.22 (*C*H_2_ on dpam), 124.16 (C_d_), 126.84 (C_b_), 136.64 (C_c_), 154.06 (C_a_), 159.10 (C_I_), 129.66, 130.75, 132.16, 133.08 (48C of Ph rings on dpam). ESI-MS m/z calcd. for C_70_H_60_Cl_2_N_4_As_4_Ru_2_ ([**3**]^2+^): 765.0, Found: 764.9.

#### 3.2.2. Synthesis of [**4**](OTf) and [**5**](OTf)

A mixture of 1-phenylprop-2-yn-1-one (HC≡C(C=O)Ph, 0.14 mmol) and *cis*-[Ru(bpy)(dppm)Cl_2_] (0.07 mmol) were refluxed in MeOH (50 mL) under argon for 16 h, during which the metal precursor gradually dissolved and the color of the reaction mixture changed from red to deep red. Upon cooling to room temperature, a saturated aqueous NaOTf solution (5 mL) was added into the reaction mixture, and the mixture was concentrated to about 5 mL by reduced pressure to give a suspension of brown red solids. The solids were then collected by suction filtration, washed with deionized water (10 mL × 3) and finally Et_2_O (10 mL × 3). The separation of the crude products [**4**](OTf) and [**5**](OTf) was performed by column chromatography. Conditions: basic alumina, CH_2_Cl_2_/(CH_3_)_2_CO 9:1 (*v*/*v*) as eluent gave [**4**](OTf) as the first band (orange); the second band (purple) containing [**5**](OTf) was then eluted with a CH_2_Cl_2_/(CH_3_)_2_CO 7:3 (*v*/*v*) mixture. Analytically pure orange crystals of [**4**](OTf) and deep purple crystals of [**5**](OTf) were obtained by the recrystallization of the collected bands via layering of *n*-hexane onto a CH_2_Cl_2_ solution of the complexes.

[**4**](OTf). Yield: 49%. Anal. Calcd for C_46_H_39_F_3_N_2_O_5_P_2_SRu: C, 58.04; H, 4.13; N, 2.94. Found: C, 58.02; H, 4.12; N, 2.94. ^1^H{^31^P} NMR (600 MHz, (CD_3_)_2_CO): δ 3.43 (s, 3H, OC*H_3_*), 5.09–5.12, 5.35–5.38 (m, 2H, *CH_2_* on P_1_^P_2_), 6.62 (s, 1H, H_β_), 7.08–7.10 (m, 1H, H_g_), 7.34–7.36 (m, 2H, H_l_ + H_j_), 7.42–7.45 (m, 1H, H_k_), 7.55–7.57 (m, 1H, H_b_), 7.71–7.72 (m, 2H, H_i_ + H_m_), 7.96–7.97 (m, 1H, H_h_), 8.64–8.65 (m, 3H, H_a_ + H_d_ + H_e_), 8.09–8.12 (m, 1H, H_f_), 8.14–8.17 (m, 1H, H_c_), 7.26–7.28, 7.36–7.37, 7.38–7.41, 7.43–7.46, 7.64–7.65 (m, 10H, protons of Ph rings on P_2_), 7.04–7.05, 7.17–7.20, 7.38–7.42, 7.51–7.54 (m, 10H, protons of Ph rings on P_1_). ^13^C{^1^H} NMR (151 MHz, (CD_3_)_2_CO): δ 43.12 (*C*H_2_ on P_1_^P_2_), 58.90 (O*C*H_3_), 107.36 (C_β_), 123.84 (C_d_), 124.24 (C_e_), 126.62 (C_g_), 127.18 (C_b_), 128.36 (C_i_ + C_m_), 129.28 (C_l_ + C_j_), 132.15 (C_k_), 137.99 (C_δ_), 139.09 (C_f_), 139.25 (C_c_), 153.86 (C_h_), 155.37 (C_a_), 156.59 (C_II_), 157.31 (C_I_), 198.61 (C_γ_), 255.00 (C_α_), 129.08, 129.23, 130.14, 130.71, 131.40, 131.59, 132.57, 134.09 (12C of Ph rings on P_2_), 129.23, 130.38, 130.71, 131.47, 132.08, 135.93, 136.18 (12C of Ph rings on P_1_). ^31^P{^1^H} NMR (243 MHz, (CD_3_)_2_CO): δ 6.10 (d, *J*_P2P1_ = 70.3 Hz, P_1_), 12.30 (d, *J*_P1P2_ = 70.3 Hz, P_2_). ESI-MS m/z calcd. for C_45_H_39_N_2_O_2_P_2_Ru ([**4**]^+^): 802.8, Found: 803.2.

[**5**](OTf). Yield: 36%. Anal. Calcd for C_45_H_36_ClF_3_N_2_O_4_P_2_SRu: C, 56.52; H, 3.79; N, 2.93. Found: C, 56.41; H, 3.78; N, 2.92. ^1^H{^31^P} NMR (600 MHz, (CD_3_)_2_CO): δ 4.23–4.25, 5.03–5.06 (m, 2H, C*H_2_* on P_1_^P_2_), 6.92–6.94 (m, 1H, H_g_), 7.39–7.41 (m, 2H, H_j_ + H_l_), 7.52–7.55 (m, 1H, H_k_), 7.72–7.74 (m, 1H, H_h_), 7.78–7.82 (m, 1H, H_f_), 7.92–7.94 (m, 1H, H_b_), 8.03–8.04 (m, 2H, H_i_ + H_m_), 8.31–8.34 (m, 1H, H_c_), 8.38–8.39 (m, 1H, H_e_), 8.63–8.64 (m, 1H, H_d_), 9.01 (s, 1H, H_β_), 9.78–9.79 (m, 1H, H_a_), 7.56–7.59, 7.79–7.81, 7.87–7.90, 7.98–8.00, 8.44–8.46 (m, 10H, protons of Ph rings on P_1_), 6.46–6.47, 6.73–6.75, 7.02–7.05, 7.26–7.29, 7.37–7.40, 7.64–7.65 (m, 10H, protons of Ph rings on P_2_). ^13^C{^1^H} NMR (151 MHz, (CD_3_)_2_CO): δ 35.31 (*C*H_2_ on P_1_^P_2_), 124.35 (C_d_), 125.00 (C_e_), 128.06 (C_g_), 128.20 (C_b_), 130.39 (C_j_ + C_l_), 130.41 (C_i_ + C_m_), 134.16 (C_k_), 138.11 (C_δ_), 138.49 (C_f_), 139.77 (C_c_), 144.21 (C_β_), 152.57 (C_a_), 155.82 (C_I_), 155.98 (C_h_), 158.68 (C_II_), 198.55 (C_γ_), 239.91 (C_α_), 120.26, 131.20, 132.21, 135.19, 135.26, 135.67, 135.74, 136.47 (12C of Ph rings on P_1_), 129.63, 129.66, 130.98, 131.78, 131.89, 134.53, 135.19 (12C of Ph rings on P_2_). ^31^P{^1^H} NMR (243 MHz, (CD_3_)_2_CO): δ 25.09–25.39 (m, P_2_), 61.16–61.58 (m, P_1_). ESI-MS *m*/*z* calcd. for C_44_H_36_ClN_2_OP_2_Ru ([**5**]^+^): 807.3, Found: 807.1.

#### 3.2.3. Synthesis of [**5**](OTf) and [**6**](OTf)

The synthesis of [**5**](OTf) and [**6**](OTf) was similar to that of [**4**](OTf), except that a mixture of THF and H_2_O (40 and 10 mL, respectively) was used as the reaction solvent. During refluxing, the metal precursor gradually dissolved and the color of the reaction mixture changed from yellowish red to purple red. Upon cooling to room temperature, the mixture was concentrated to about 10 mL by reduced pressure and the resultant aqueous solution was washed with Et_2_O (10 mL × 2). A saturated aqueous NaOTf solution (5 mL) was added to the aqueous phase, and the resultant mixture was then extracted with CH_2_Cl_2_ (30 mL × 3). The organic phases were dried over anhydrous MgSO_4_. After the removal of MgSO_4_ by a simple filtration, the filtrate was concentrated to give a dark purple red oil. The separation of the crude products [**5**](OTf) and [**6**](OTf) was performed by column chromatography. Conditions: basic alumina, CH_2_Cl_2_/(CH_3_)_2_CO 7:3 (*v*/*v*) as eluent gave [**5**](OTf) as the first band (purple); the second band (yellow) containing [**6**](OTf) was then eluted with a CH_2_Cl_2_/(CH_3_)_2_CO 5:5 (*v*/*v*) mixture. The yield of [**5**](OTf) prepared by this method was found to be 63%.

[**6**](OTf). Yield: 37%. Anal. Calcd for C_37_H_30_ClF_3_N_2_O_4_P_2_SRu: C, 52.03; H, 3.54; N, 3.28. Found: C, 51.91; H, 3.54; N, 3.28. ^1^H{^31^P} NMR (600 MHz, CD_2_Cl_2_): δ 4.84–4.87, 5.63–5.65 (m, 2H, *CH*_2_ on P_1_^P_2_), 6.85–6.86 (m, 1H, H_b_), 7.72–7.74 (m, 1H, H_g_), 7.80–7.81 (m, 1H, H_a_), 7.83–7.86 (m, 1H, H_c_), 8.13–8.15 (m, 1H, H_d_), 8.18–8.21 (m, 1H, H_f_), 8.34–8.35 (m, 1H, H_e_), 9.32–9.33 (m, 1H, H_h_), 7.48–7.49, 7.58–7.62, 7.98–7.99, 8.22–8.23 (m, 10H, protons of Ph rings on P_2_), 6.85–6.86, 7.04–7.07, 7.20–7.22, 7.47–7.48, 7.51–7.53, 7.56–7.57, (m, 10H, protons of Ph rings on P_1_). ^13^C{^1^H} NMR (151 MHz, CD_2_Cl_2_): δ 43.27 (*C*H_2_ on P_1_^P_2_), 124.08 (C_d_), 124.56 (C_e_), 127.04 (C_b_), 127.89 (C_g_), 139.93 (C_f_), 140.00 (C_c_ ), 152.49 (C_a_), 153.44 (C_h_), 155.20 (C_II_), 155.66(C_I_), 202.56 (*C*O), 130.32, 130.55, 131.55, 132.00, 132.81 (12C of Ph rings on P_2_), 128.43, 129.69, 129.75, 130.03, 130.39, 131.87, 131.95, 132.60, (12C of Ph rings on P_1_). ^31^P{^1^H} NMR (243 MHz, CD_2_Cl_2_): δ –9.78 (d, *J*_P2P1_ = 48.5 Hz, P_2_), 1.24 (d, *J*_P1P2_ = 48.5 Hz, P_1_). IR(KBr, cm^–1^): ν_CO_ = 1994. ESI-MS *m*/*z* calcd. for C_36_H_30_ClN_2_OP_2_Ru ([**6**]^+^): 705.1, Found: 705.1.

#### 3.2.4. Synthesis of [**7**](OTf)

The procedure for the synthesis of [**7**](OTf) was the same as that for the synthesis of [**4**](OTf), except that *cis*-[Ru(bpy)(dppe)Cl_2_] was used instead of *cis*-[Ru(bpy)(dppm)Cl_2_]. The color of the reaction mixture was found to be orange during refluxing. The crude product, [**7**](OTf), was eluted as an orange band using a CH_2_Cl_2_/(CH_3_)_2_CO 8:2 (*v*/*v*) mixture as eluent on a basic alumina column. Analytically pure orange crystals of [**7**](OTf) were obtained by the recrystallization of the collected band via layering of *n*-hexane onto a CH_2_Cl_2_ solution of the complex.

[**7**](OTf). Yield: 65%. Anal. Calcd for C_47_H_41_F_3_N_2_O_5_P_2_SRu: C, 58.44; H, 4.28; N, 2.90. Found: C, 58.33; H, 4.26; N, 2.89. ^1^H{^31^P} NMR (600 MHz, CD_2_Cl_2_): δ 2.35–2.39, 2.65–2.69, 3.46–3.52 (m, 4H, (*CH*_2_)_2_ on P_1_^P_2_), 3.21 (s, 3H, OC*H_3_*), 5.97 (s, 1H, H_β_), 6.52–6.54 (m, 1H, H_g_), 7.27–7.33 (m, 2H, H_l_ + H_j_), 7.37–7.39 (m, 1H, H_k_), 7.42–7.46 (m, 2H, H_b_ + H_h_), 7.57–7.58 (m, 2H, H_i_ + H_m_), 7.73–7.75 (m, 1H, H_f_), 8.03–8.06 (m, 1H, H_c_), 8.17–8.19 (m, 1H, H_e_), 8.34–8.35 (m, 1H, H_d_), 8.67–8.68 (m, 1H, H_a_), 6.95–6.97, 7.07–7.10, 7.19–7.21, 7.36–7.39, 7.90–7.92 (m, 10H, protons of Ph rings on P_1_), 6.54–6.58, 6.84–6.86, 6.97–7.00, 7.28–7.34, 7.43–7.48 (m, 10H, protons of Ph rings on P_2_). ^13^C{^1^H} NMR (151 MHz, CD_2_Cl_2_): δ 26.34, 28.88 ((*C*H_2_)_2_ on P_1_^P_2_), 58.51 (O*C*H_3_), 106.86 (C_β_), 123.05 (C_d_), 123.12 (C_e_), 125.33 (C_g_), 126.47 (C_b_), 127.73 (C_i_ + C_m_), 128.36 (C_l_ + C_j_), 131.59 (C_k_), 137.58 (C_δ_), 137.83 (C_f_), 138.79 (C_c_), 154.00 (C_a_), 154.52 (C_h_), 155.50 (C_II_), 156.83 (C_I_), 198.31 (C_γ_), 254.61 (C_α_), 128.24, 129.20, 129.86, 131.59, 133.49, 136.16 (12C of Ph rings on P_1_), 128.64, 128.70, 129.03, 129.68 132.37, 132.43, 134.88, 137.55, (12C of Ph rings on P_2_). ^31^P{^1^H} NMR (243 MHz, CD_2_Cl_2_): δ 67.50 (d, *J*_P1P2_ = 20.3 Hz, P_1_), 74.16 (d, *J*_P2P1_ = 20.3 Hz, P_2_). ESI-MS *m*/*z* calcd. for C_46_H_41_N_2_O_2_P_2_Ru ([**7**]^+^): 816.9, Found: 817.1.

#### 3.2.5. Synthesis of [**8**](OTf)

The procedure for the synthesis of [**8**](OTf) was the same as that for [**6**](OTf), except that *cis*-[Ru(bpy)(dppe)Cl_2_] was used instead of *cis*-[Ru(bpy)(dppm)Cl_2_]. During refluxing, the metal precursor gradually dissolved and the color of the reaction mixture changed from deep yellow to pale yellow. The crude product, [**8**](OTf), was eluted as a yellow band using a CH_2_Cl_2_/(CH_3_)_2_CO 5:5 (*v*/*v*) mixture as eluent on a basic alumina column. Analytically pure yellow crystals of [**8**](OTf) were obtained by the recrystallization of the collected band via layering of *n*-hexane onto a CH_2_Cl_2_ solution of the complex.

[**8**](OTf). Yield: 40%. Anal. Calcd for C_38_H_32_ClF_3_N_2_O_4_P_2_SRu: C, 52.57; H, 3.71; N, 3.23. Found: C, 52.57; H, 3.72; N, 3.24. ^1^H{^31^P} NMR (600 MHz, MeOD): δ 2.90–2.95, 3.07–3.11, 3.33–3.36 (m, 4H, (*CH*_2_)_2_ on P_1_^P_2_), 6.86–6.98 (m, 1H, H_b_), 7.59–7.60 (m, 1H, H_a_), 7.80–7.82 (m, 1H, H_g_), 7.86–7.89 (m, 1H, H_c_), 8.25–8.28 (m, 2H, H_d_ + H_f_), 8.48–8.49 (m, 1H, H_e_), 9.30–9.31 (m, 1H, H_h_), 7.46–7.47, 7.48–7.50, 7.55–7.57, 8.09–8.10, 8.28–8.30 (m, 10H, protons of Ph rings on P_2_), 6.64–6.65, 6.92–6.95, 7.14–7.16, 7.51–7.55, 7.68–7.69 (m, 10H, protons of Ph rings on P_1_). ^13^C{^1^H} NMR (151 MHz, MeOD): δ 24.80, 27.35 ((*C*H_2_)_2_ on P_1_^P_2_), 125.35 (C_d_), 125.70 (C_e_), 127.26 (C_b_), 129.01 (C_g_), 141.05 (C_f_), 141.14 (C_c_ ), 153.50 (C_a_), 156.57 (C_II_), 154.90 (C_h_), 157.21 (C_I_), 202.14 (*C*O), 130.05, 130.60, 132.15, 132.20, 132.21, 133.02, 134.16, 136.12 (12C of Ph rings on P_2_) 130.00, 130.10, 130.54, 130.93, 131.74, 133.09 (12C of Ph rings on P_1_). ^31^P{^1^H} NMR (243 MHz, MeOD): δ 51.09 (d, *J*_P2P1_ = 13.29 Hz, P_2_), 62.71 (d, *J*_P1P2_ = 13.29 Hz, P_1_). IR(KBr, cm^–1^): ν_CO_ = 1983. ESI-MS *m*/*z* calcd. for C_37_H_32_ClN_2_OP_2_Ru ([**8**]^+^): 719.1, Found: 719.0.

### 3.3. X-ray Crystallographic Data

CCDC 2144812–2144816 contain the supplementary crystallographic data for this paper. These data can be obtained free of charge via http://www.ccdc.cam.ac.uk/conts/retrieving.html, accessed on 3 February 2022 (or from the CCDC, 12 Union Road, Cambridge, CB2 1EZ, U.K.; Fax: +44-1223-336033; E-mail: deposit@ccdc.cam.ac.uk).

### 3.4. Cytotoxicity Studies

The cytotoxicity of [**4**](OTf), [**5**](OTf), [**7**](OTf), [**9**](OTf) and cisplatin (*cis*-[Pt(NH_3_)_2_Cl_2_]) against HeLa cancer cells was evaluated using the CCK8 assay [66]. Briefly, HeLa cells were seeded at 4000 cells per well in a 96-well culture microplate using 100 μL 10% FBS and 1% PS DMEM as culture solution and incubated for 12 h at standard incubation conditions for mammalian cells (37 °C, 5% CO_2_, 95% air). Stock solutions of [**4**](OTf), [**5**](OTf), [**7**](OTf) and [**9**](OTf) (100 μM) were prepared using DMSO as solvent, whereas that of cisplatin (100 μM) was prepared using 0.9% (*w*/*v*) saline solution as solvent. A series of concentrations for [**4**](OTf) and [**7**](OTf) (7.8 nM–10 μM), [**5**](OTf) and [**9**](OTf) (0.38 nM–25 μM), and cisplatin (9.5 nM–250 μM) were prepared in solutions with 10% FBS and 1% PS DMEM; 100 μL of each solution was added to each well and the microplate was incubated for 24 h at standard incubation conditions for mammalian cells (37 °C, 5% CO_2_, 95% air). For [**4**](OTf), [**5**](OTf), [**7**](OTf) and [**9**](OTf), the highest concentration of the complex-treated cell culture medium constitutes 0.5% DMSO. For the corresponding control experiments, 0.5% DMSO were used. Afterwards, the complex-containing culture medium was replaced by CCK8 reagent in 10% FBS and 1% PS DMEM (1:10 *v*/*v*) and the microplate was incubated for 2 h. Upon incubation, the microplate was shaken at room temperature for 10 s and absorbance measurement was carried out at 450 nm using a microplate reader. The cytotoxicity of each complex, expressed as IC_50_, was determined by the surviving cells curve after exposure to the complexes for 24 h. Each experiment was repeated three times to obtain the mean values.

## 4. Conclusions

Mononuclear *cis*-[Ru(κ^2^-dppm)(bpy)Cl_2_] (**1**), *cis*-[Ru(κ^2^-dppe)(bpy)Cl_2_] (**2**), and dinuclear [Ru_2_(bpy)_2_(μ-dpam)_2_(μ-Cl)_2_](Cl)_2_ ([**3**](Cl)_2_), were prepared from the reactions between *cis*(Cl), *cis*(S)-[Ru(bpy)(dmso-*S*)_2_Cl_2_] and diphosphine/diarsine ligands dppm, dppe, and dpam, respectively. The reaction between **2** and HC≡C(C=O)Ph in MeOH only led to the formation of methoxy-substituted ruthenafuran [Ru(bpy)(κ^2^-dppe)(C^O)]^+^ ([**7**]^+^), but reacting **1** with HC≡C(C=O)Ph under identical reaction conditions gave a mixture of methoxy-substituted ruthenafuran [Ru(bpy)(κ^2^-dppm)(C^O)]^+^ ([**4**]^+^) and phosphonium-ring-fused bicyclic ruthenafuran [Ru(bpy)(P^C^O)Cl]^+^ ([**5**]^+^). These findings revealed that (1) the functionalized alkyne HC≡C(C=O)Ph was activated by **1** and **2** via a vinylidene-involving pathway, and (2) bidentate ligands with small bite-angles, such as dppm, could induce new reactivity due to a ring strain. Both methoxy-substituted ruthenafuran and phosphonium-ring-fused bicyclic ruthenafuran were found to exhibit moderate-to-strong cytotoxicity against cervical carcinoma (HeLa) cancer cell line. Encouragingly, they were found to be one order of magnitude more cytotoxic than the classic metal-based anticancer agent cisplatin.

## Figures and Tables

**Table 1:** Cytotoxicity (IC_50_, μM) of complexes [**4**](OTf), [**5**](OTf), [**7**](OTf), [**9**](OTf) and *cis*-[Pt(NH_3_)_2_Cl_2_] against a HeLa cancer cell line ^1^.

Complex	IC_50_ (μM)
[**4**](OTf)	2.53 ± 0.40
[**5**](OTf)	1.60 ± 0.20
[**7**](OTf)	2.83 ± 0.45
[**9**](OTf)	0.84 ± 0.08
*cis*-[Pt(NH_3_)_2_Cl_2_]	21.8 ± 1.34

^1^ Maximum complex concentration tested: 10 μM for [**4**](OTf) and [**7**](OTf); 25 μM for [**5**](OTf) and [**9**](OTf); 250 μM for cisplatin.

## Data Availability

Not applicable.

## Supplementary Materials

The following are available online. Figures S1–S69: NMR spectra of all complexes reported in this work; Figures S70–S85: Experimental and simulated mass spectra of all complexes reported in this work.
