# Supplementary material for: Ruthenafuran Complexes Supported by the Bipyridine-Bis(diphenylphosphino)methane Ligand Set: Synthesis and Cytotoxicity Studies
Source: Molecules. 2022 Mar 5;27(5):1709. doi: 10.3390/molecules27051709 (PMC8911682; doi:10.3390/molecules27051709)
Supplement: Supplementary file 1 [file molecules-27-01709-s001.zip › molecules-1605386-supplementary.pdf]

Supplementary Materials

for

Ruthenafuran Complexes Supported by the Bipyridine-Bis(diphenylphosphino)methane Ligand Set: Synthesis and Cytotoxicity Studies

Chi-Fung Yeung [1,2,†], Sik-Him Tang [1,†], Zhe Yang [1,†],Tsun-Yin Li [1], Ka-Kit Li [1], Yuen-Man Chan [1], Hau-Lam Shek [1], Kai-Wa Io [1], King-Ting Tam [1], Shek-Man Yiu [1], Man-Kit Tse [1] and Chun-Yuen Wong [1,2,*]

[1] Department of Chemistry, City University of Hong Kong, Tat Chee Avenue, Kowloon, Hong Kong

[2] State Key Laboratory of Terahertz and Millimeter Waves, City University of Hong Kong, Tat Chee Avenue, Kowloon, Hong Kong

* Correspondence: acywong@cityu.edu.hk; Tel.: +852-3442-6831

† These authors contributed equally to this work.

Table of Contents

	Page
NMR spectra of complex **1**	S3−S11
NMR spectra of complex **2**	S12−S20
NMR spectra of complex [**3**](OTf)$_2$	S21−S26
NMR spectra of complex [**4**](OTf)	S27−S35
NMR spectra of complex [**5**](OTf)	S36−S44
NMR spectra of complex [**6**](OTf)	S45−S53
NMR spectra of complex [**7**](OTf)	S54−S62
NMR spectra of complex [**8**](OTf)	S63−S71
Experimental and simulated mass spectra of [**1**−Cl]$^+$	S72−S73
Experimental and simulated mass spectra of [**2**−Cl]$^+$	S74−S75
Experimental and simulated mass spectra of [**3**]$^{2+}$	S76−S77
Experimental and simulated mass spectra of [**4**]$^+$	S78−S79
Experimental and simulated mass spectra of [**5**]$^+$	S80−S81
Experimental and simulated mass spectra of [**6**]$^+$	S82−S83
Experimental and simulated mass spectra of [**7**]$^+$	S84−S85
Experimental and simulated mass spectra of [**8**]$^+$	S86−S87

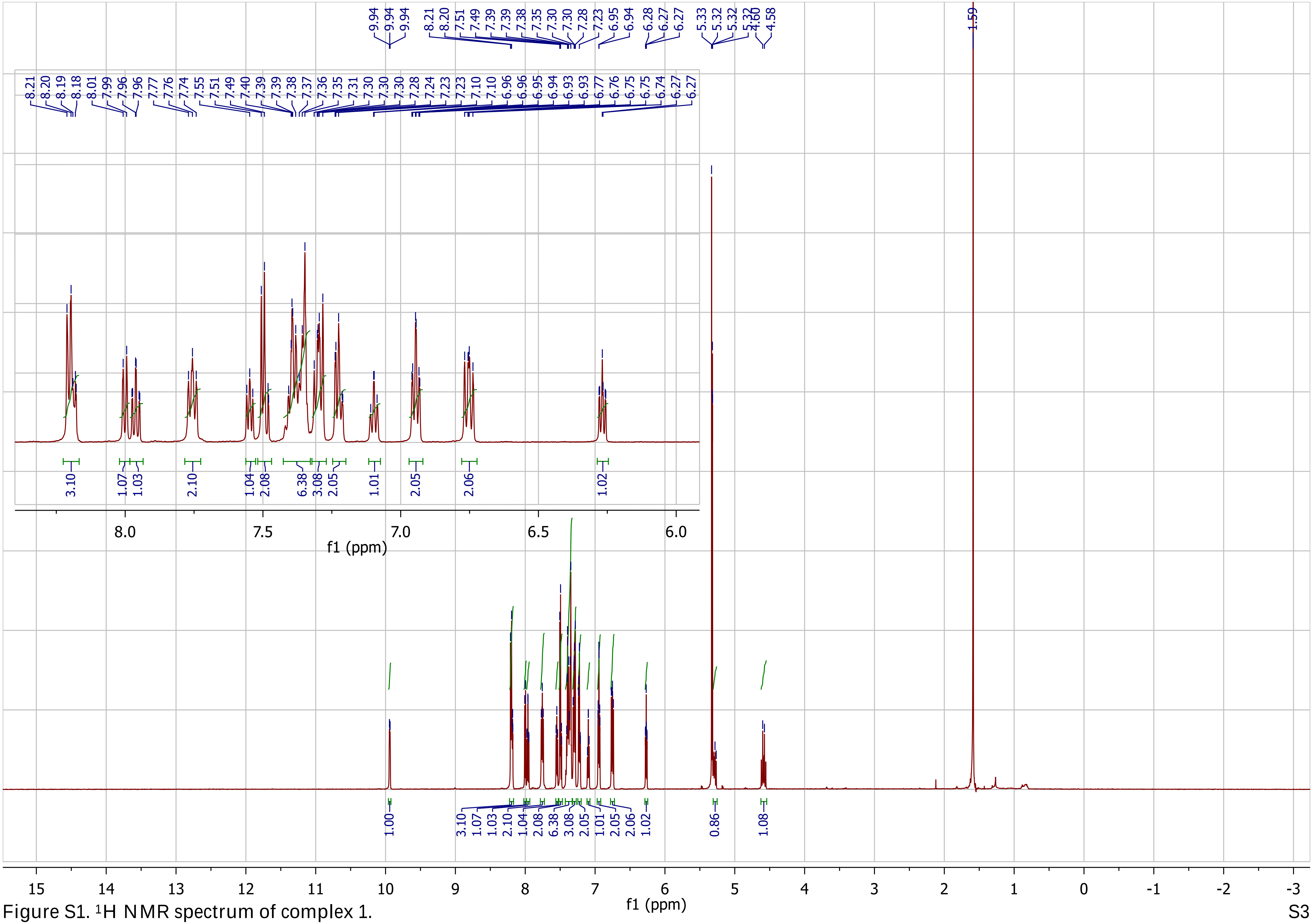

Figure S1. ^1H NMR spectrum of complex 1.

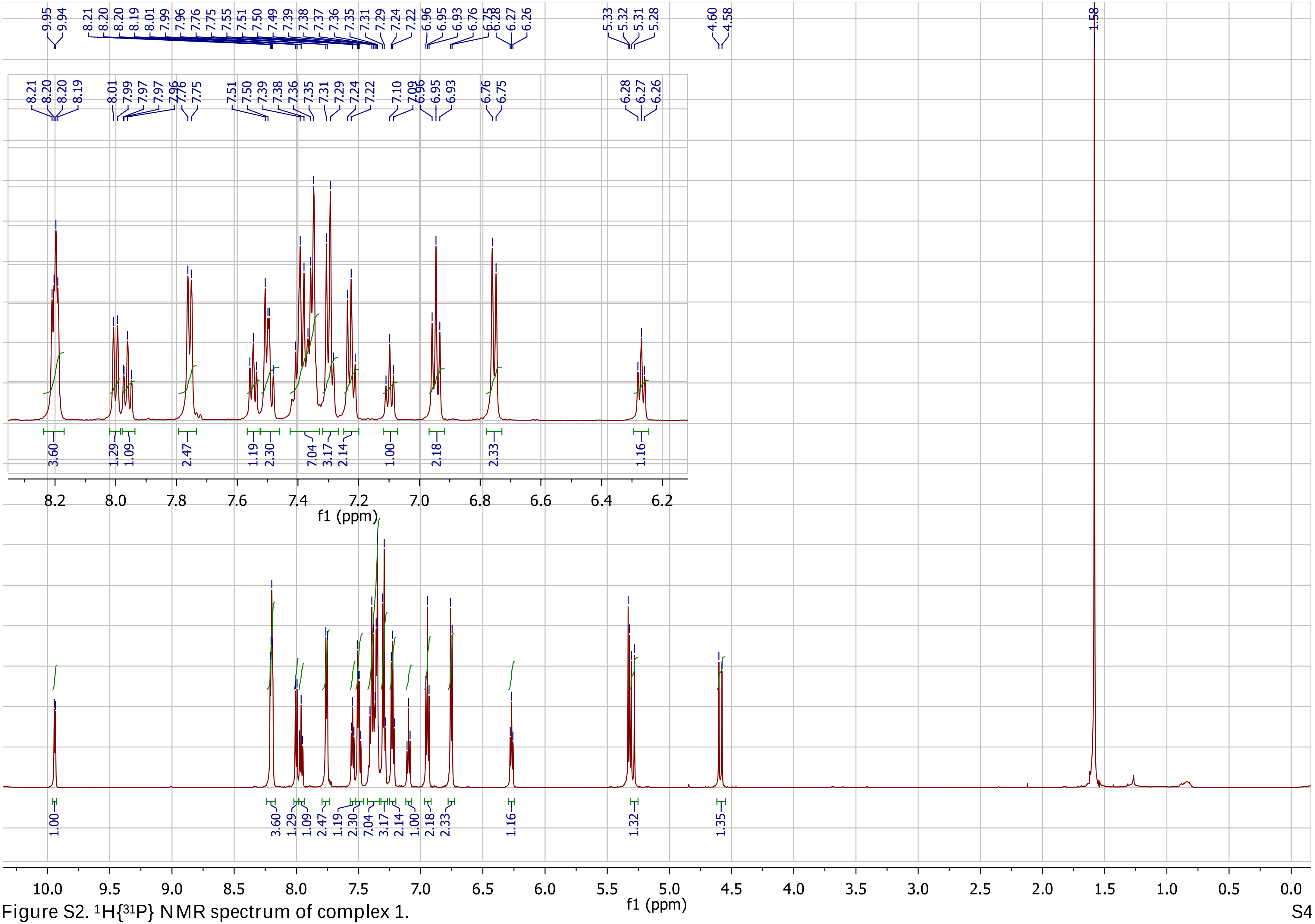

Figure S2. $^{1}H\{^{31}P\}$ NMR spectrum of complex 1.

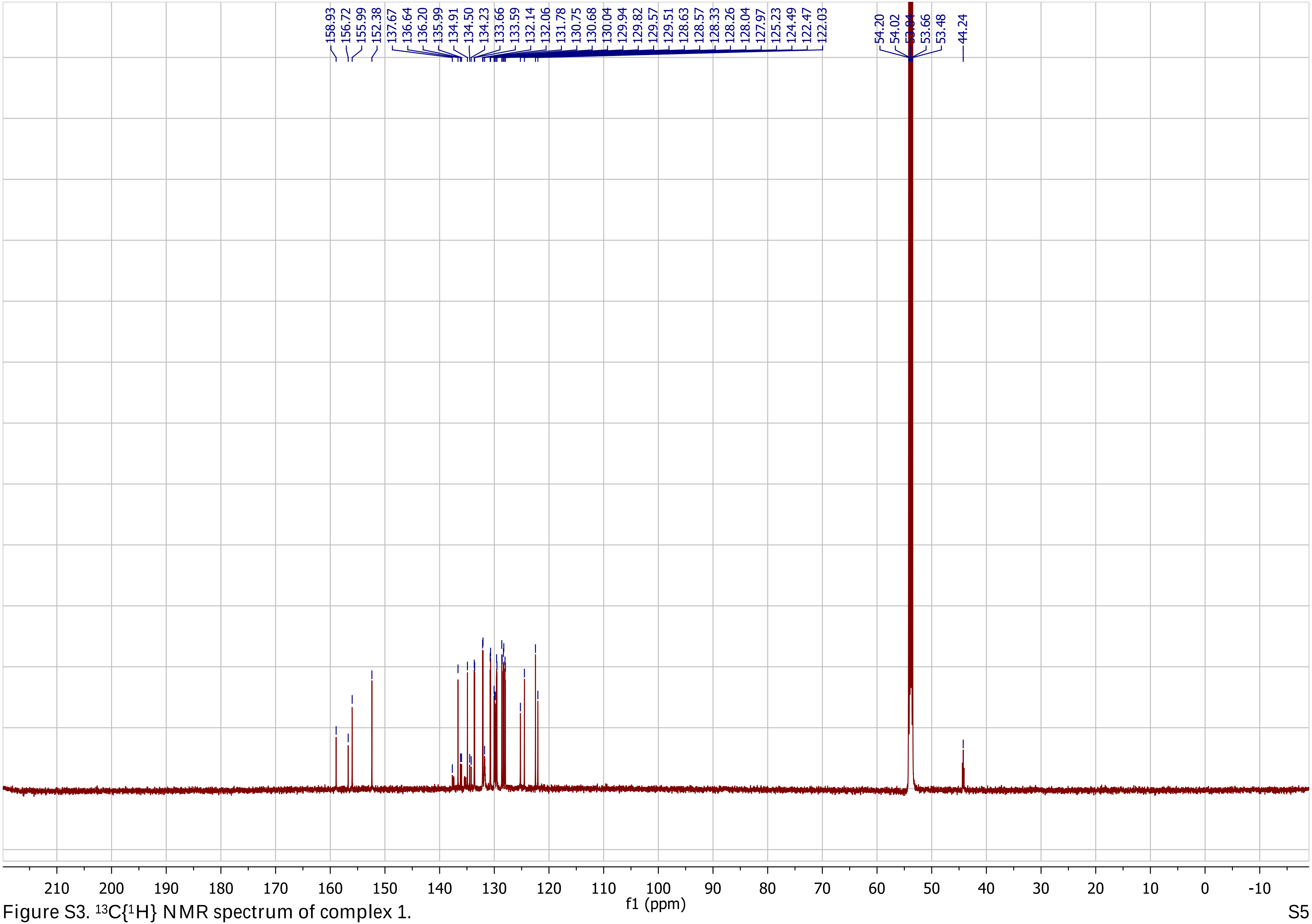

Figure S3. $^{13}C\{^{1}H\}$ NMR spectrum of complex 1.

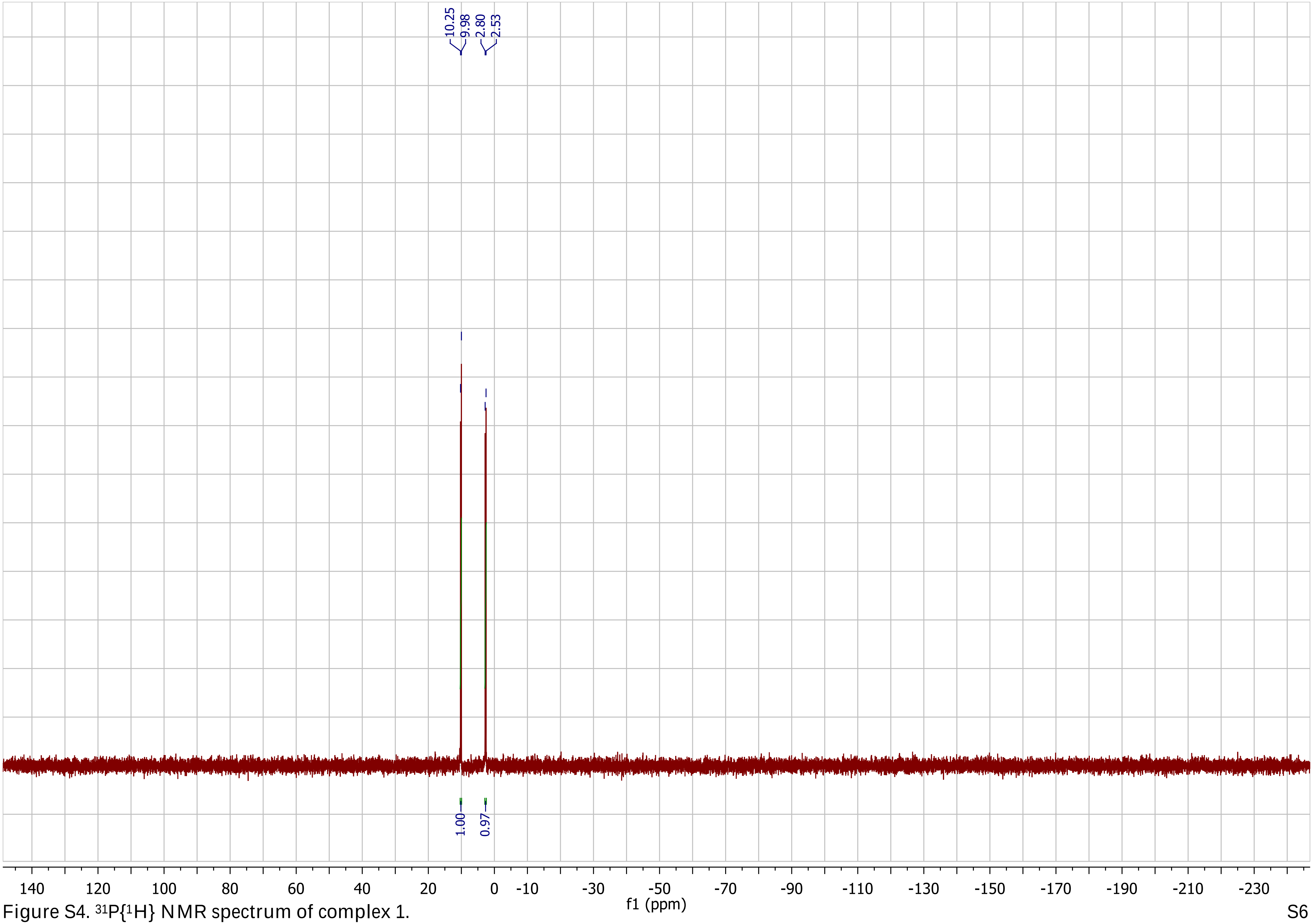

Figure S4. $^{31}P\{^{1}H\}$ NMR spectrum of complex 1.

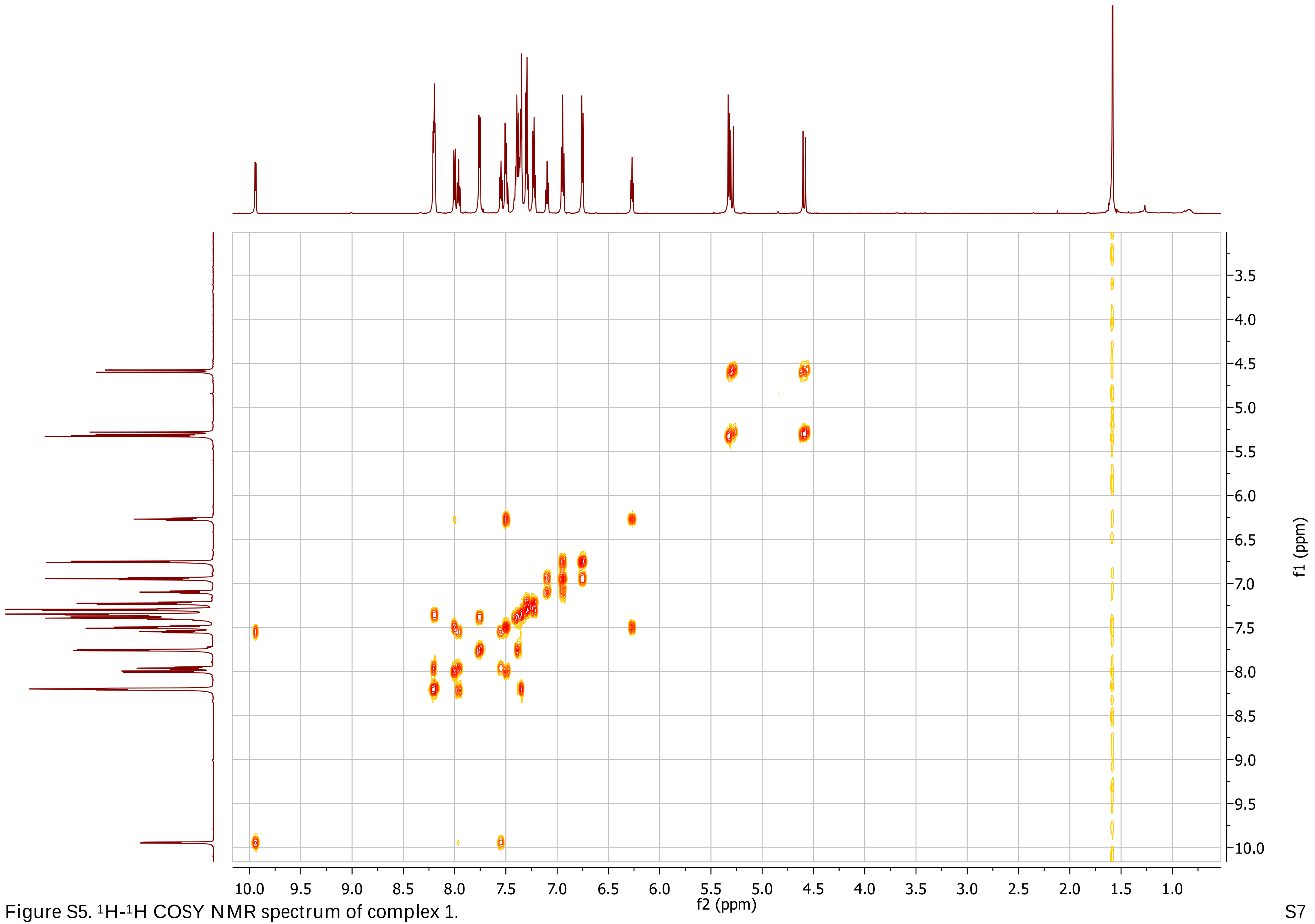

Figure S5. ^{1}H-^{1}H COSY NMR spectrum of complex 1.

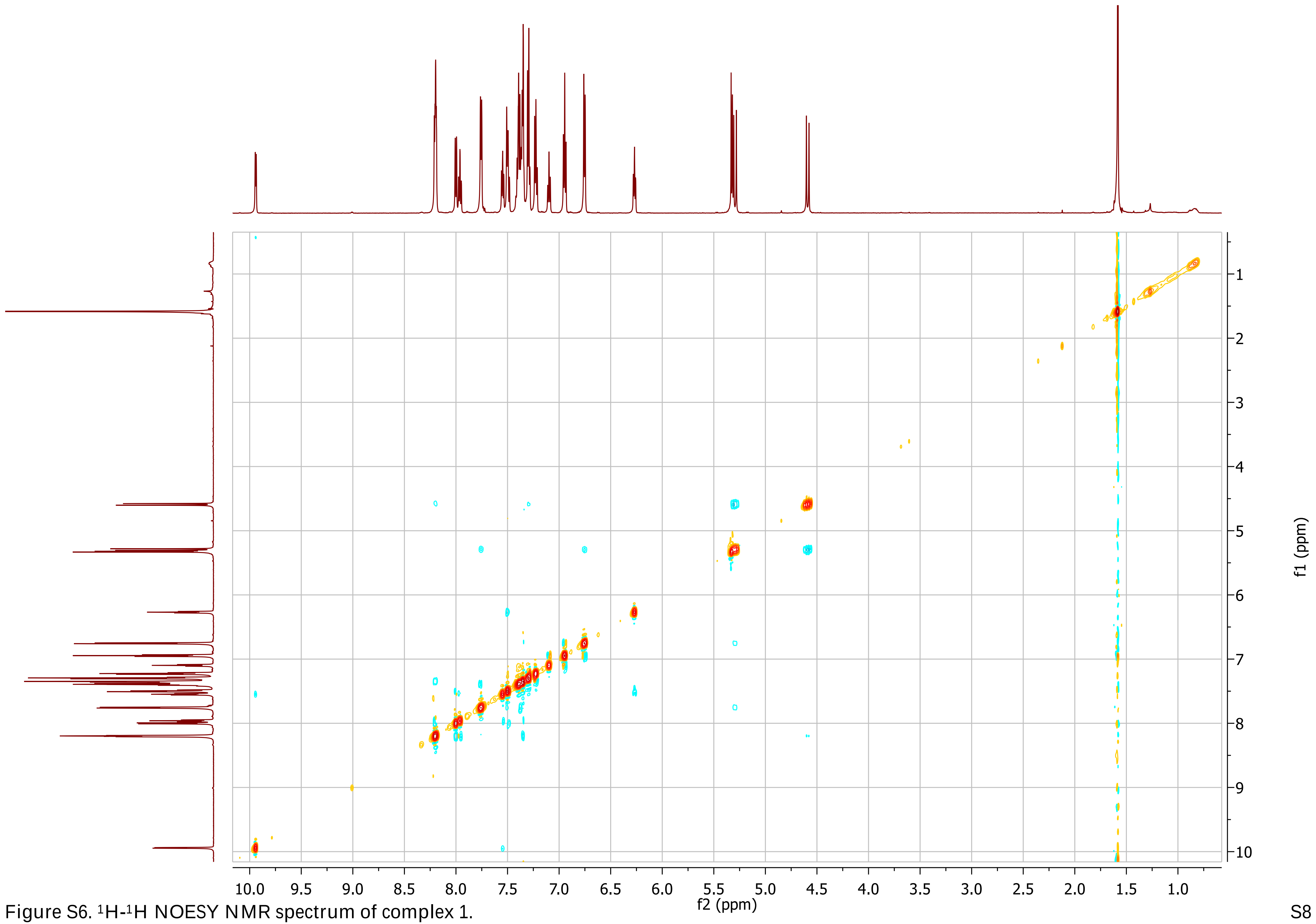

Figure S6. ^{1}H-^{1}H NOESY NMR spectrum of complex 1.

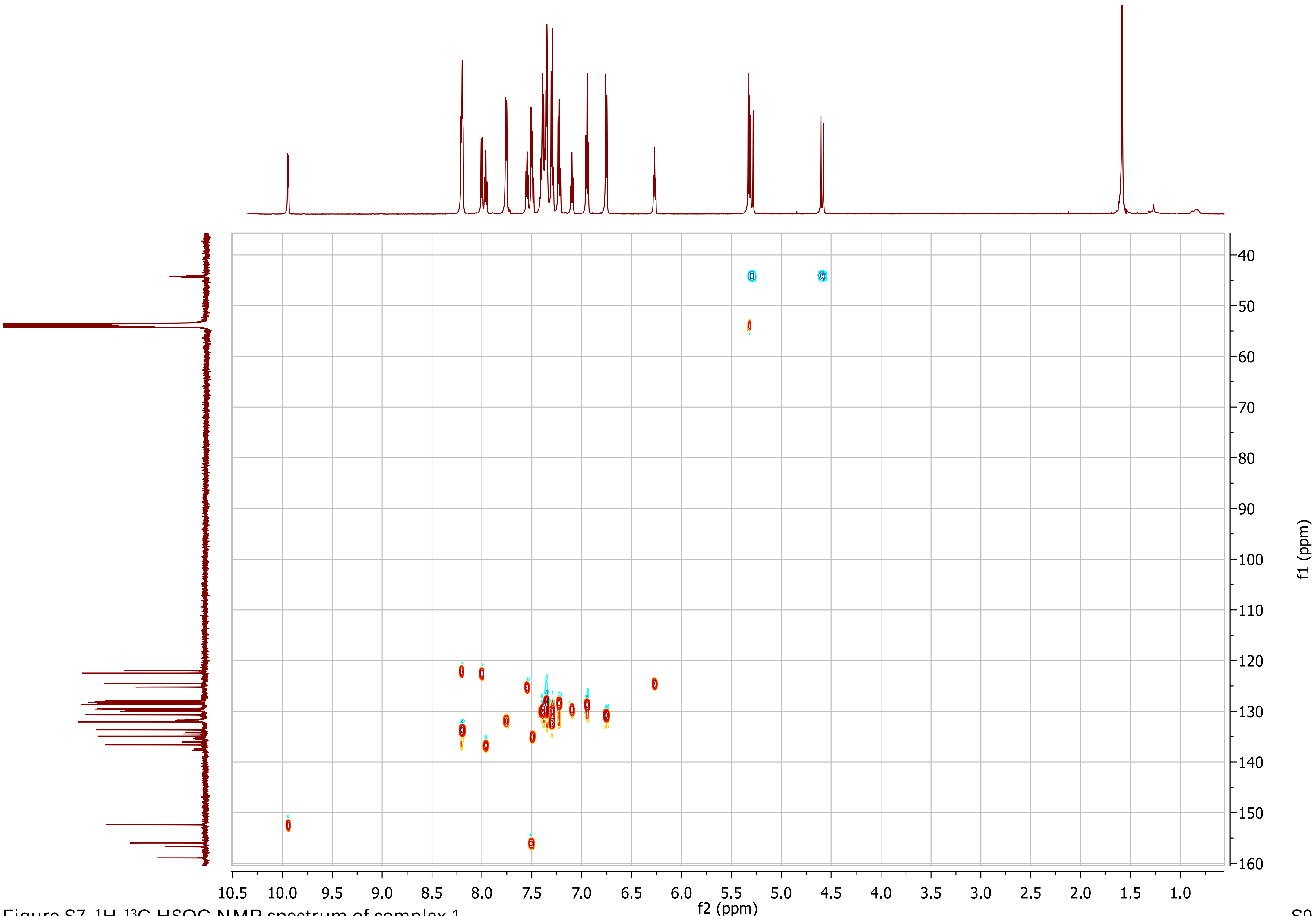

Figure S7. ^{1}H-^{13}C HSQC NMR spectrum of complex 1.

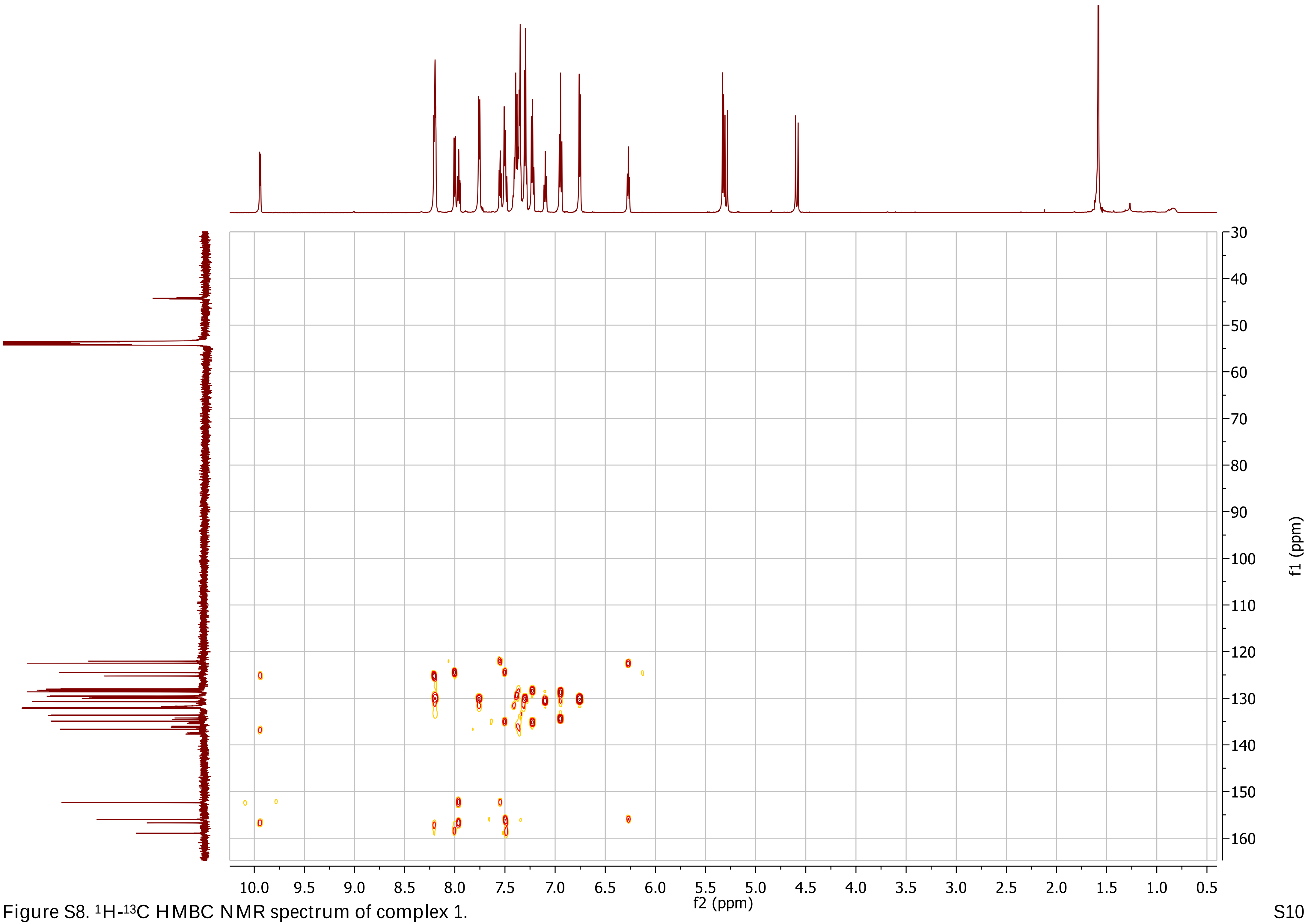

Figure S8. ^{1}H-^{13}C HMBC NMR spectrum of complex 1.

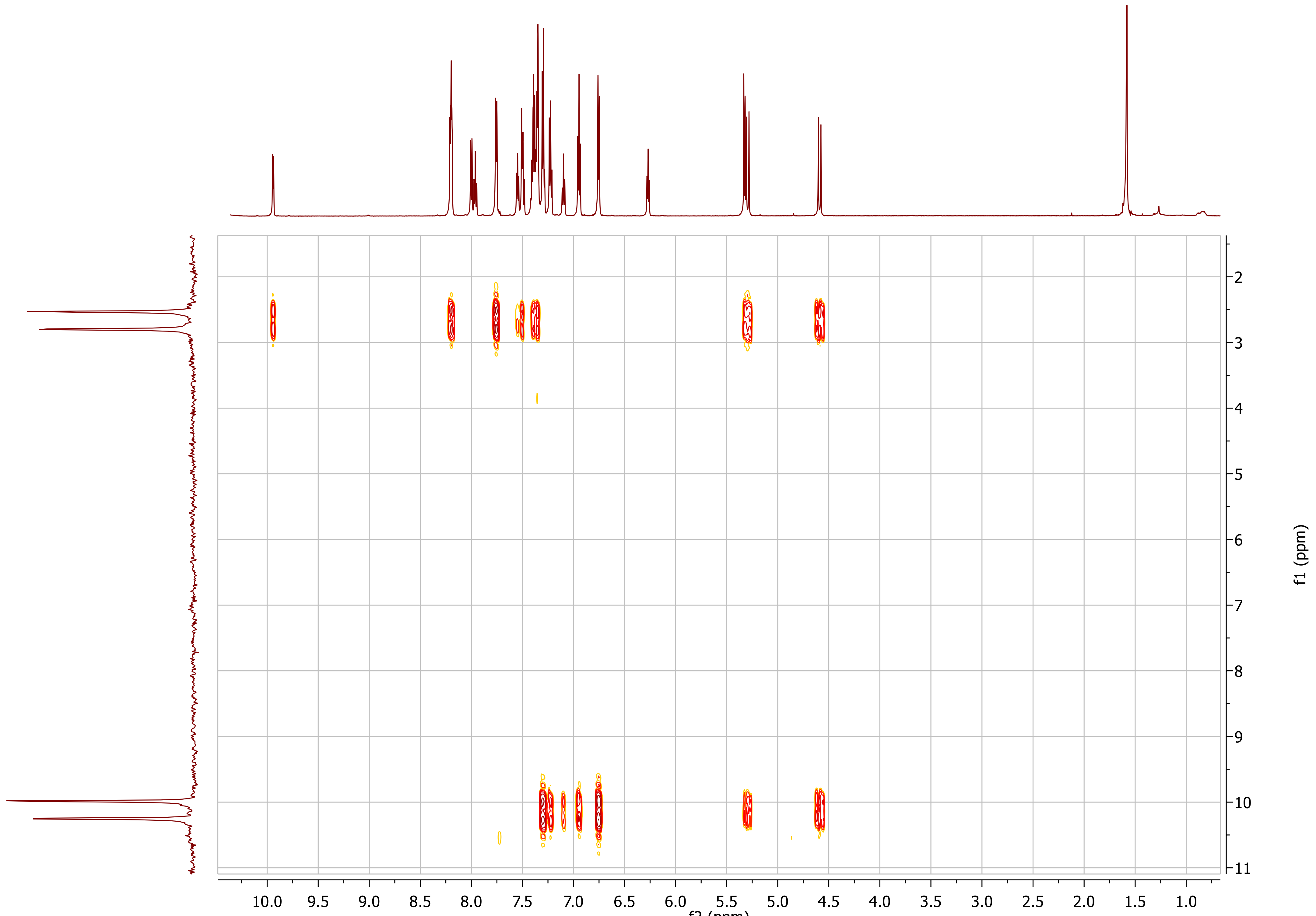

Figure S9. ^{1}H-^{31}P HMBC NMR spectrum of complex 1.

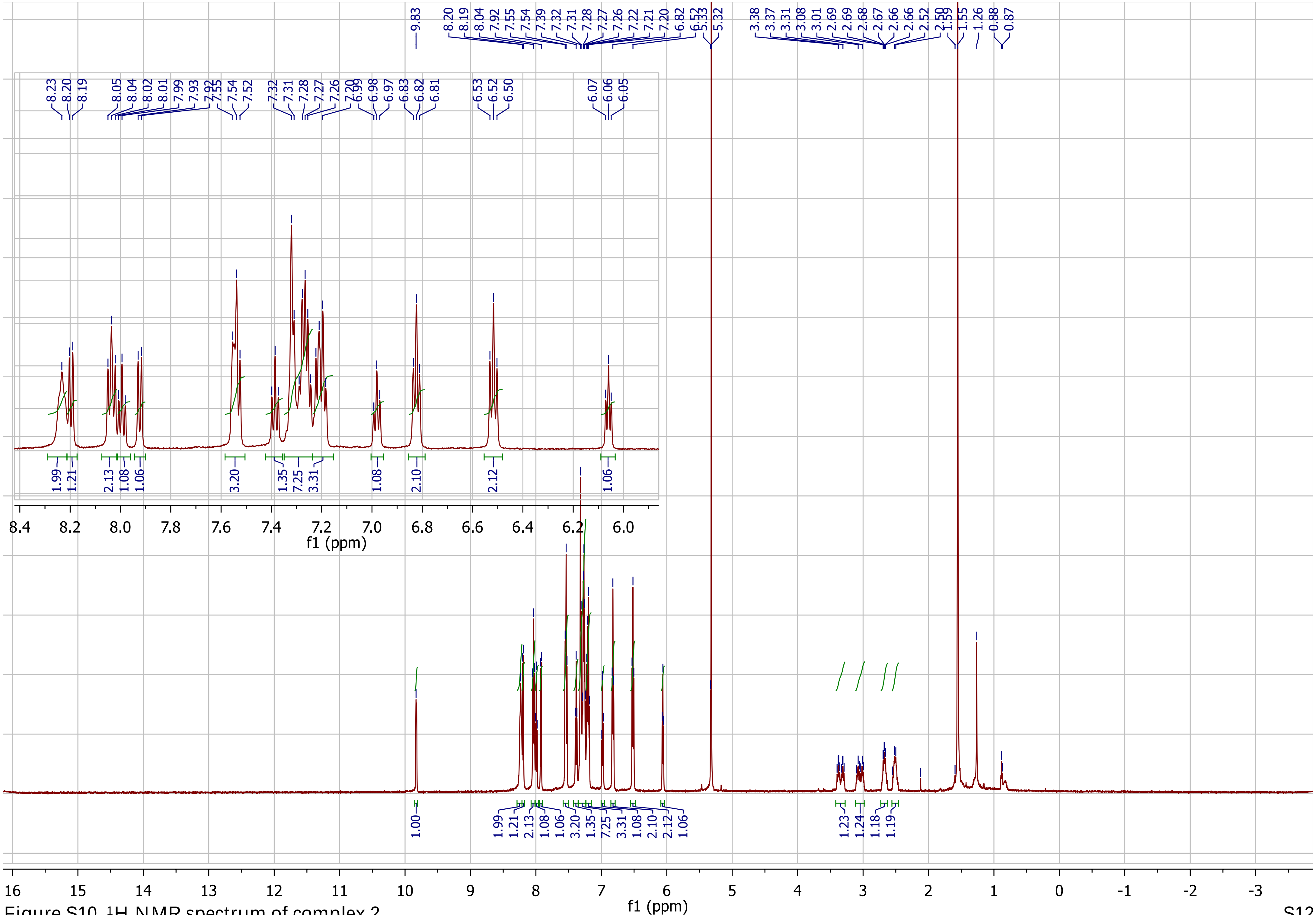

Figure S10. ¹H NMR spectrum of complex 2.

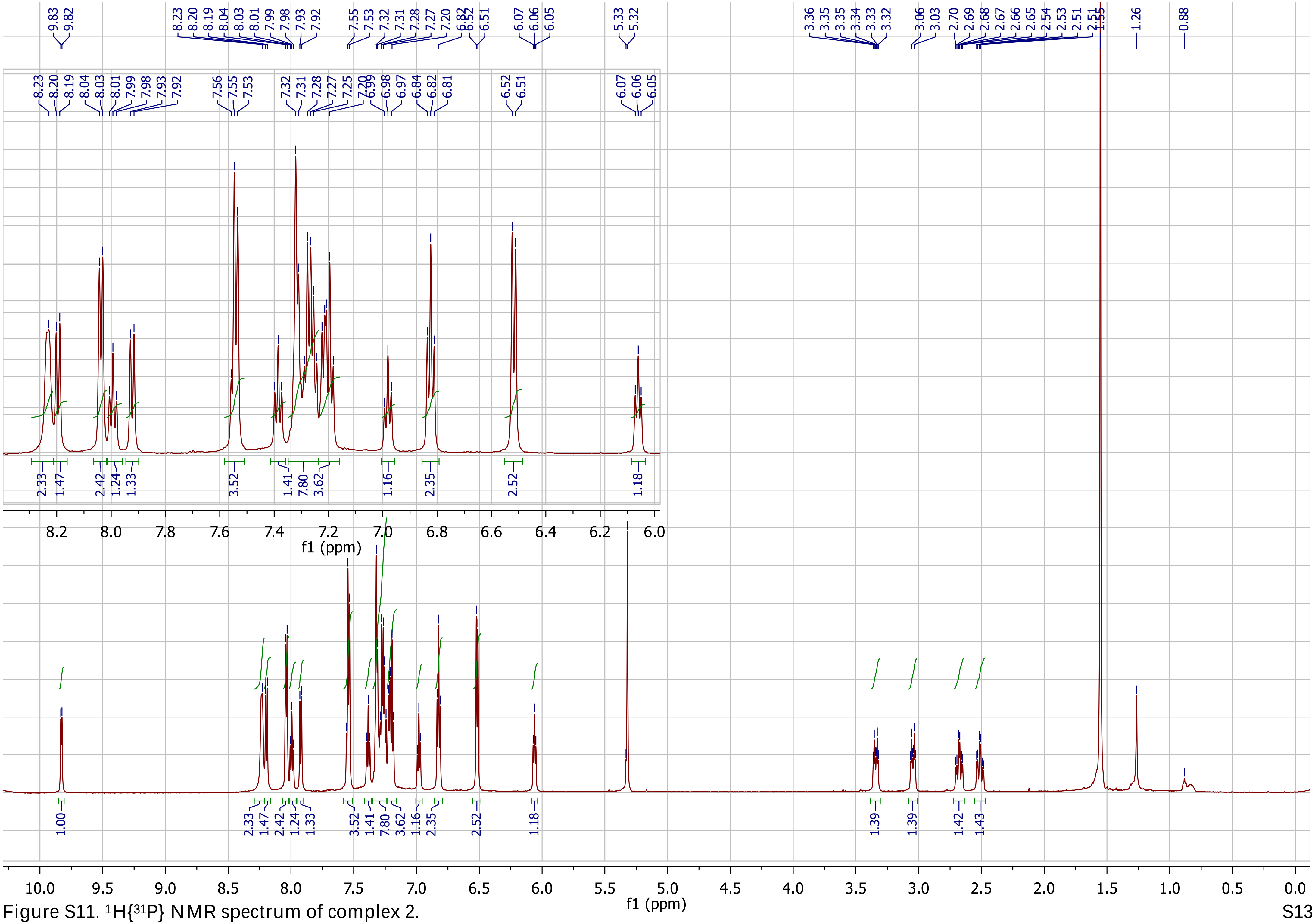

Figure S11. $^{1}H\{^{31}P\}$ NMR spectrum of complex 2.

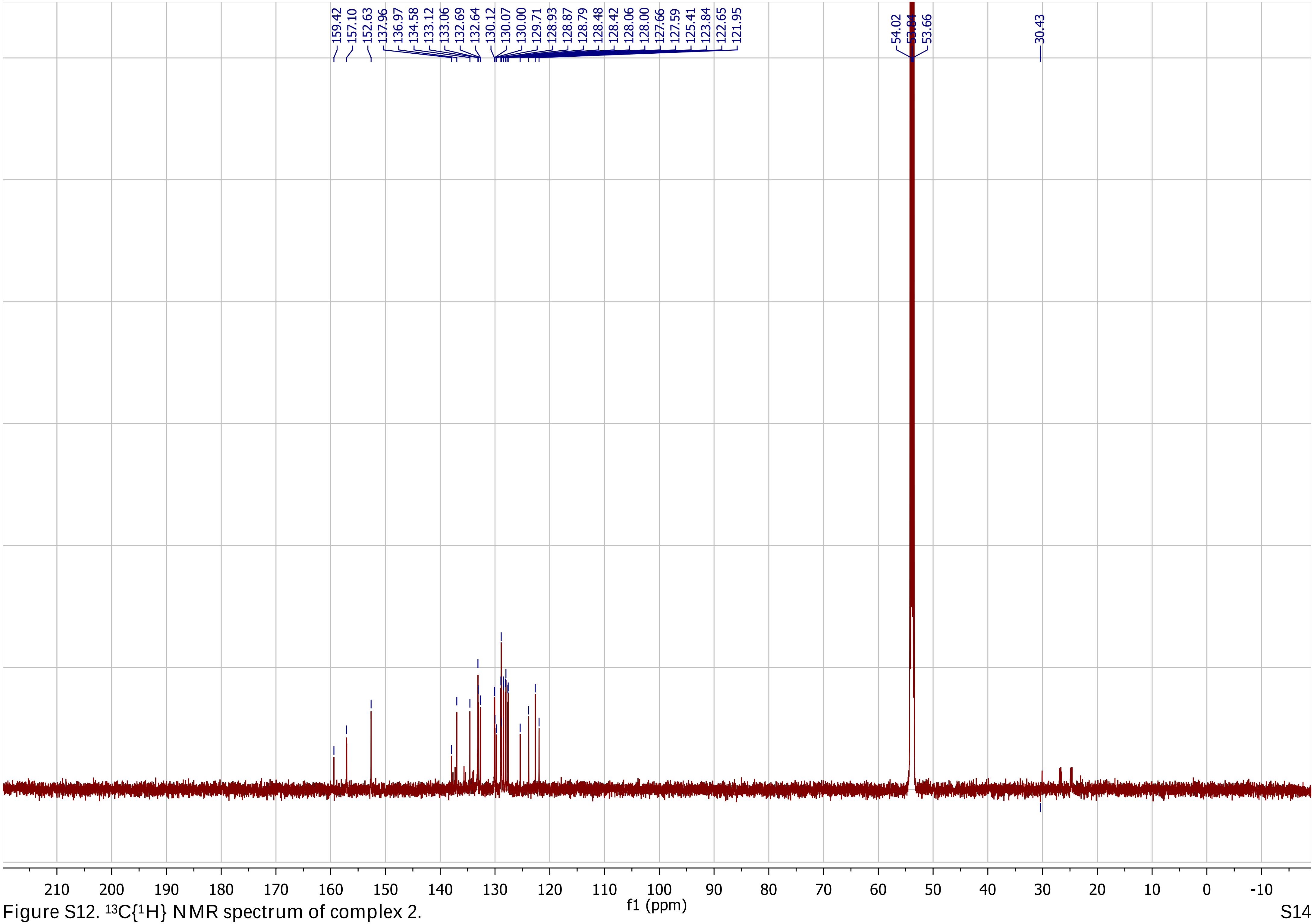

Figure S12. $^{13}C\{^1H\}$ NMR spectrum of complex 2.

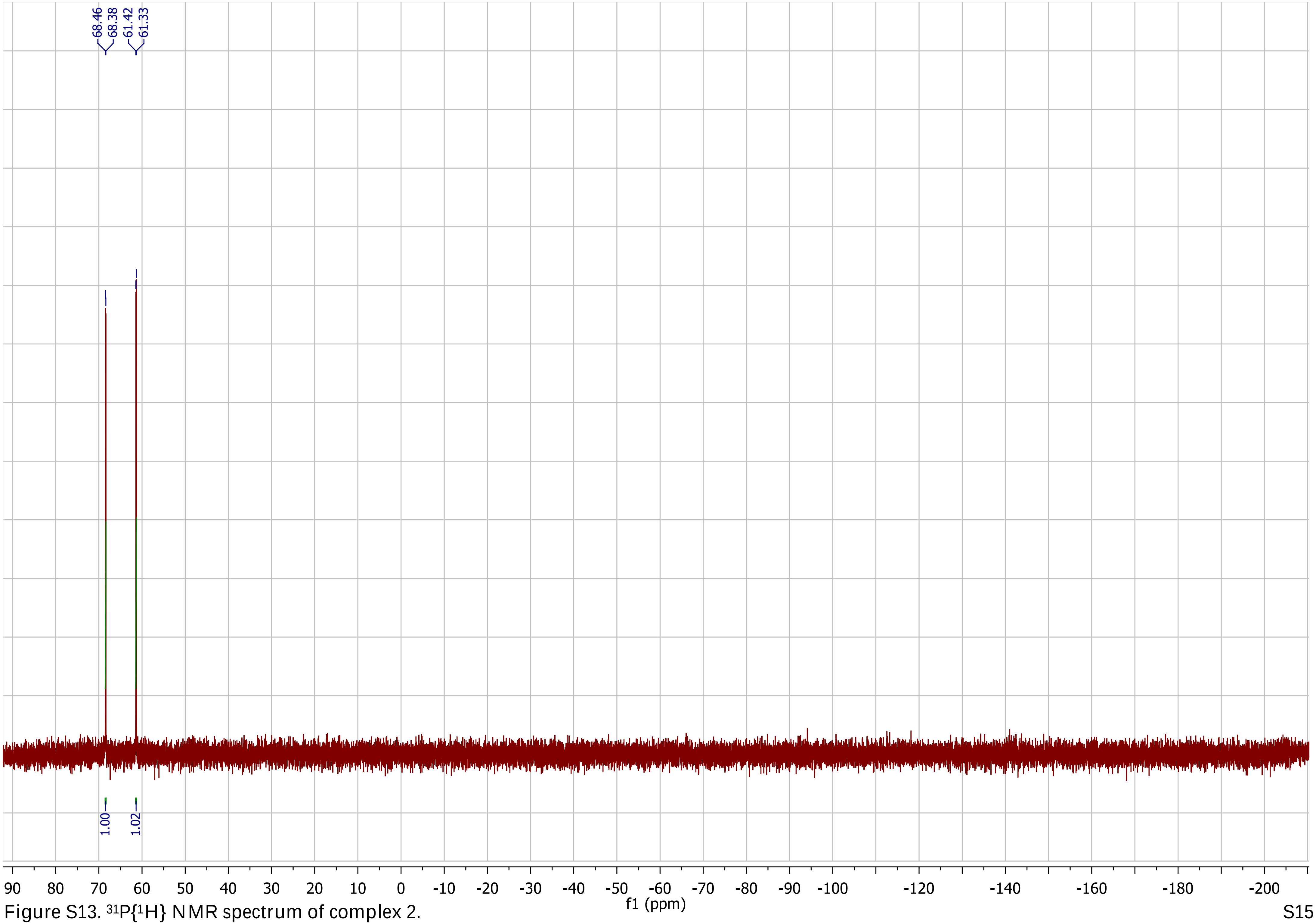

Figure S13. $^{31}P\{^1H\}$ NMR spectrum of complex 2.

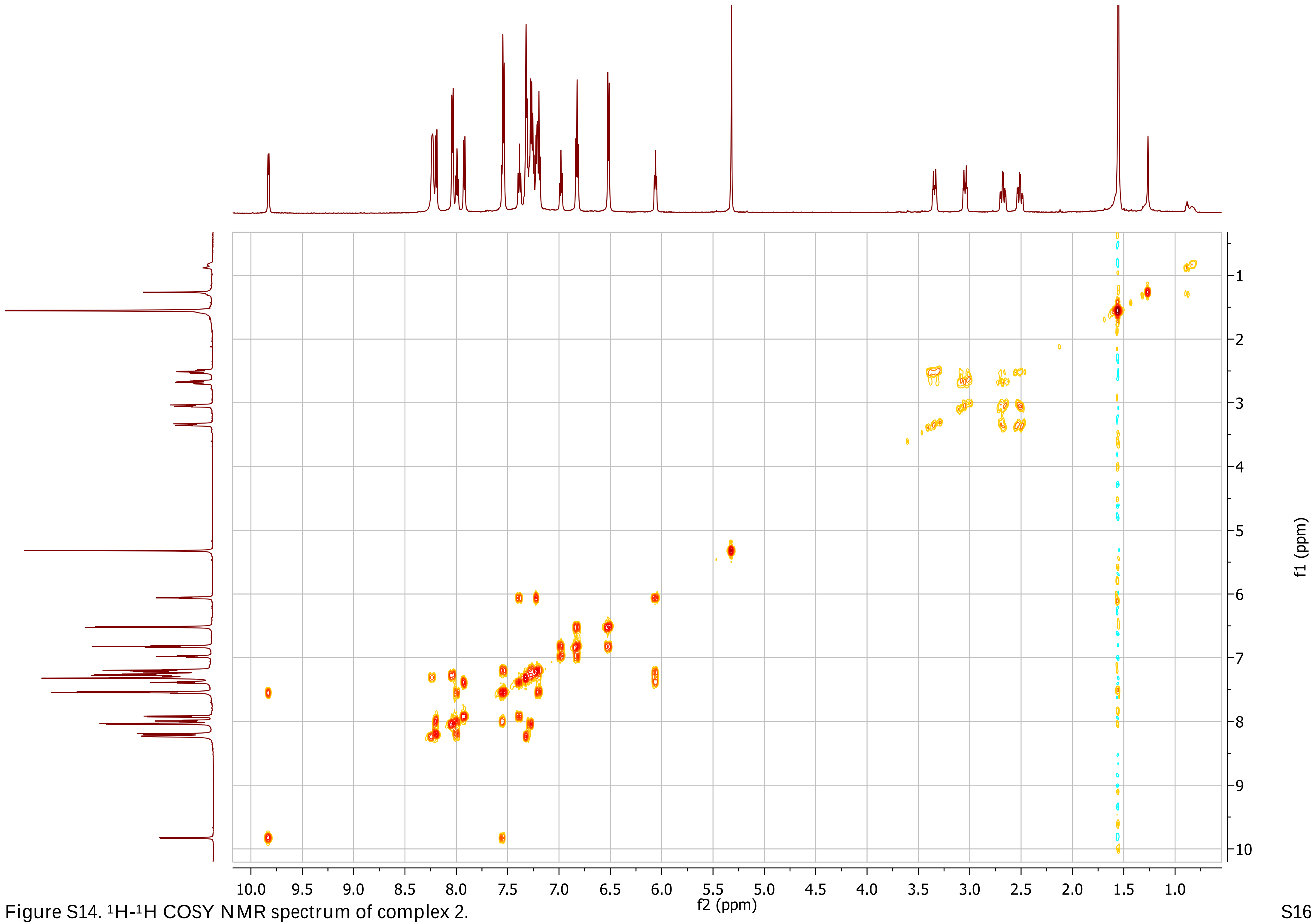

Figure S14. ^{1}H-^{1}H COSY NMR spectrum of complex 2.

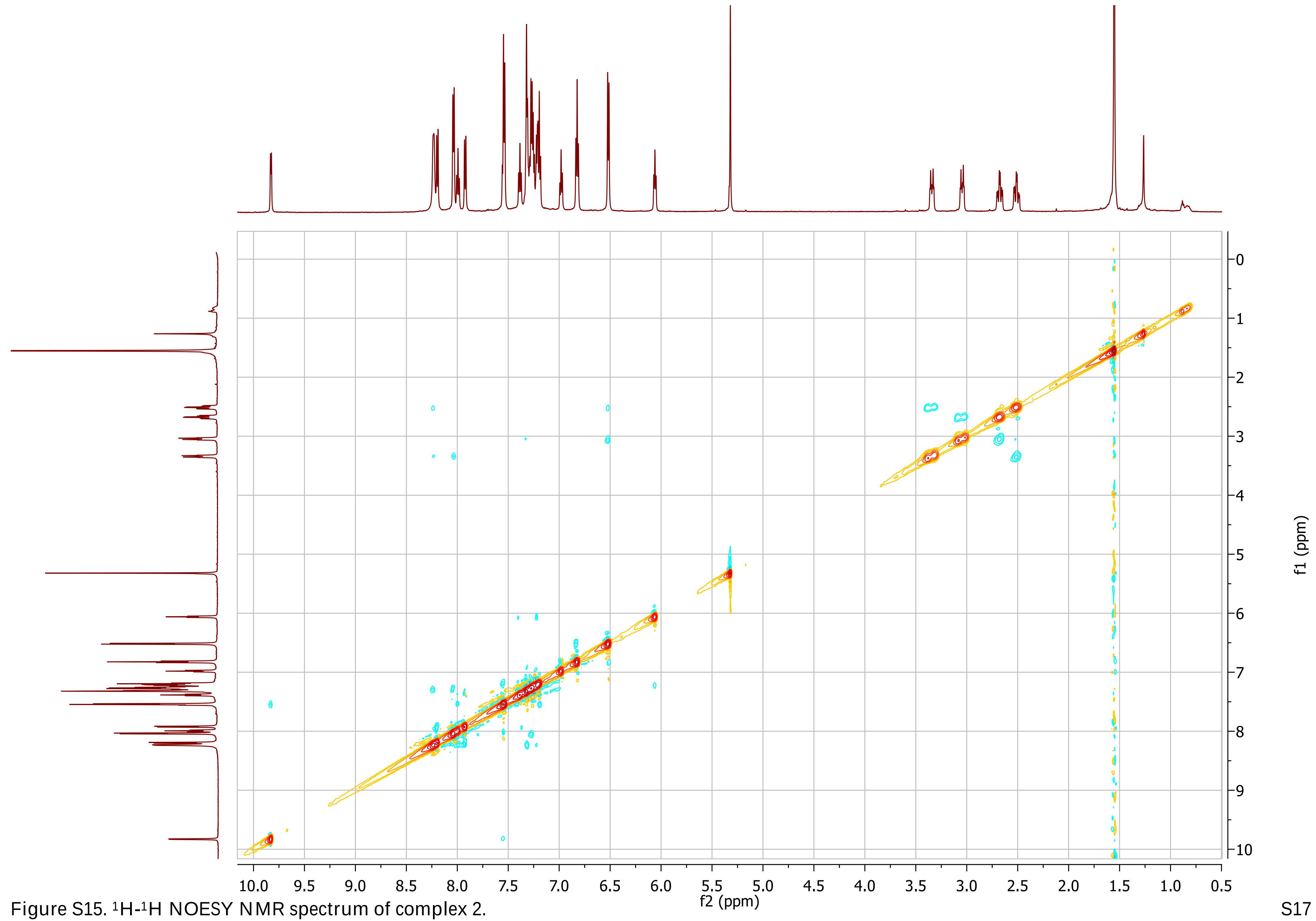

Figure S15. ¹H-¹H NOESY NMR spectrum of complex 2.

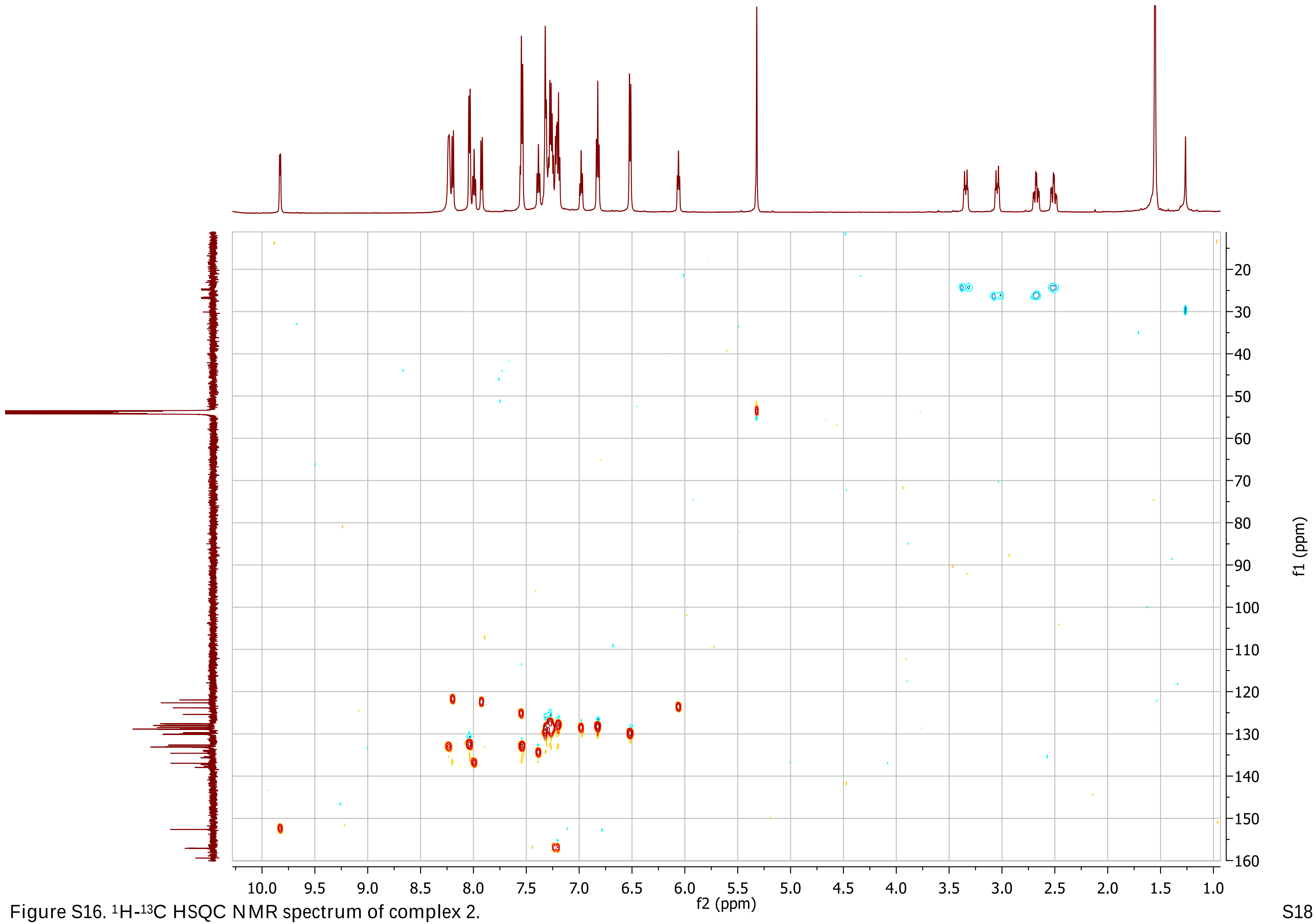

Figure S16. ^{1}H-^{13}C HSQC NMR spectrum of complex 2.

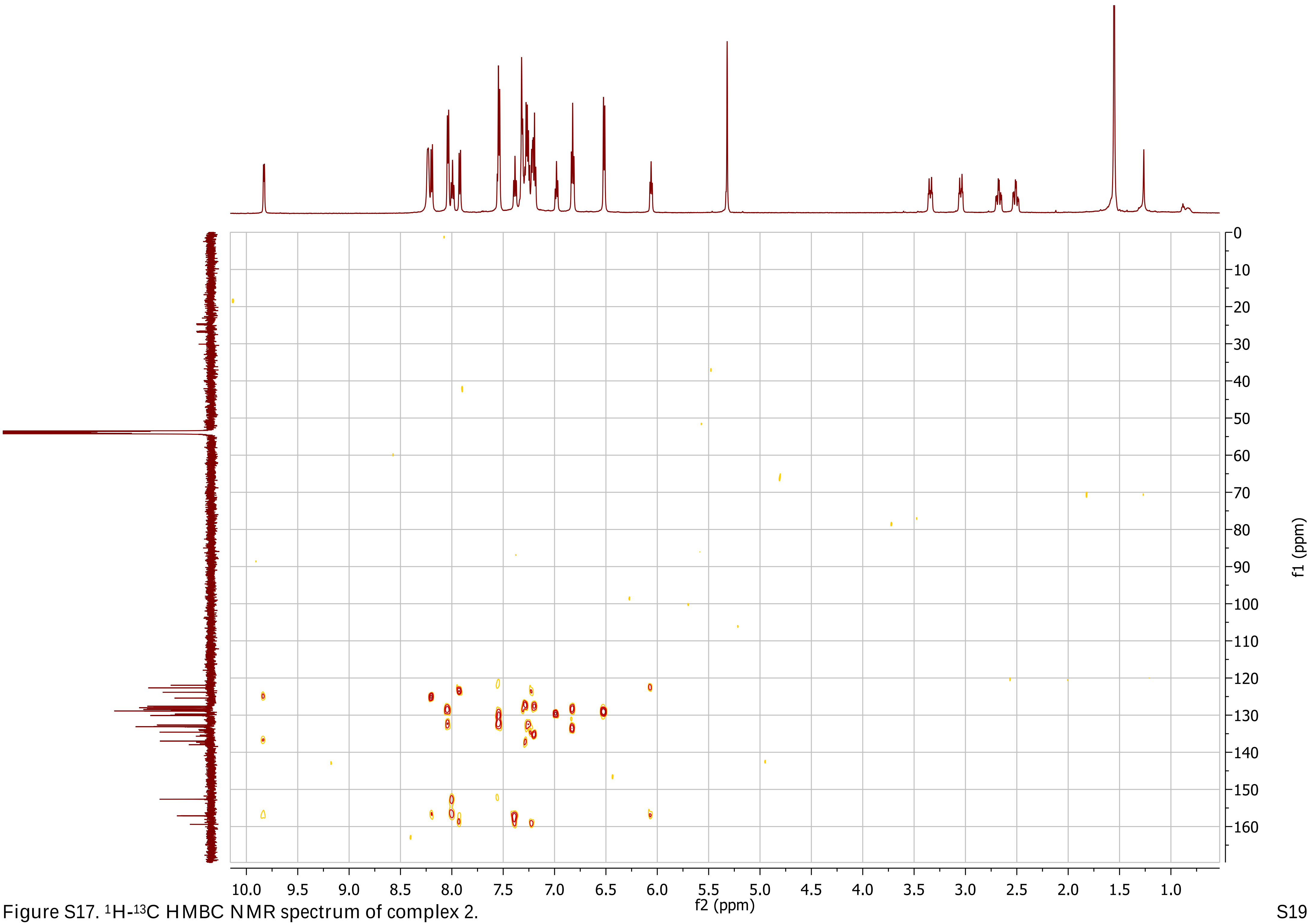

Figure S17. ^{1}H-^{13}C HMBC NMR spectrum of complex 2.

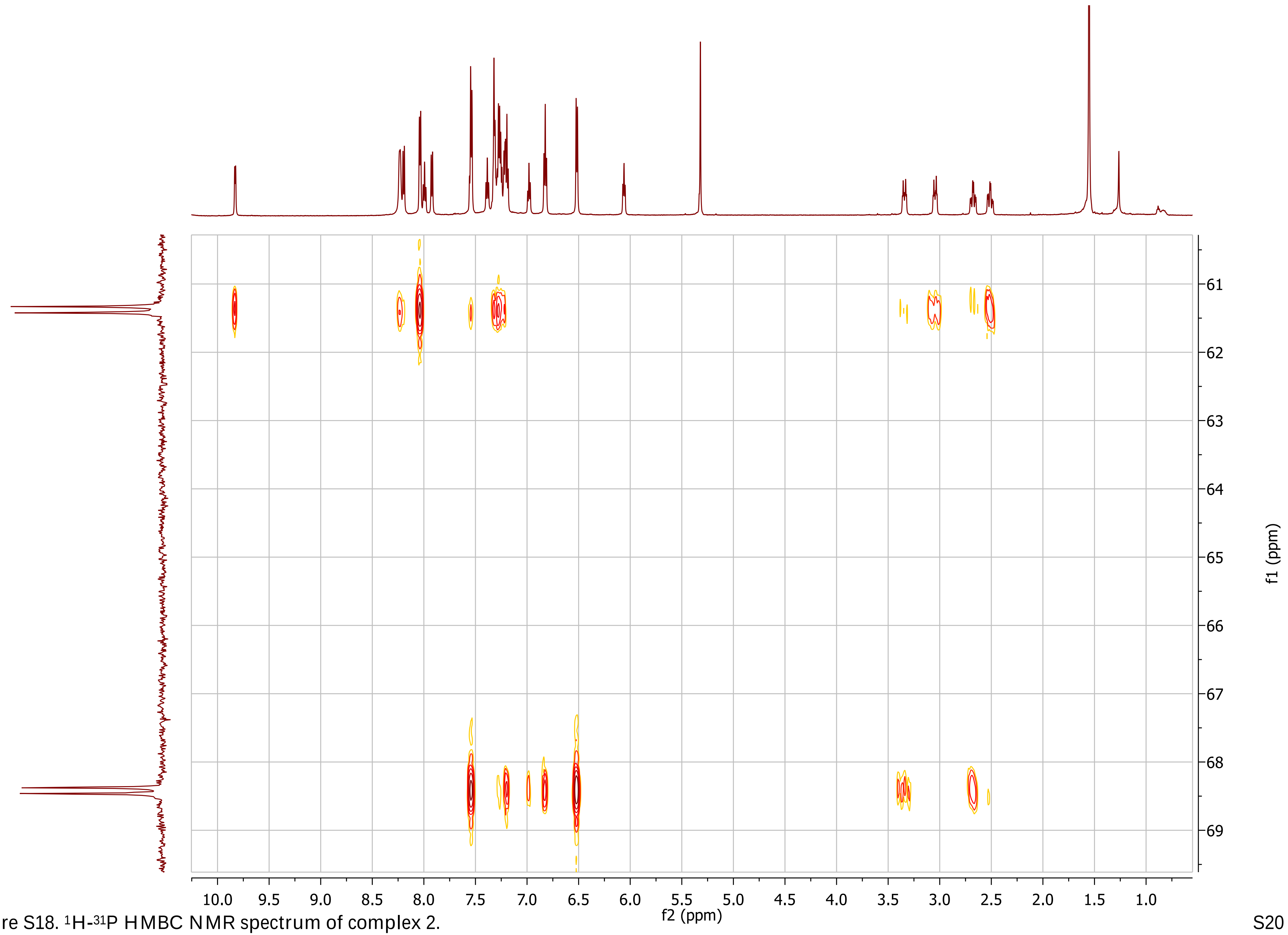

Figure S18. ^{1}H-^{31}P HMBC NMR spectrum of complex 2.

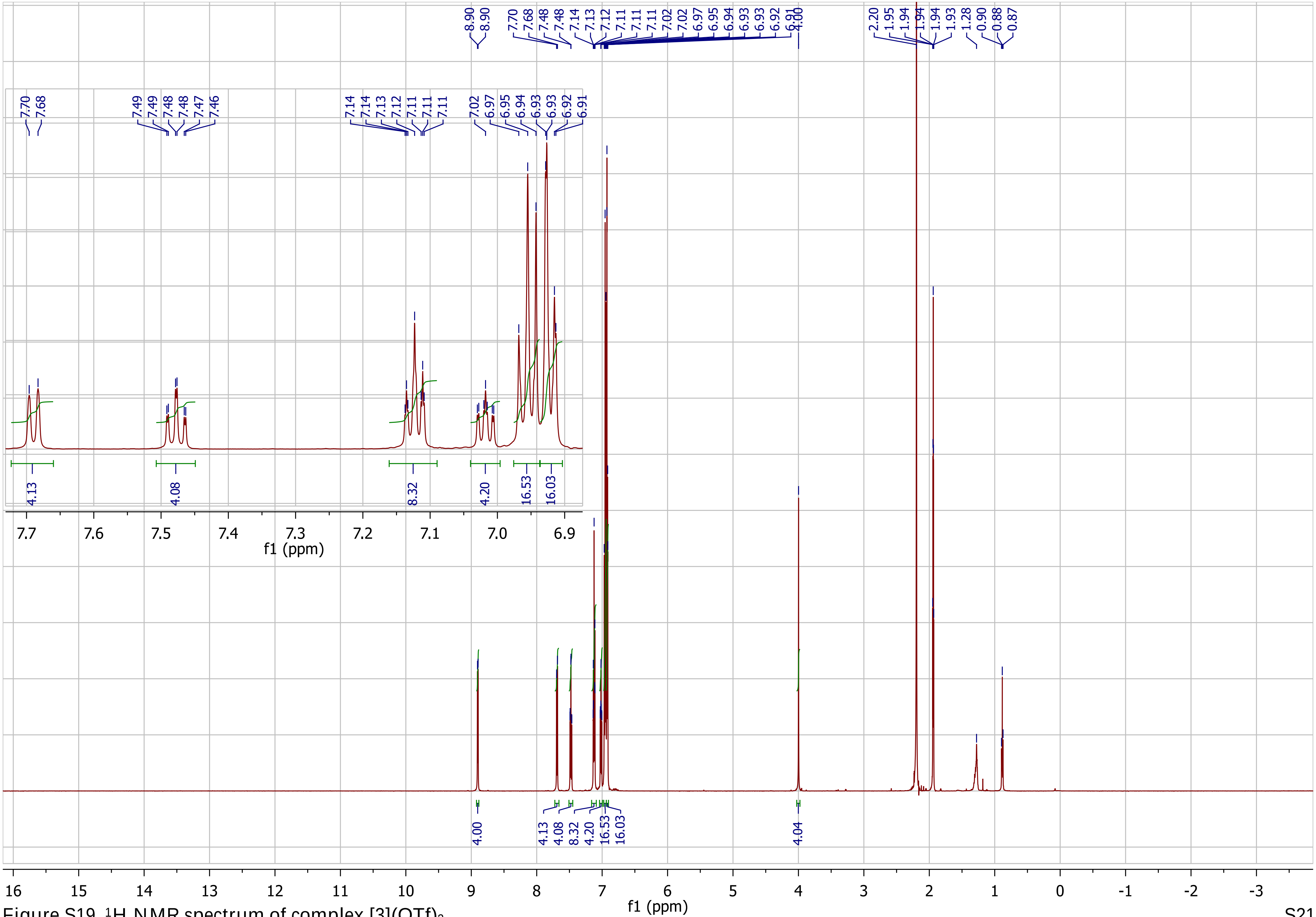

Figure S19. ^{1}H NMR spectrum of complex [3](OTf)$_2$.

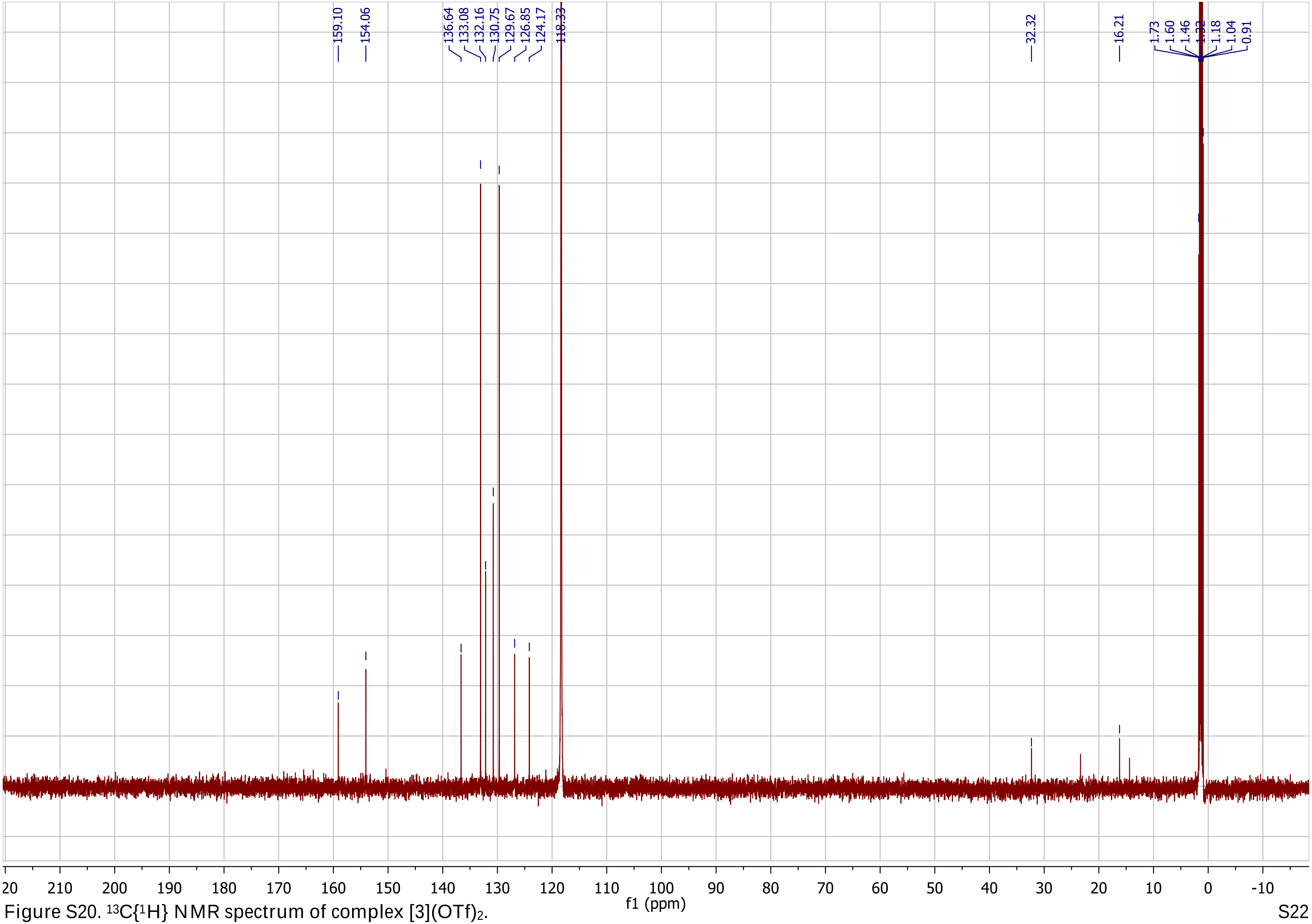

Figure S20. $^{13}C\{^1H\}$ NMR spectrum of complex [3](OTf)$_2$.

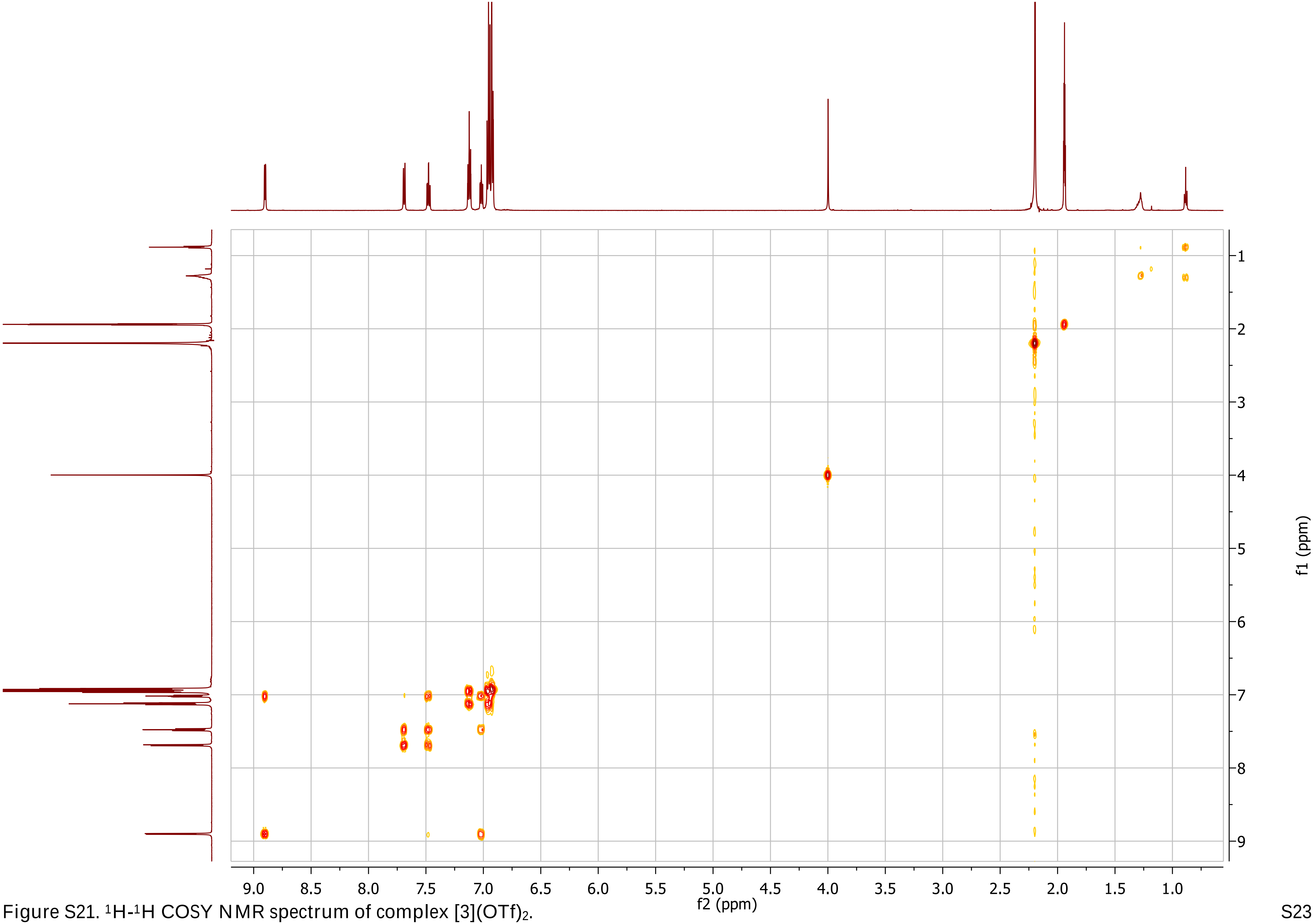

Figure S21. ^{1}H-^{1}H COSY NMR spectrum of complex [3](OTf)$_2$.

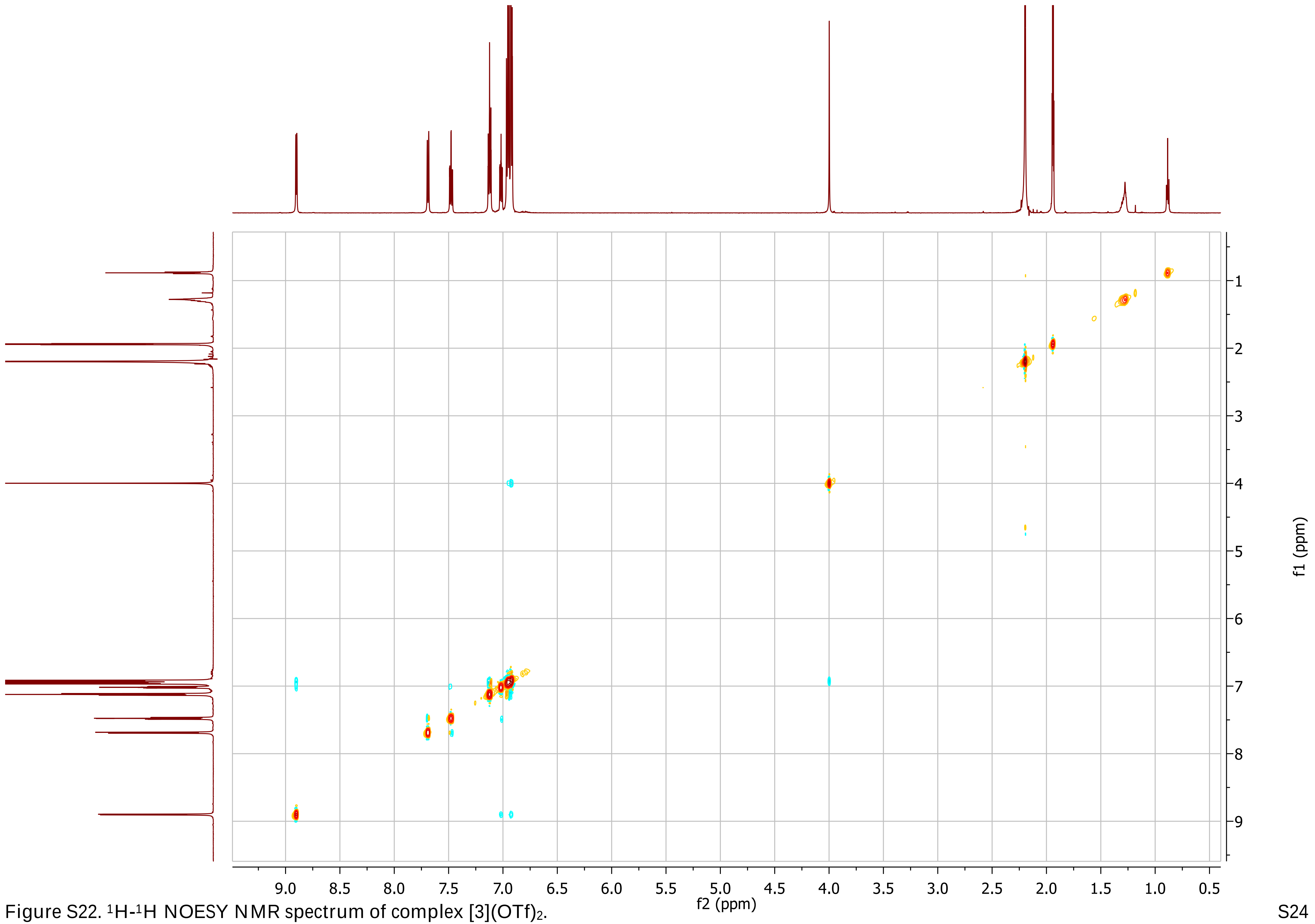

Figure S22. ^1H-^1H NOESY NMR spectrum of complex [3](OTf)$_2$.

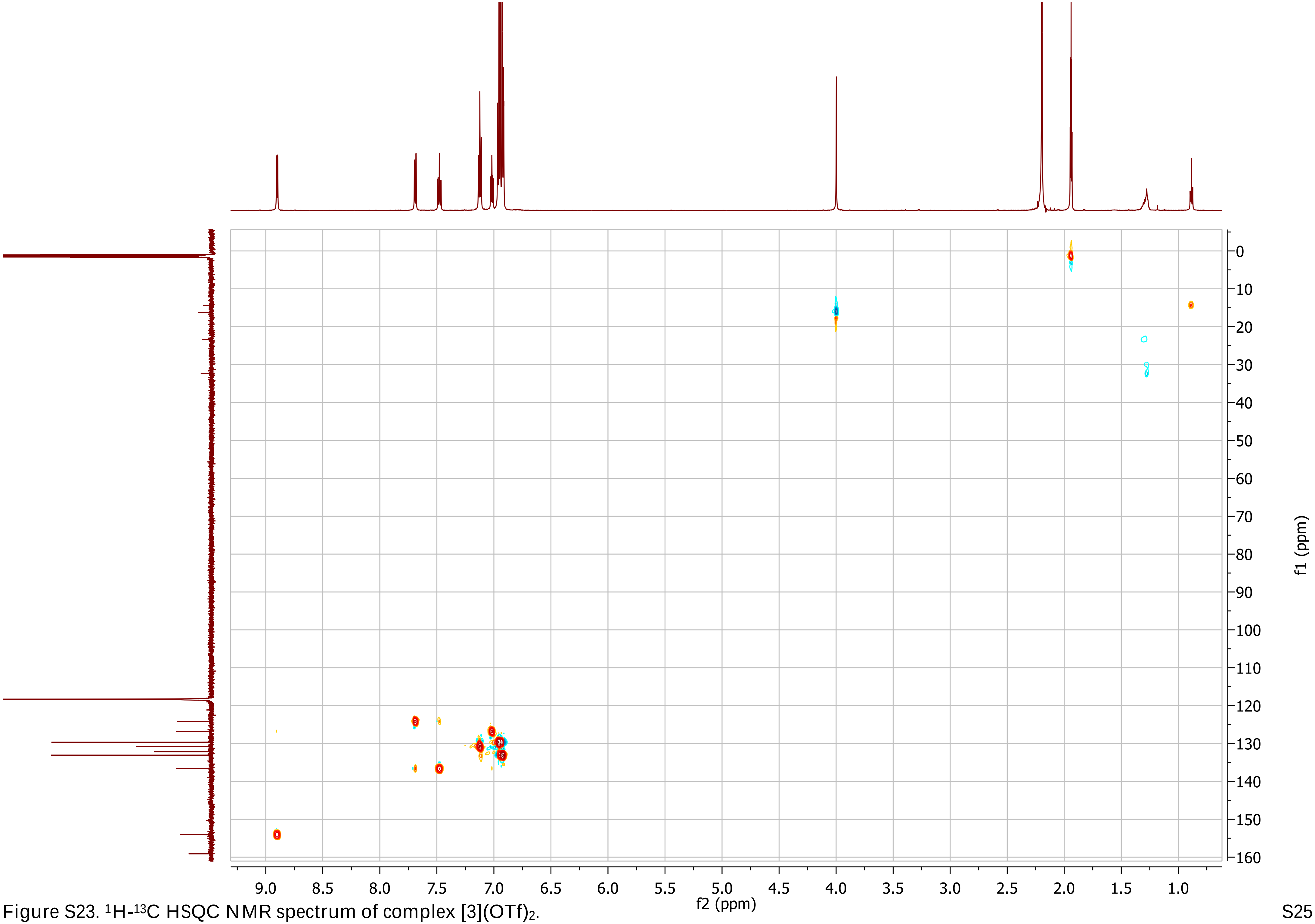

Figure S23. ^{1}H-^{13}C HSQC NMR spectrum of complex [3](OTf)$_2$.

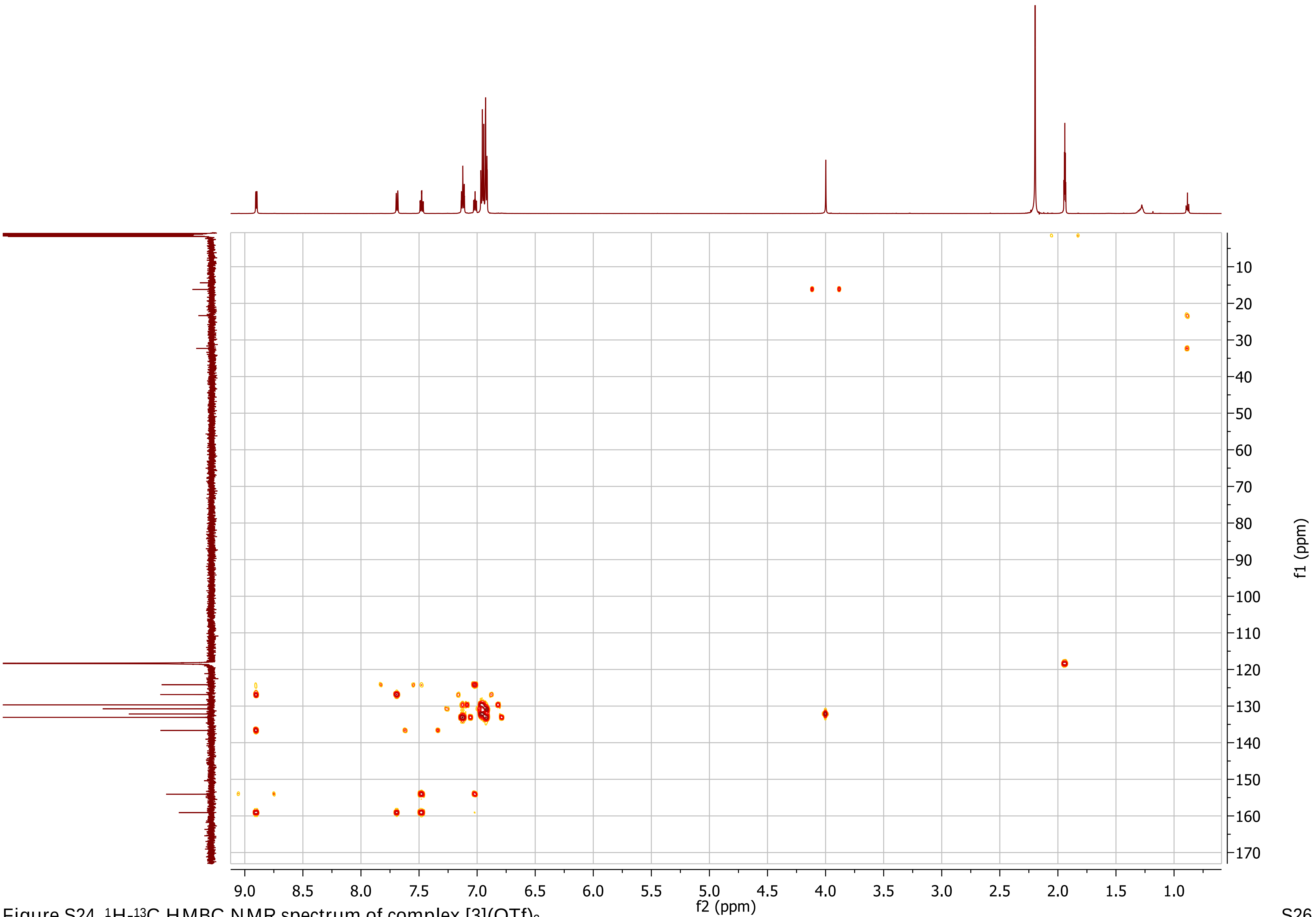

Figure S24. ^{1}H-^{13}C HMBC NMR spectrum of complex [3](OTf)$_2$.

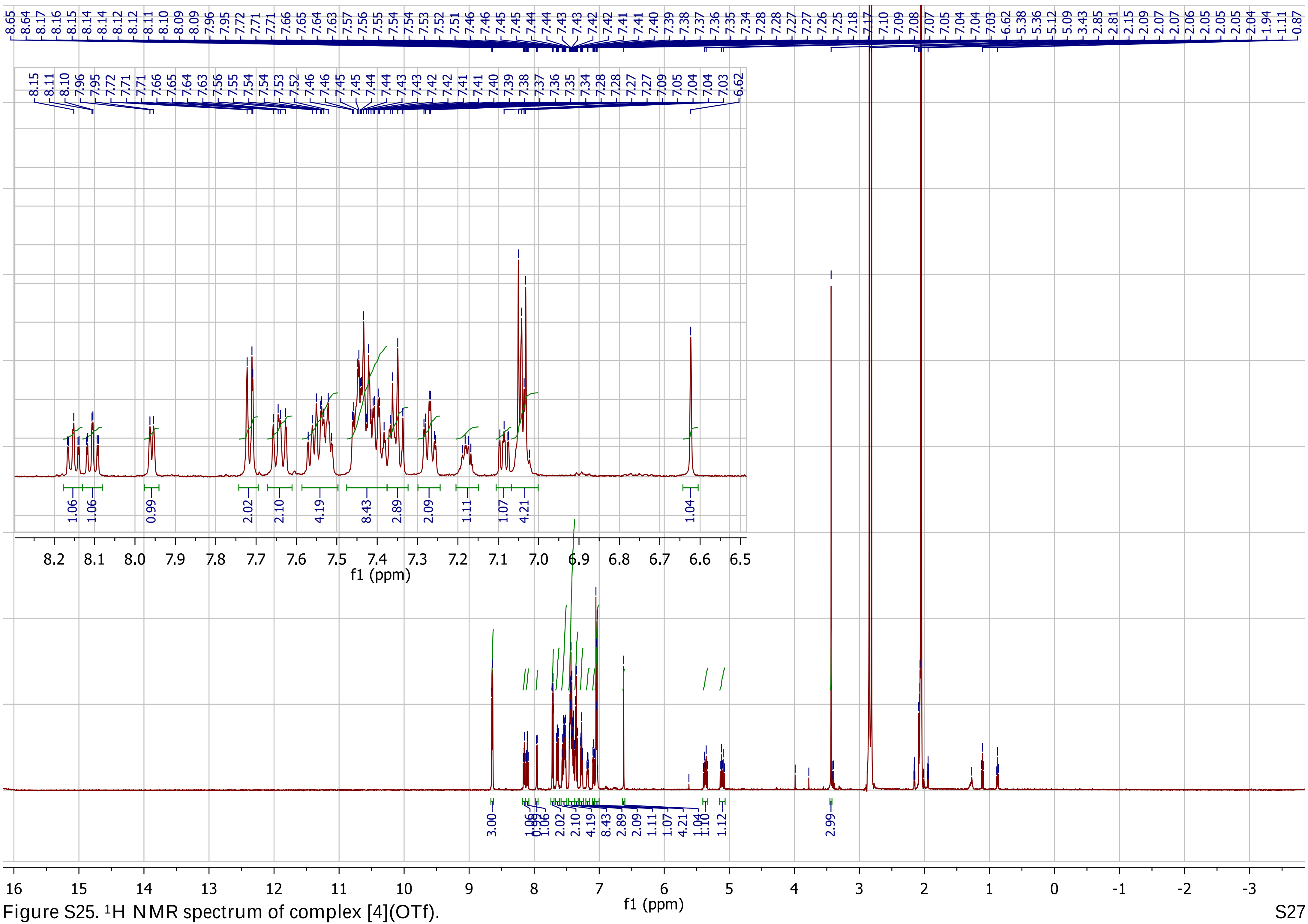

Figure S25. ^{1}H NMR spectrum of complex [4](OTf).

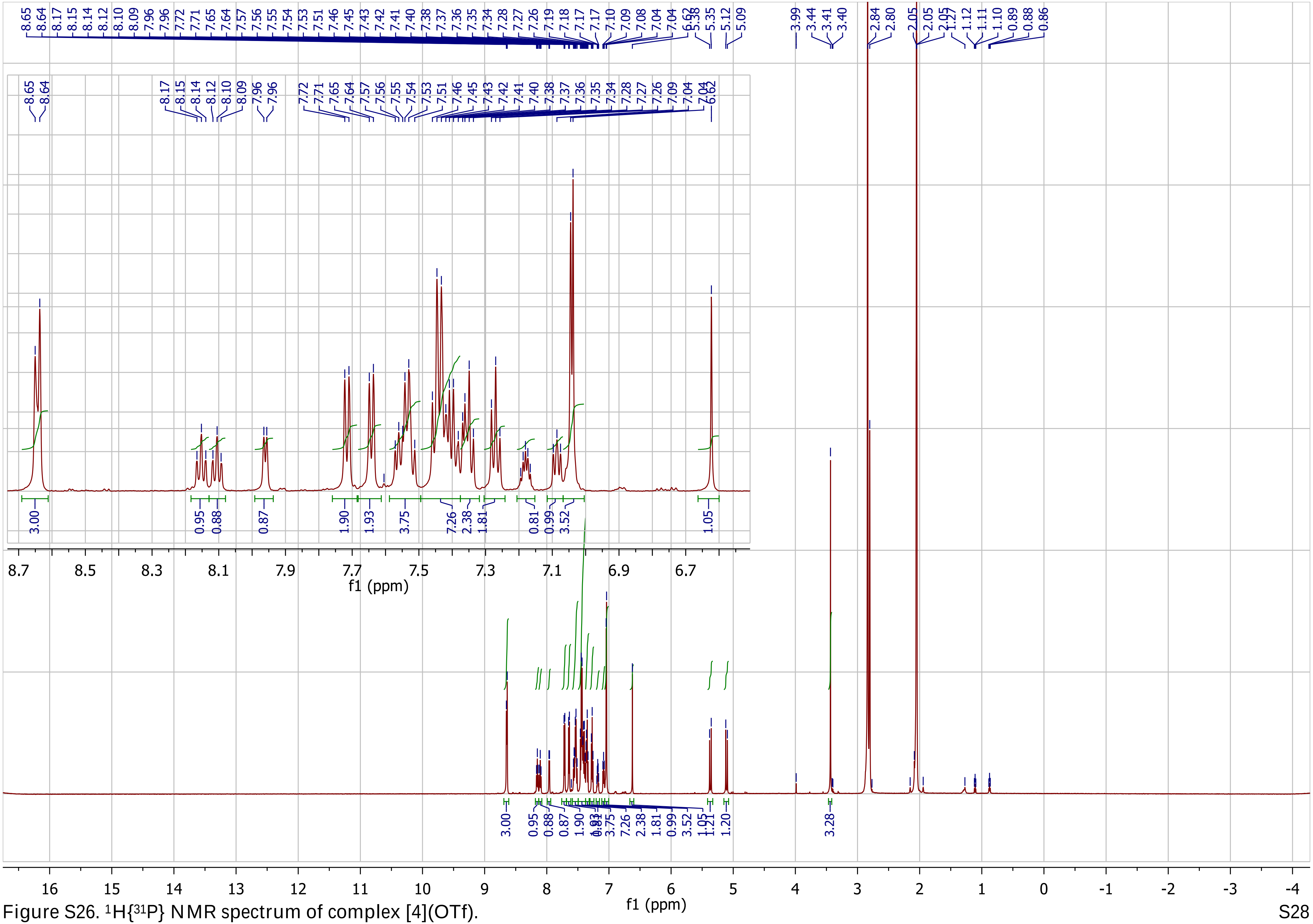

Figure S26. $^1H\{^{31}P\}$ NMR spectrum of complex [4](OTf).

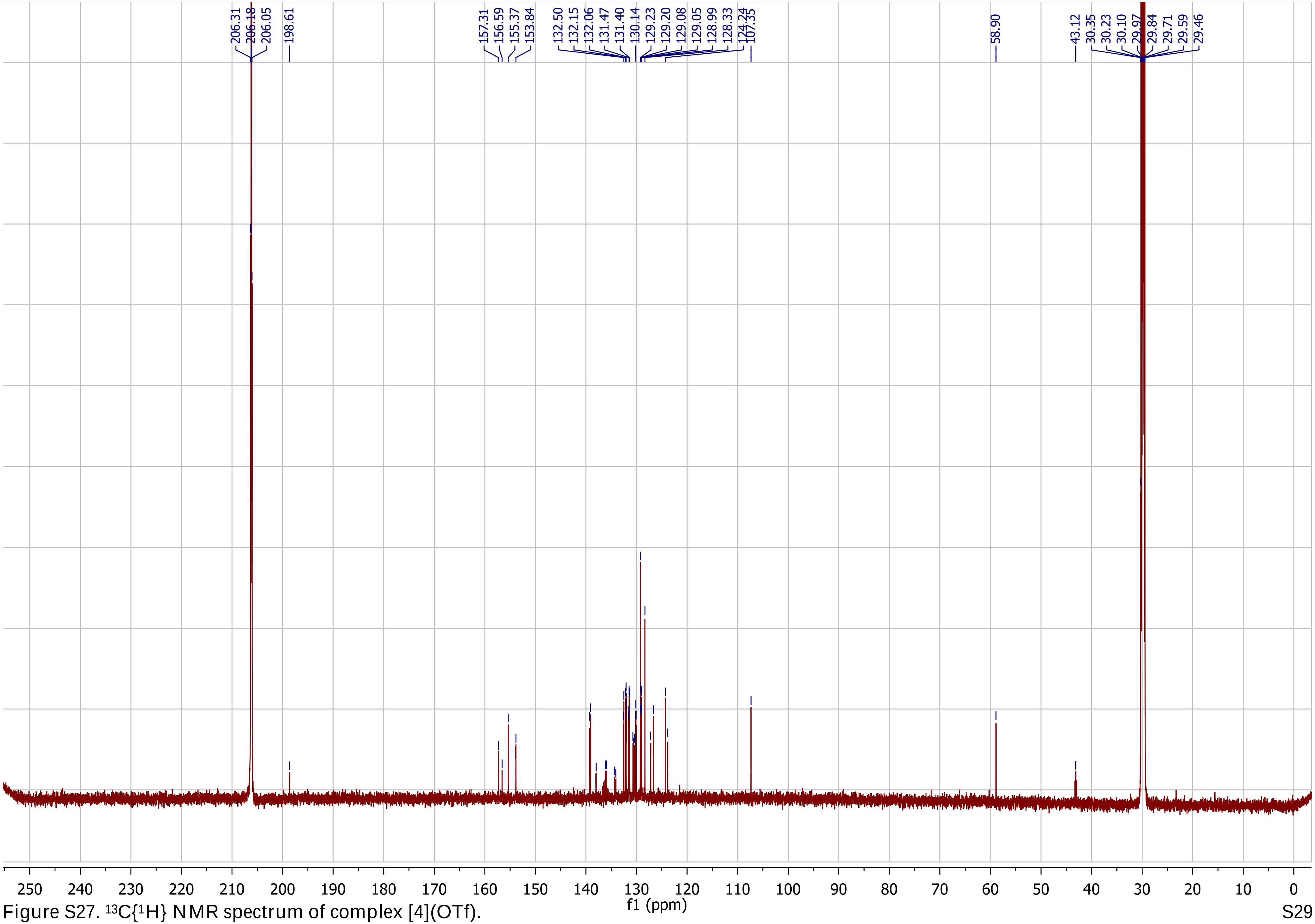

Figure S27. $^{13}C\{^{1}H\}$ NMR spectrum of complex [4](OTf).

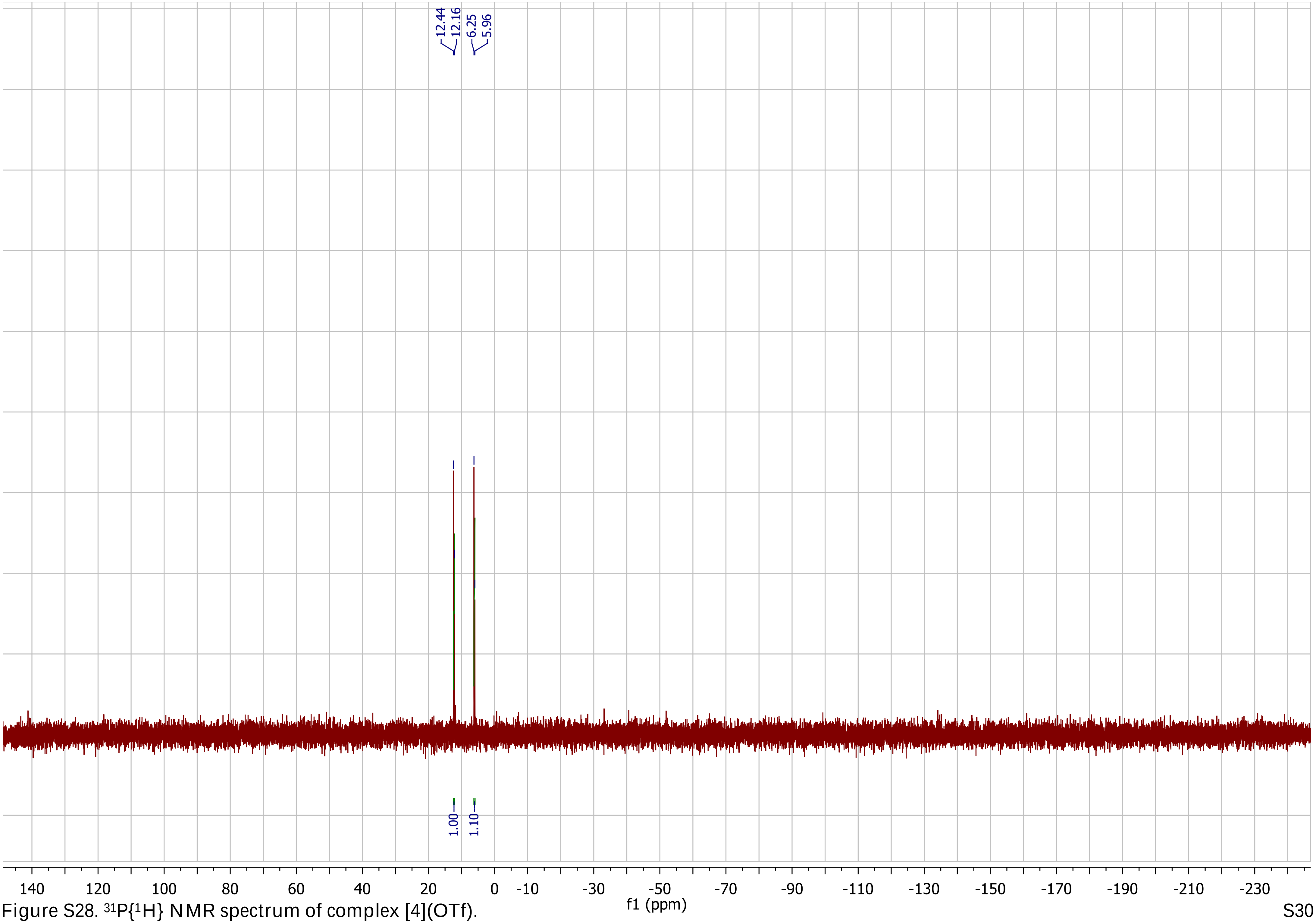

Figure S28. $^{31}P\{^{1}H\}$ NMR spectrum of complex [4](OTf).

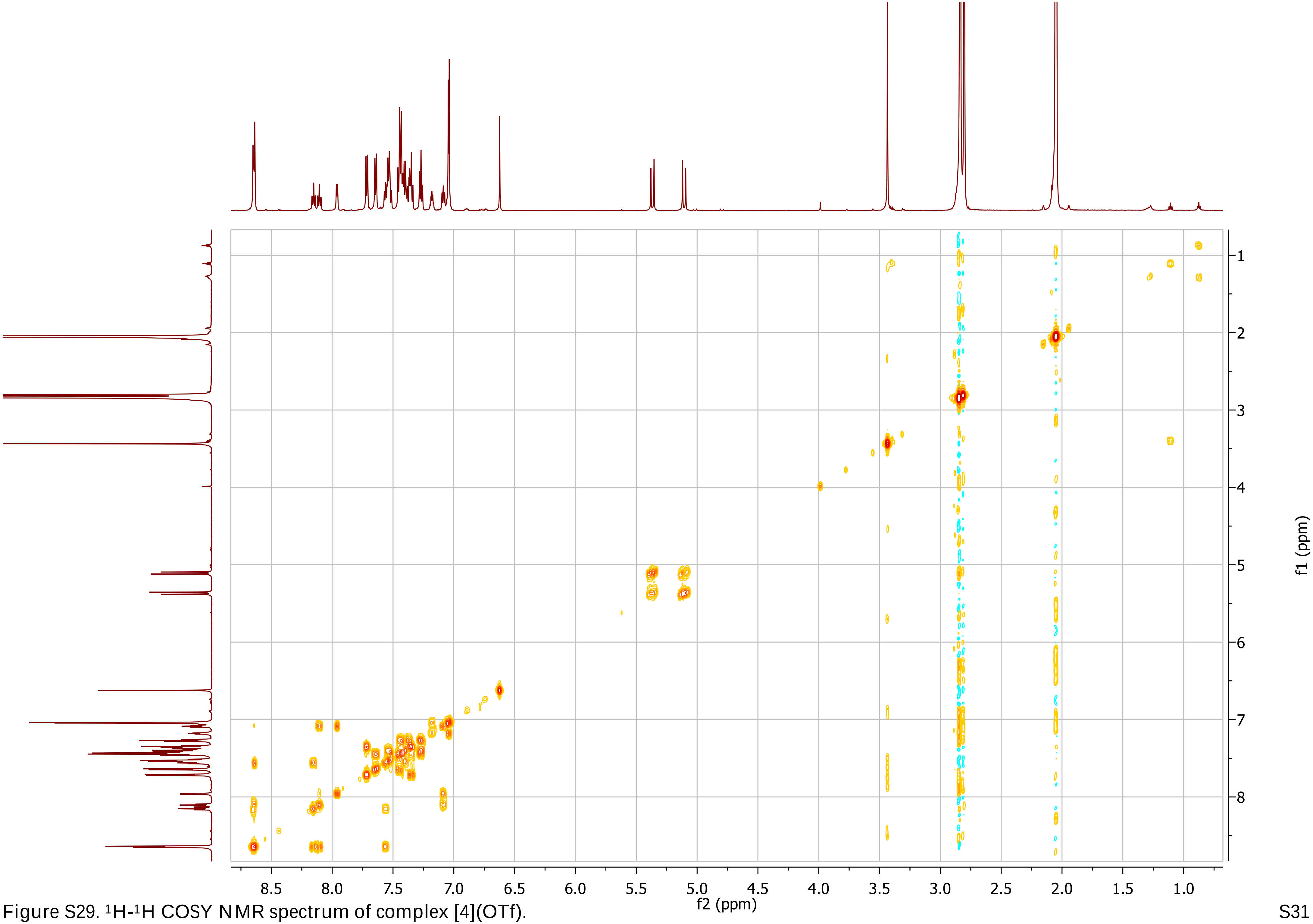

Figure S29. ^{1}H-^{1}H COSY NMR spectrum of complex [4](OTf).

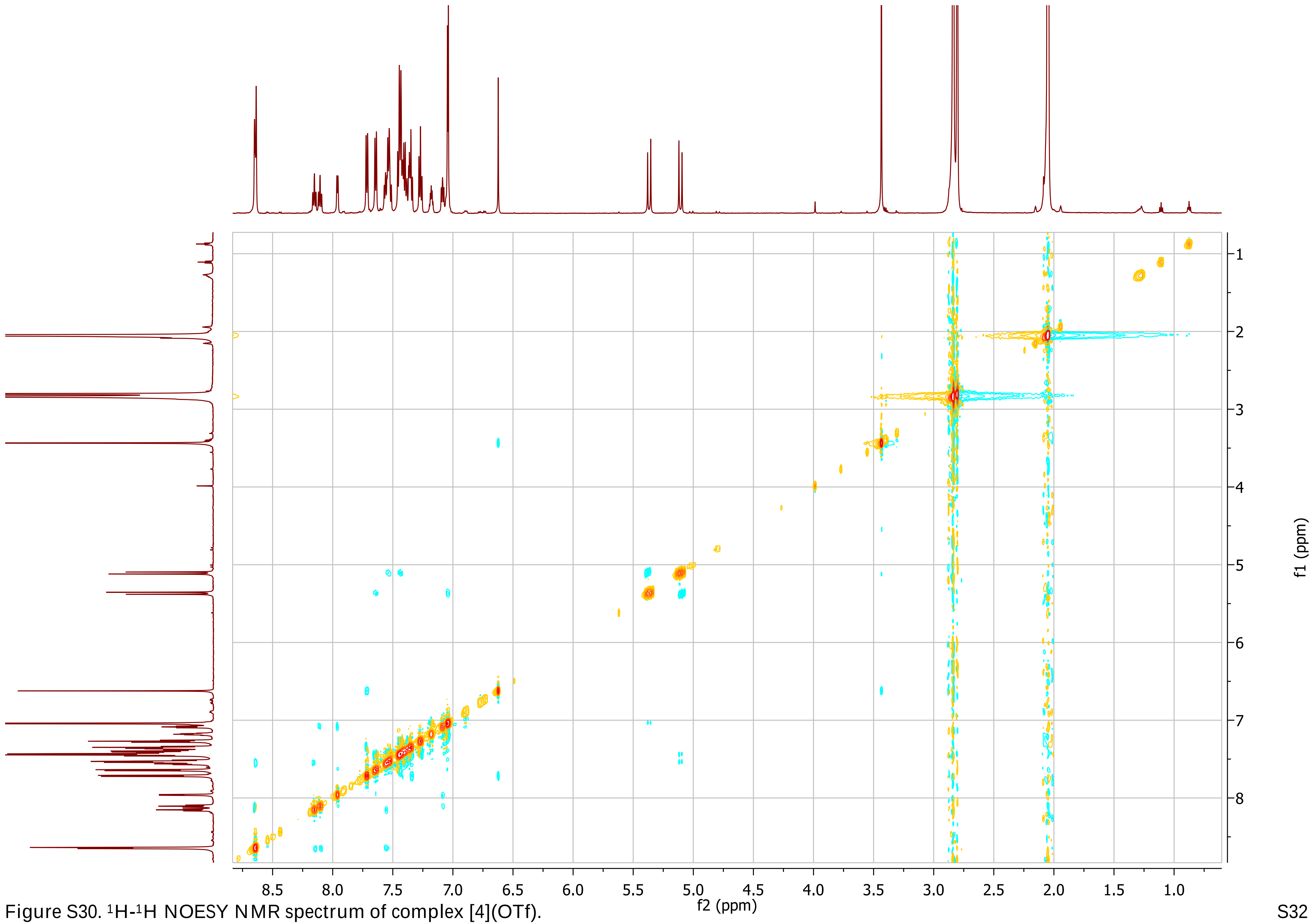

Figure S30. ^{1}H-^{1}H NOESY NMR spectrum of complex [4](OTf).

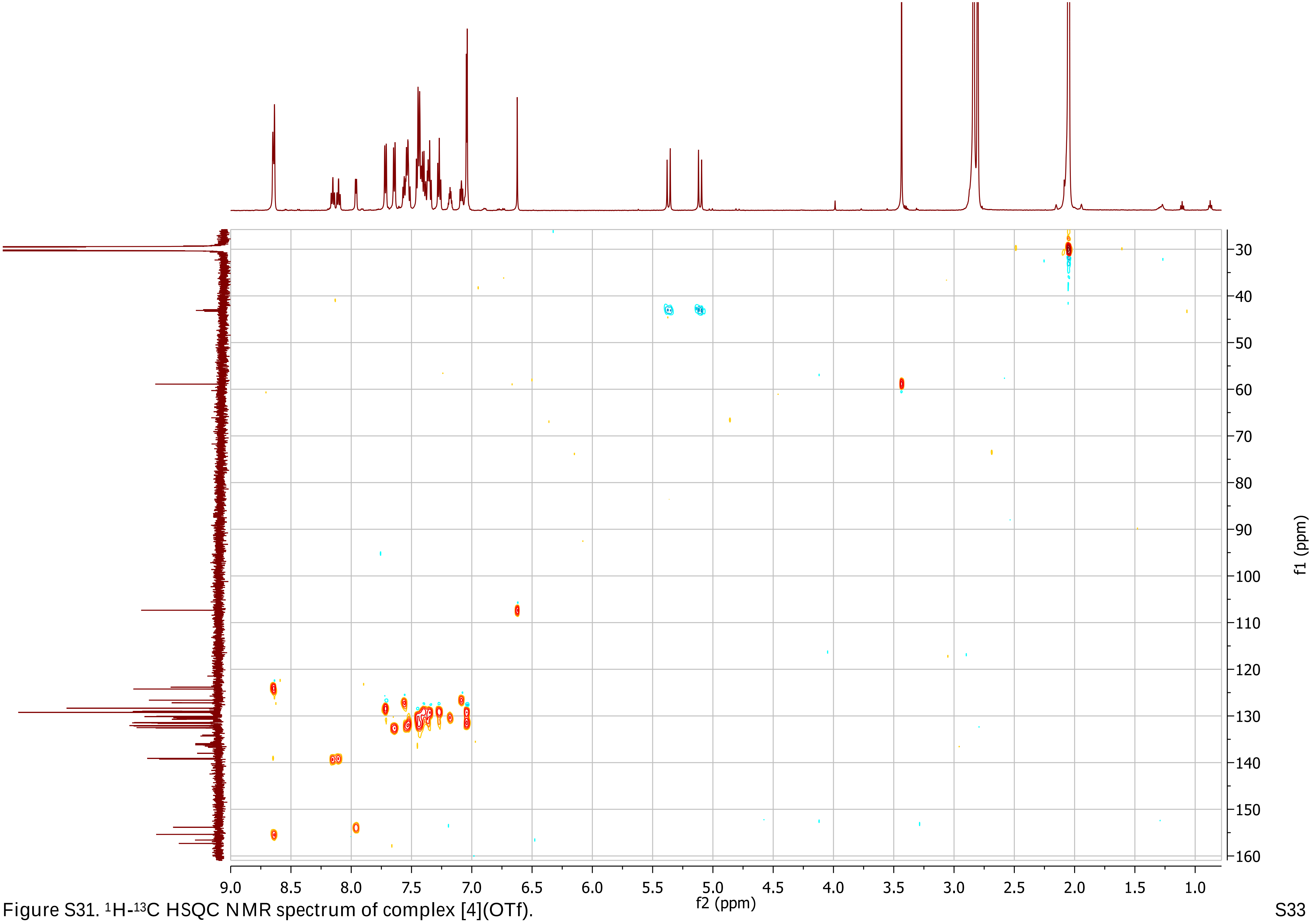

Figure S31. ^{1}H-^{13}C HSQC NMR spectrum of complex [4](OTf).

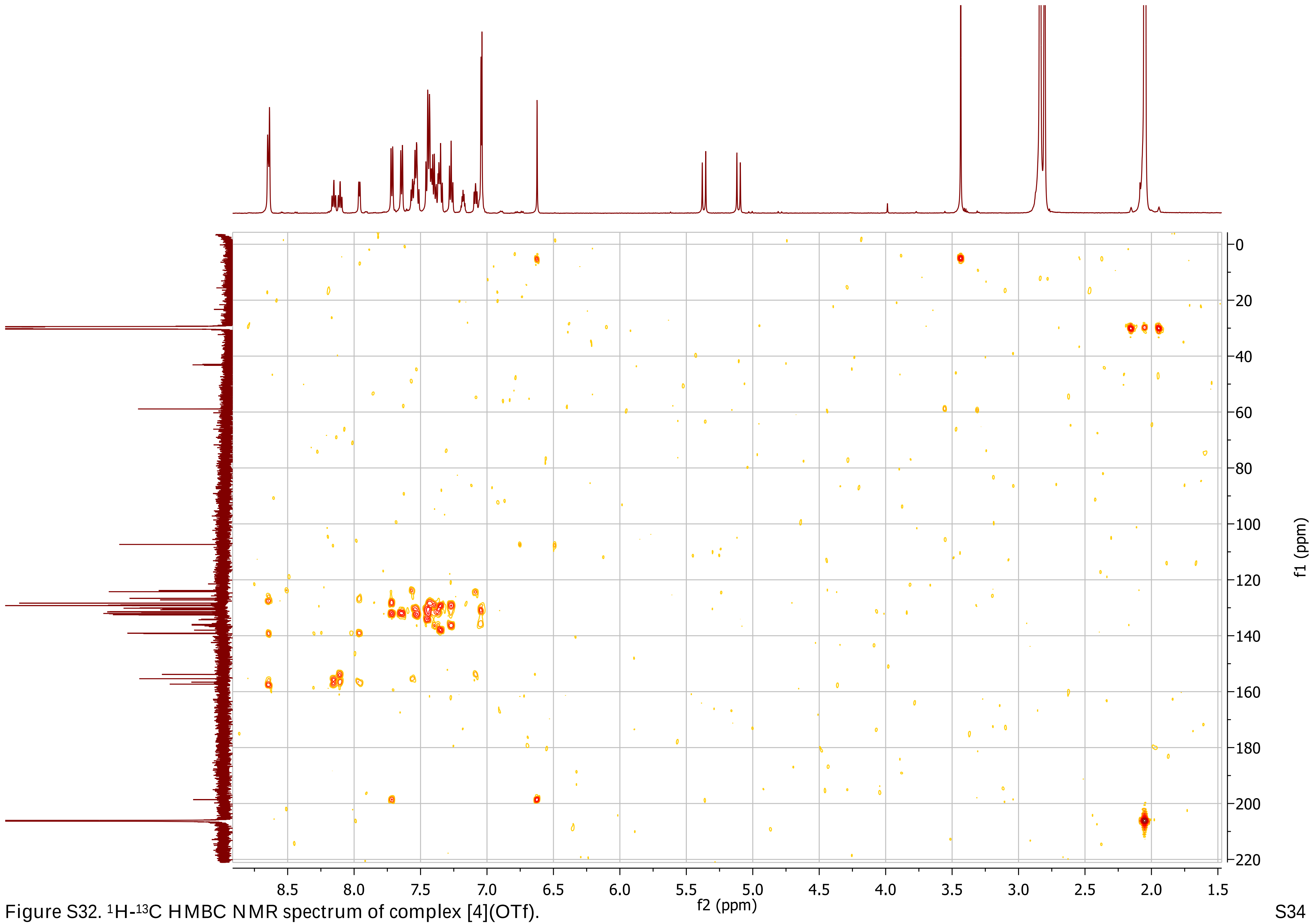

Figure S32. ^{1}H-^{13}C HMBC NMR spectrum of complex [4](OTf).

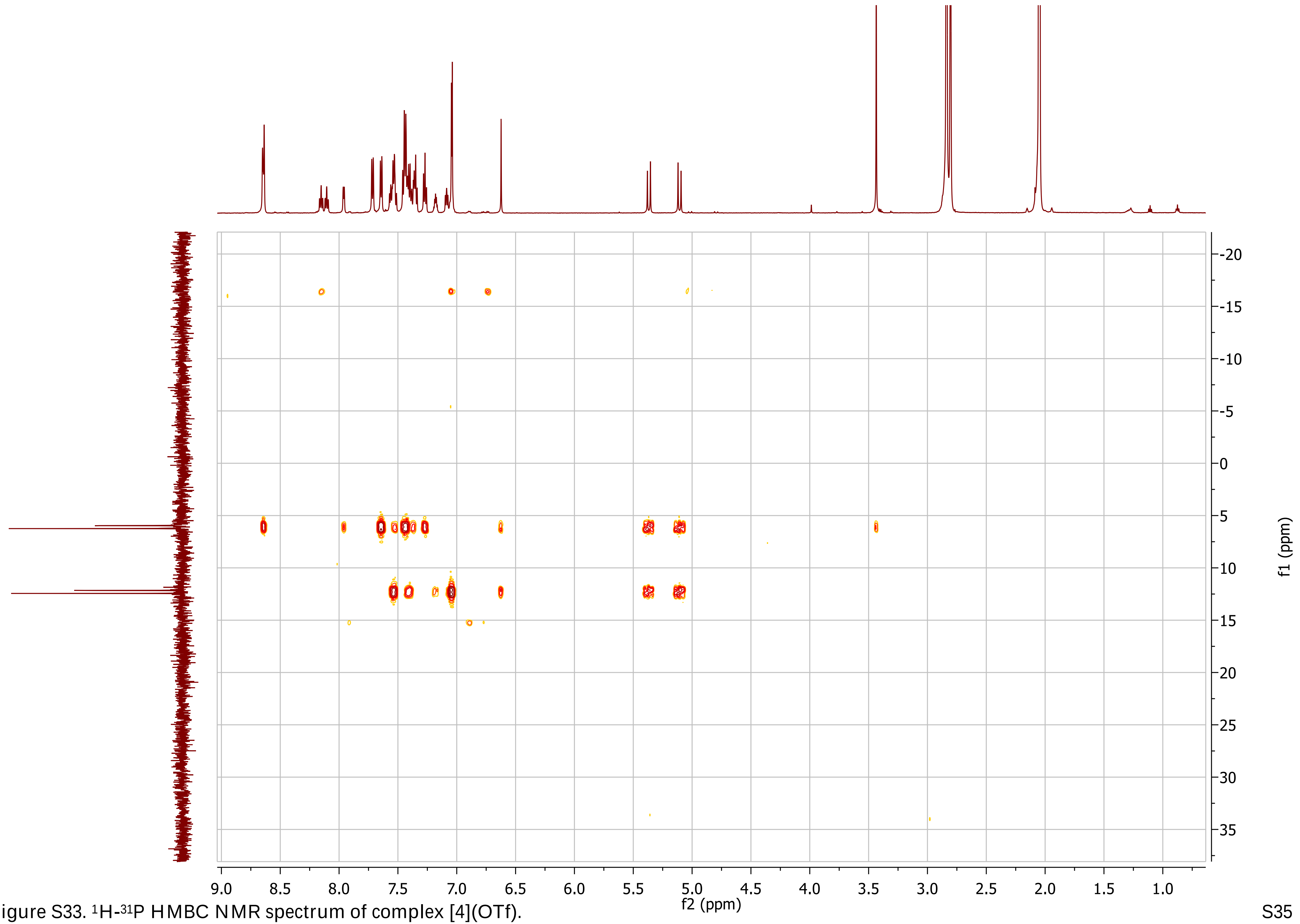

Figure S33. ^{1}H-^{31}P HMBC NMR spectrum of complex [4](OTf).

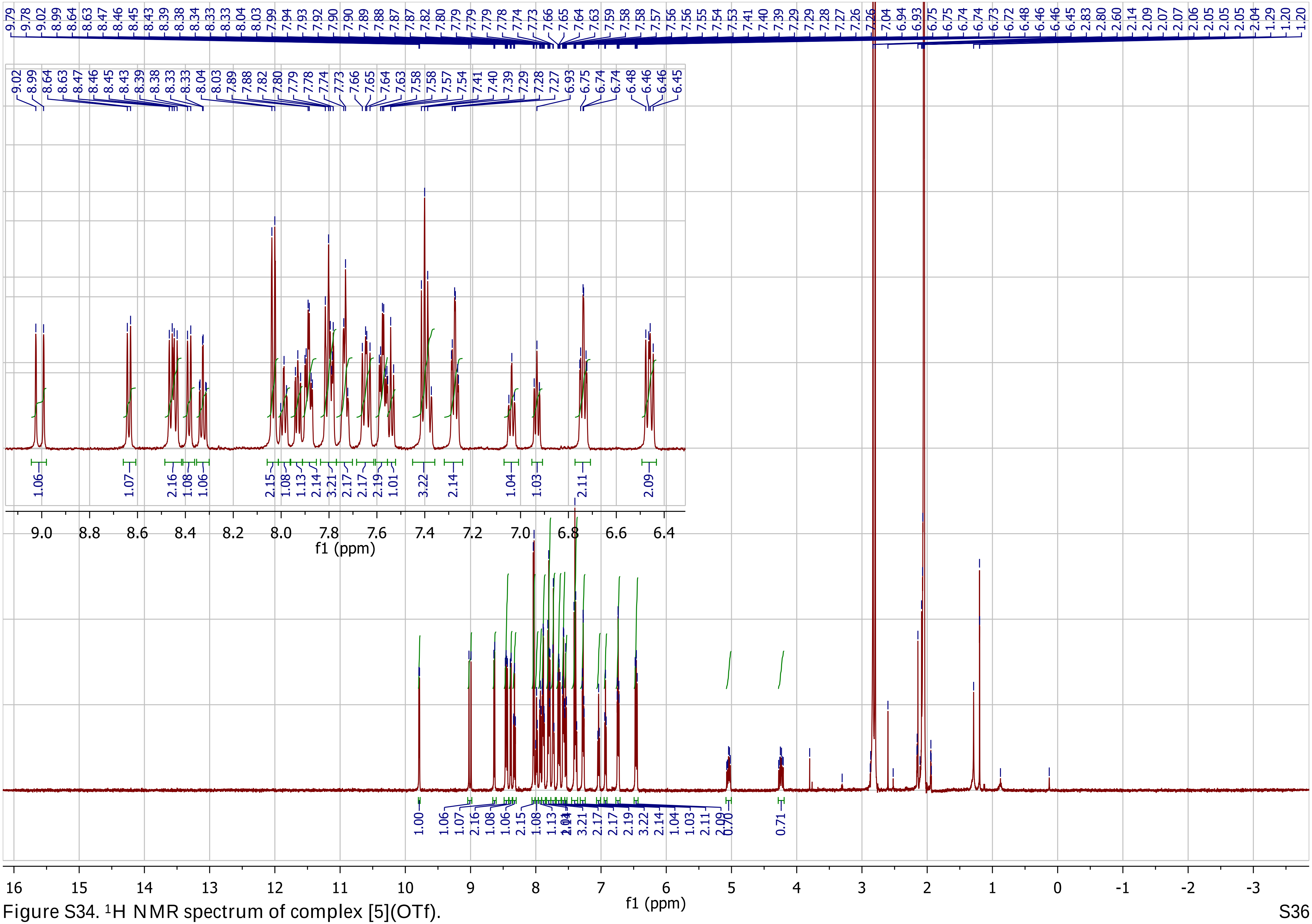

Figure S34. ¹H NMR spectrum of complex [5](OTf).

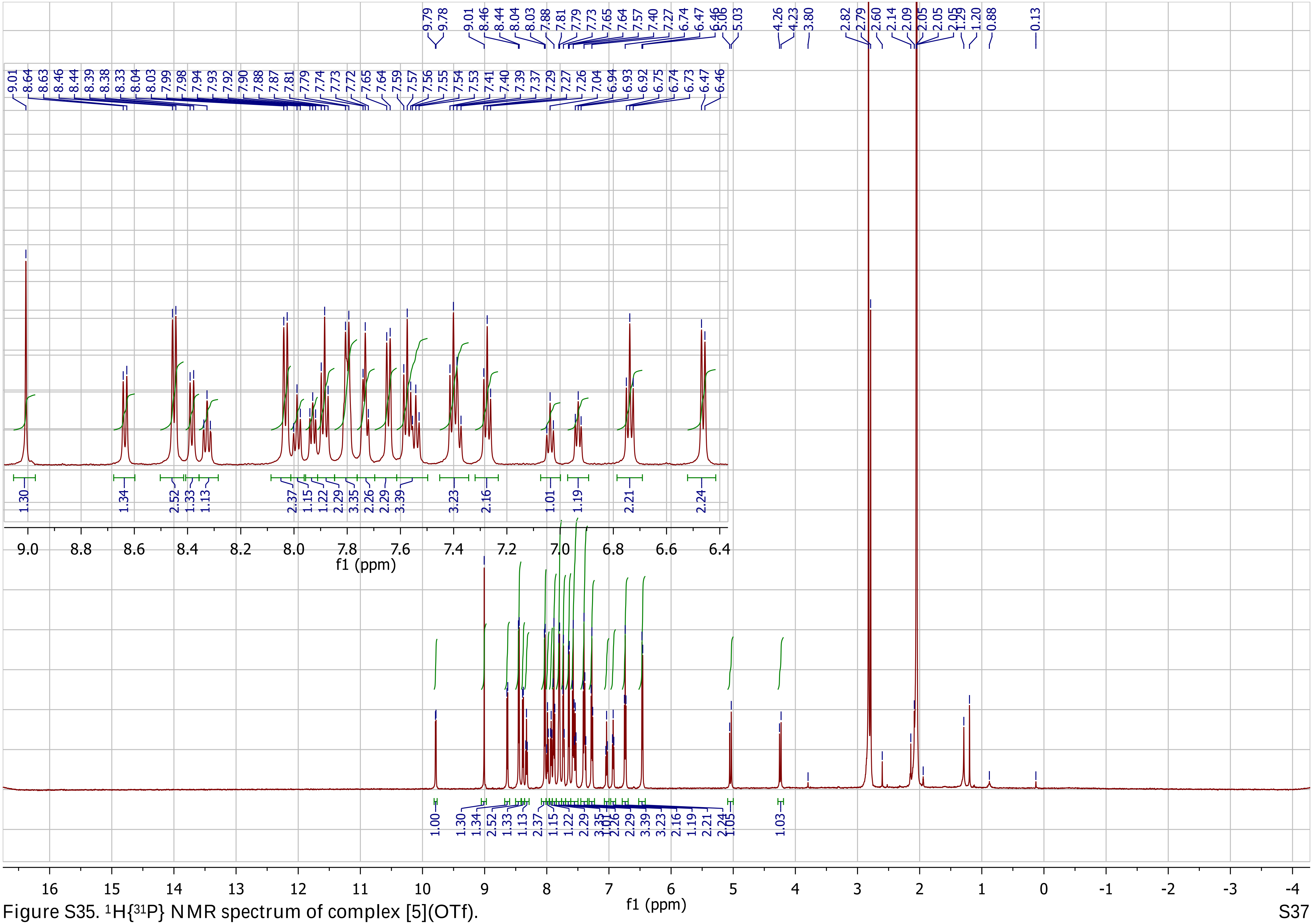

Figure S35. $^{1}H\{^{31}P\}$ NMR spectrum of complex [5](OTf).

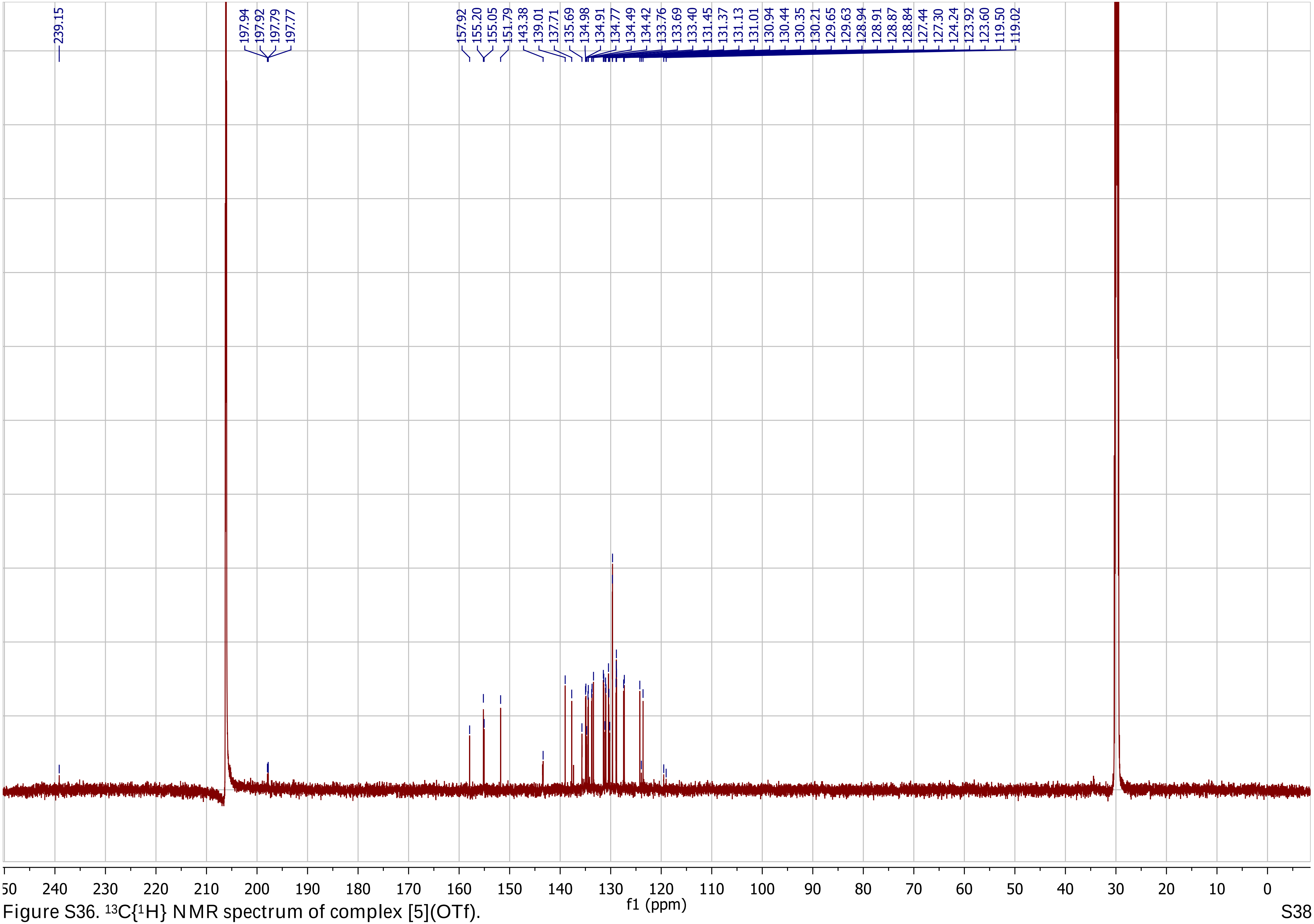

Figure S36. $^{13}C\{^{1}H\}$ NMR spectrum of complex [5](OTf).

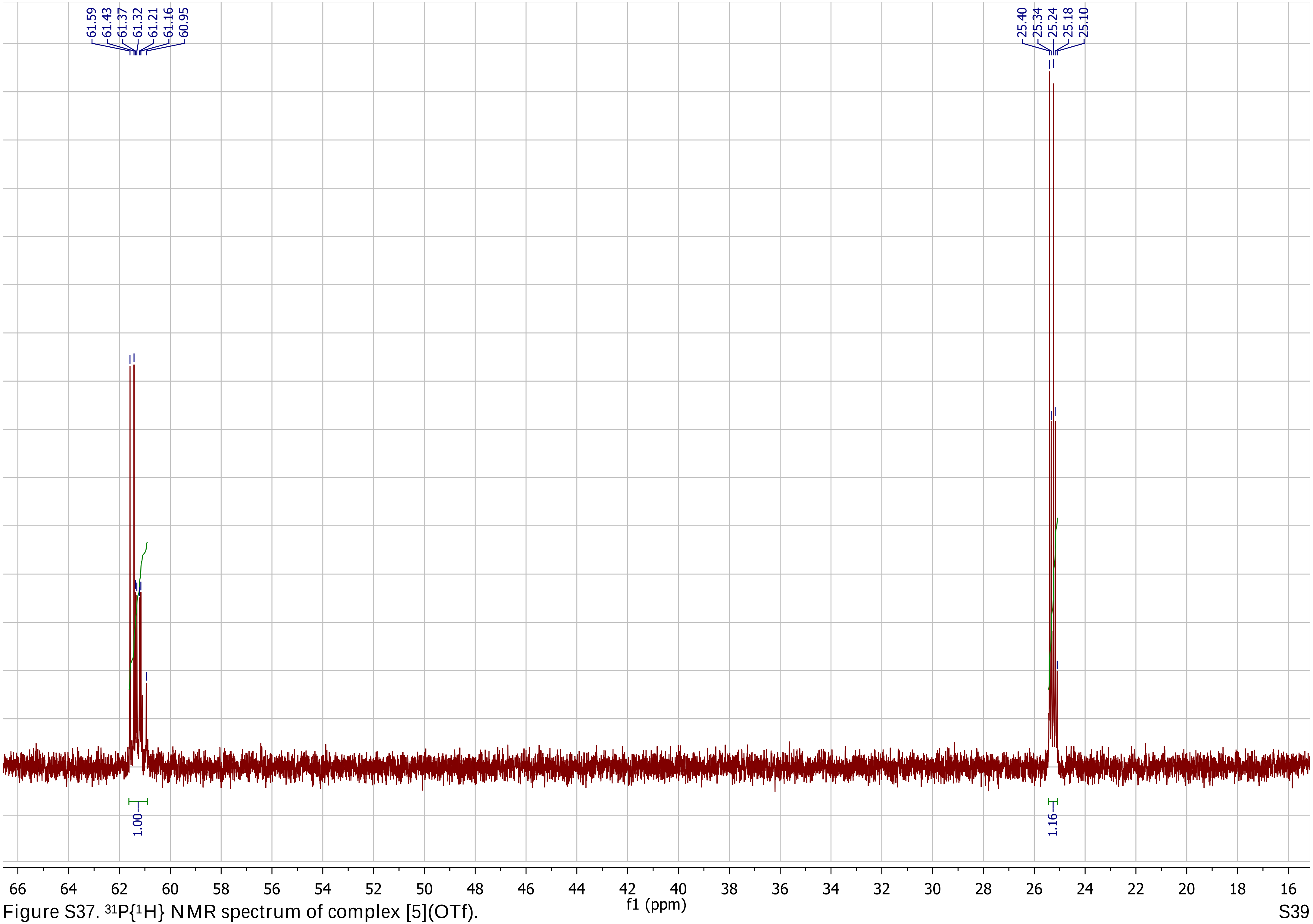

Figure S37. $^{31}P\{^{1}H\}$ NMR spectrum of complex [5](OTf).

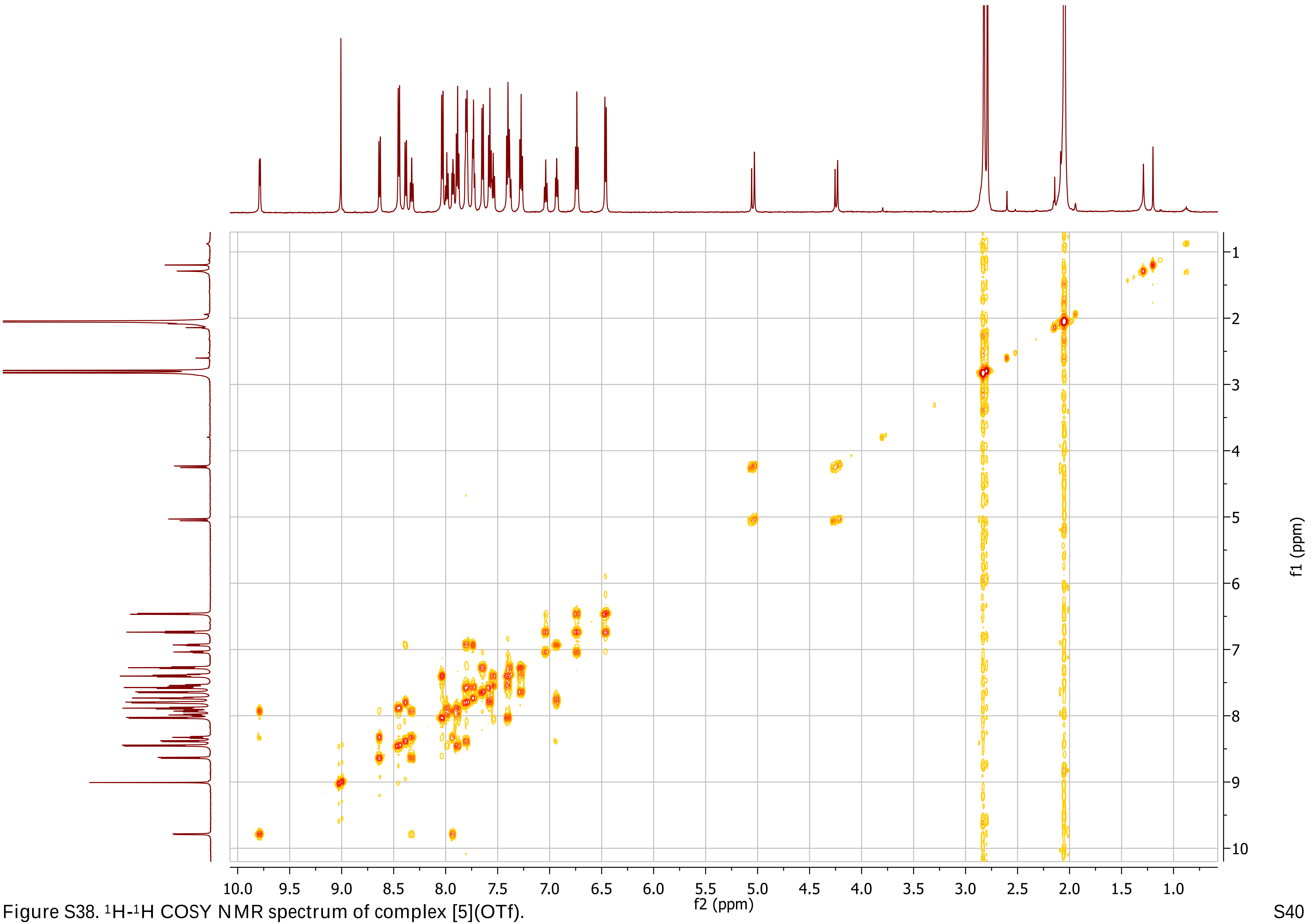

Figure S38. ^{1}H-^{1}H COSY NMR spectrum of complex [5](OTf).

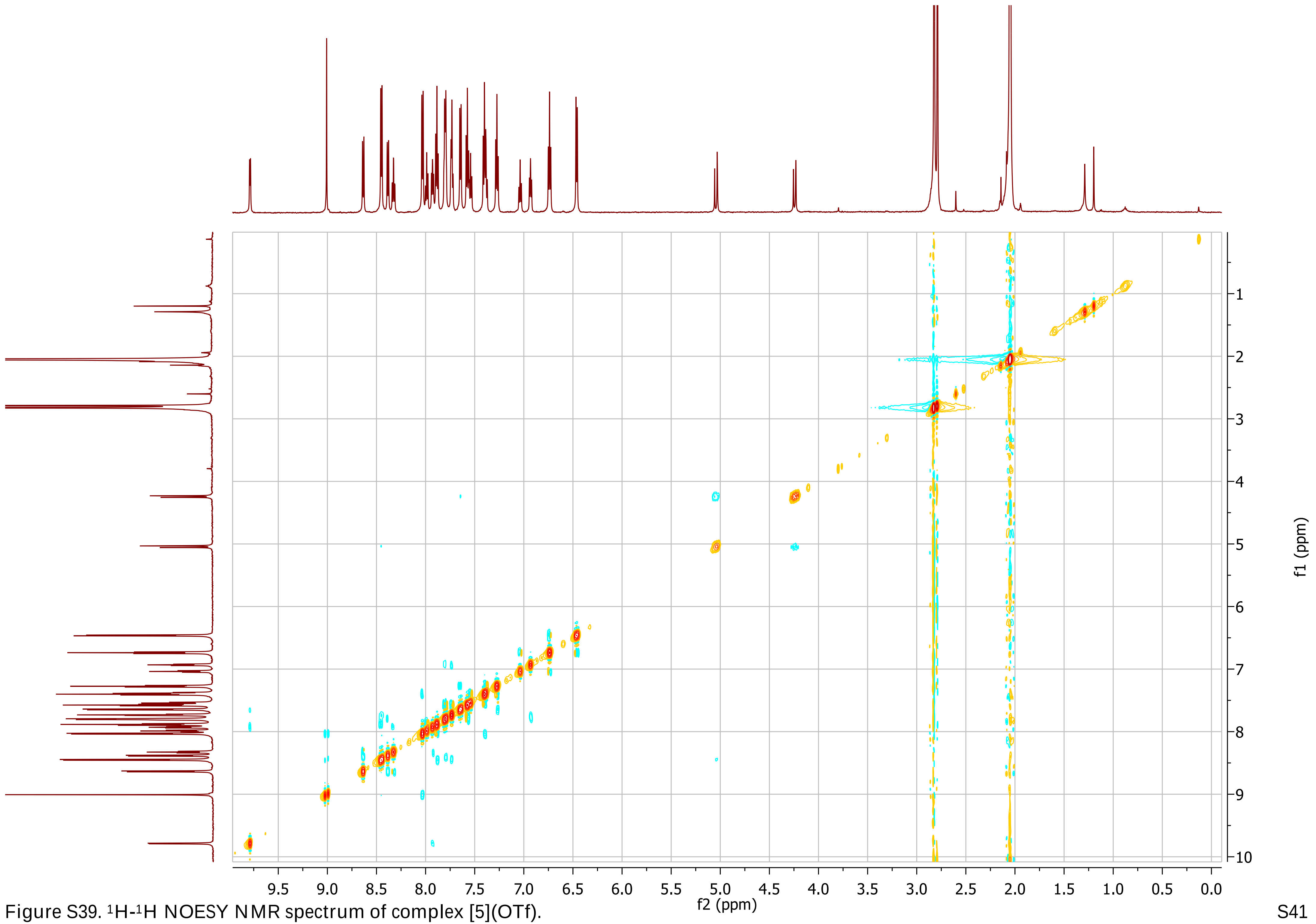

Figure S39. ^{1}H-^{1}H NOESY NMR spectrum of complex [5](OTf).

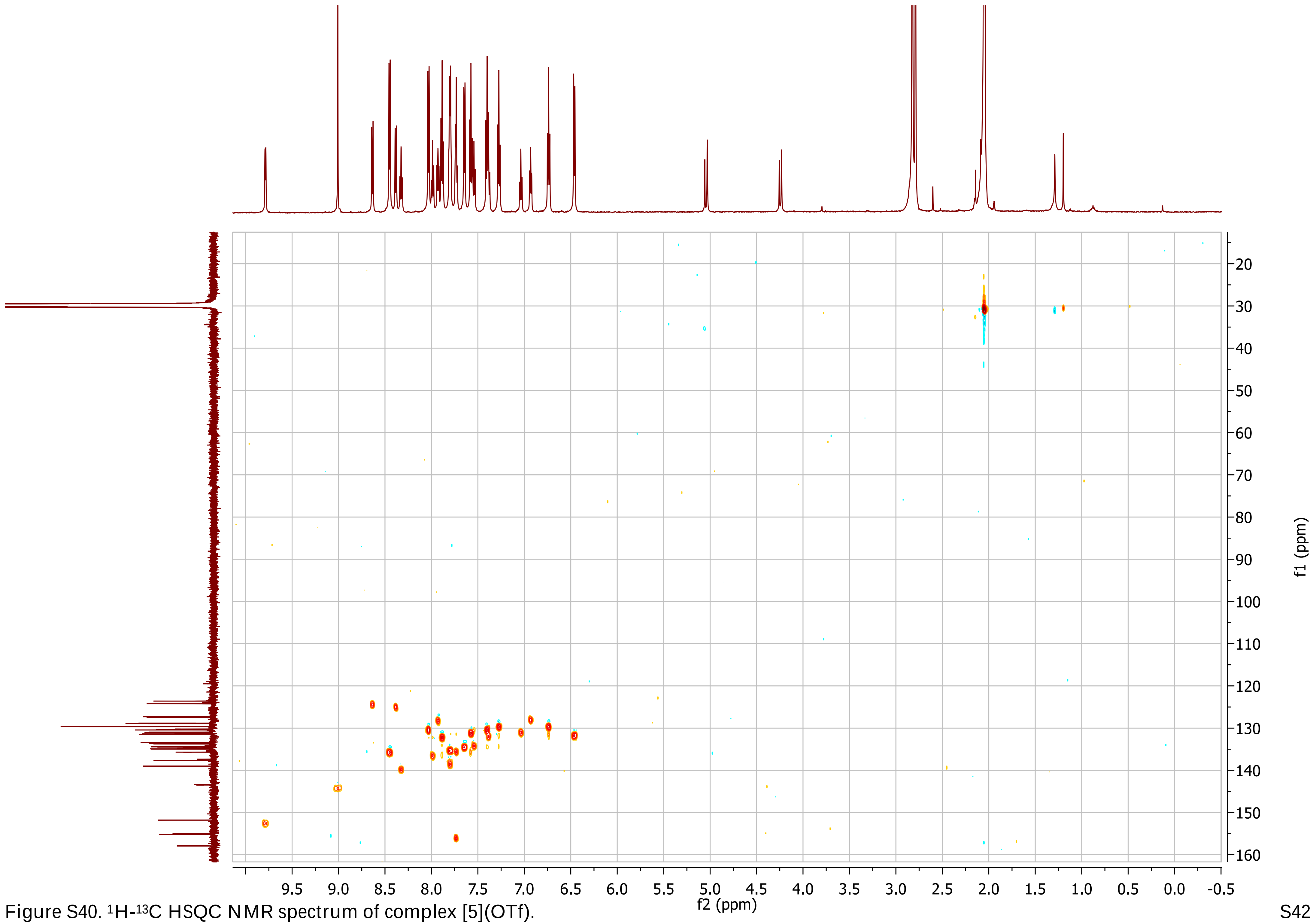

Figure S40. ^{1}H-^{13}C HSQC NMR spectrum of complex [5](OTf).

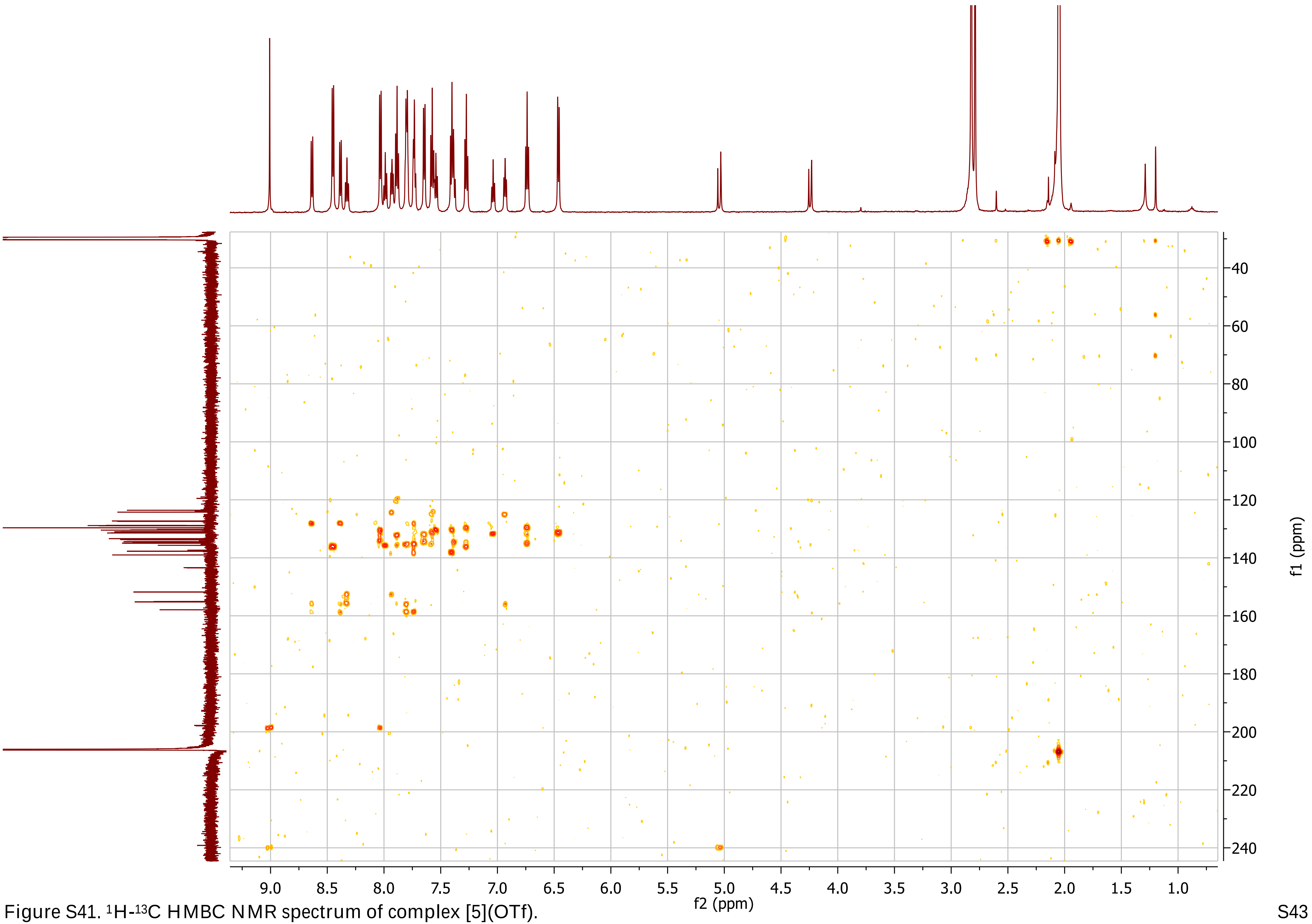

Figure S41. ^1H-^{13}C HMBC NMR spectrum of complex [5](OTf).

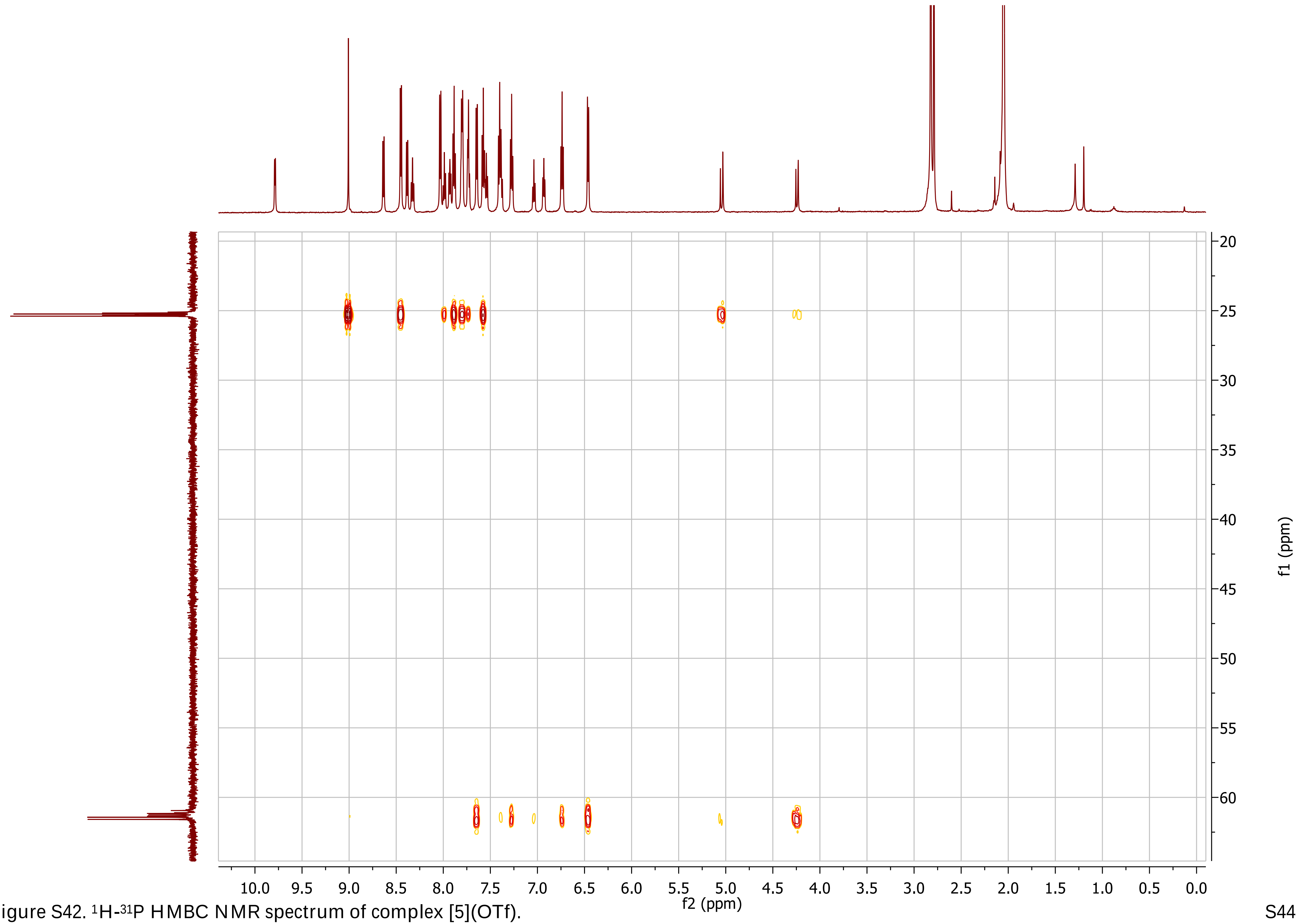

Figure S42. ^{1}H-^{31}P HMBC NMR spectrum of complex [5](OTf).

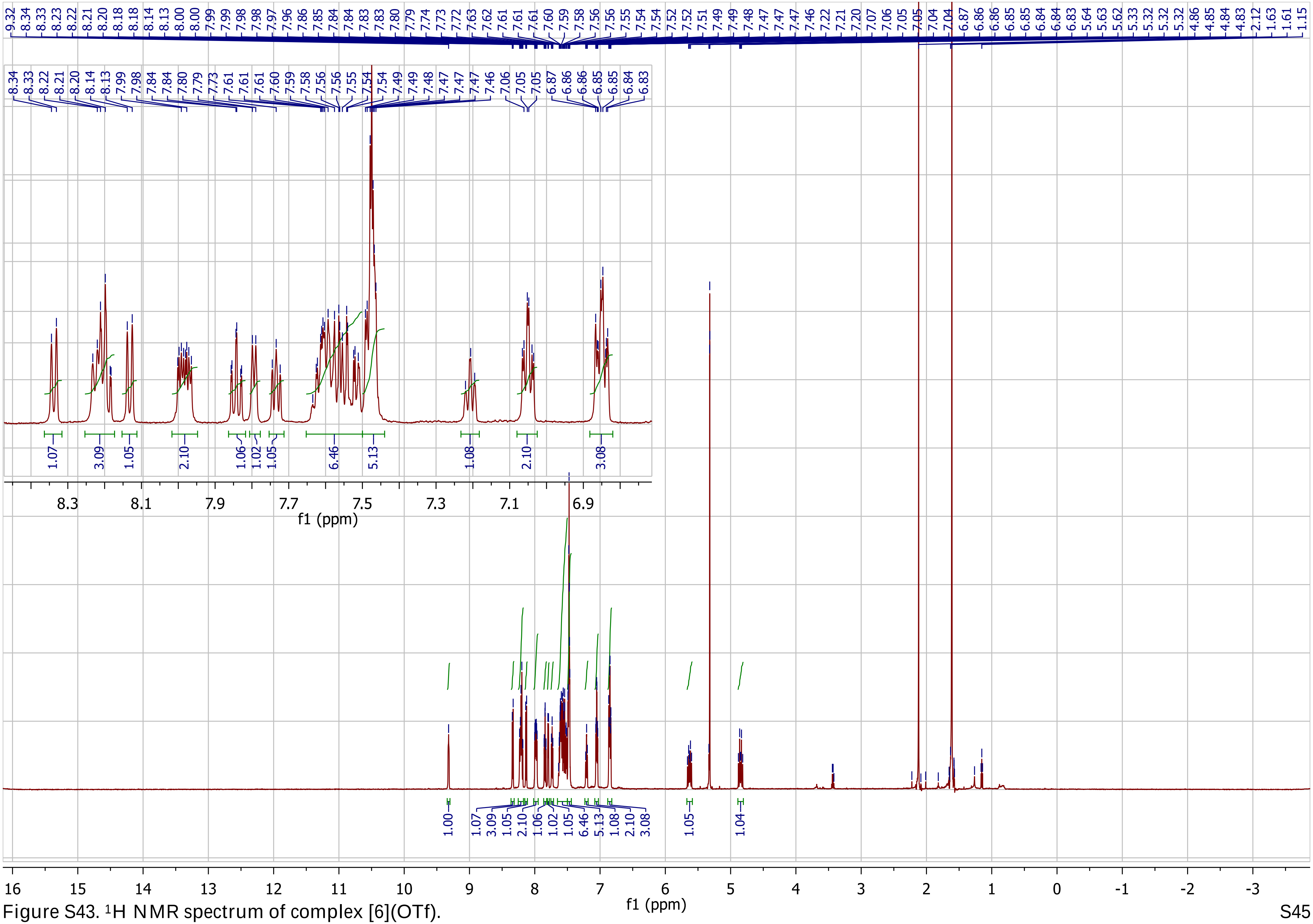

Figure S43. ¹H NMR spectrum of complex [6](OTf).

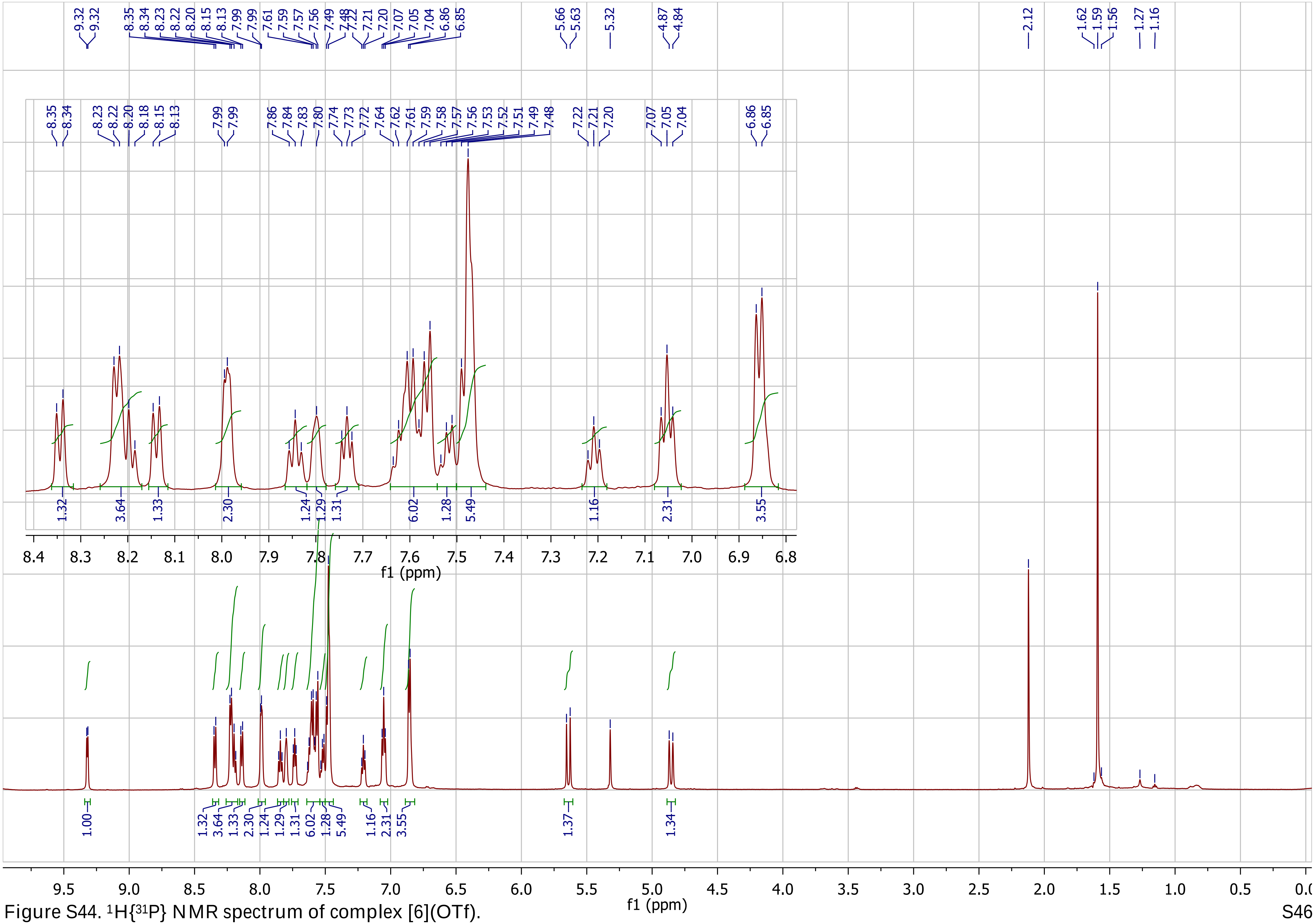

Figure S44. $^{1}H\{^{31}P\}$ NMR spectrum of complex [6](OTf).

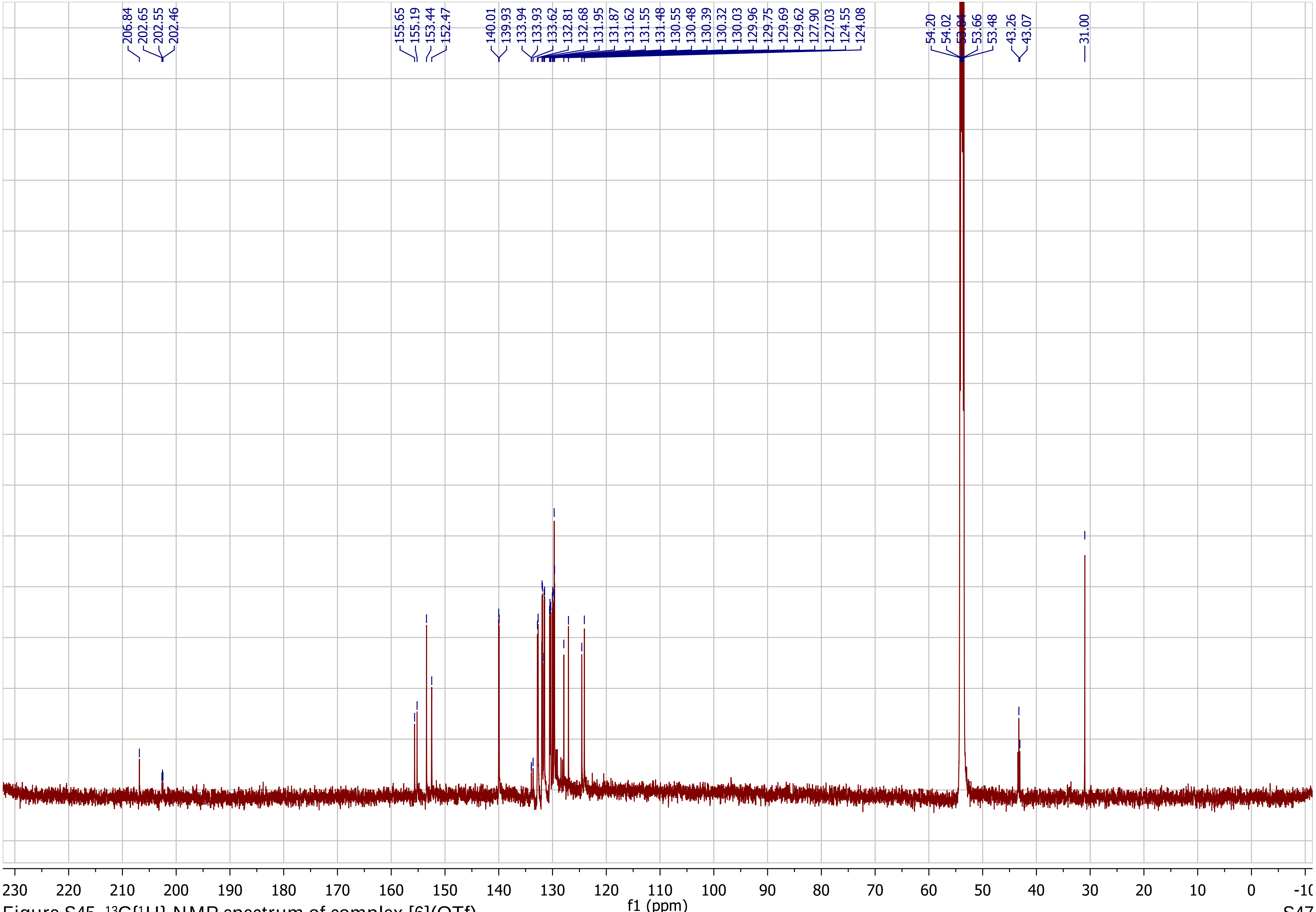

Figure S45. $^{13}C\{^{1}H\}$ NMR spectrum of complex [6](OTf).

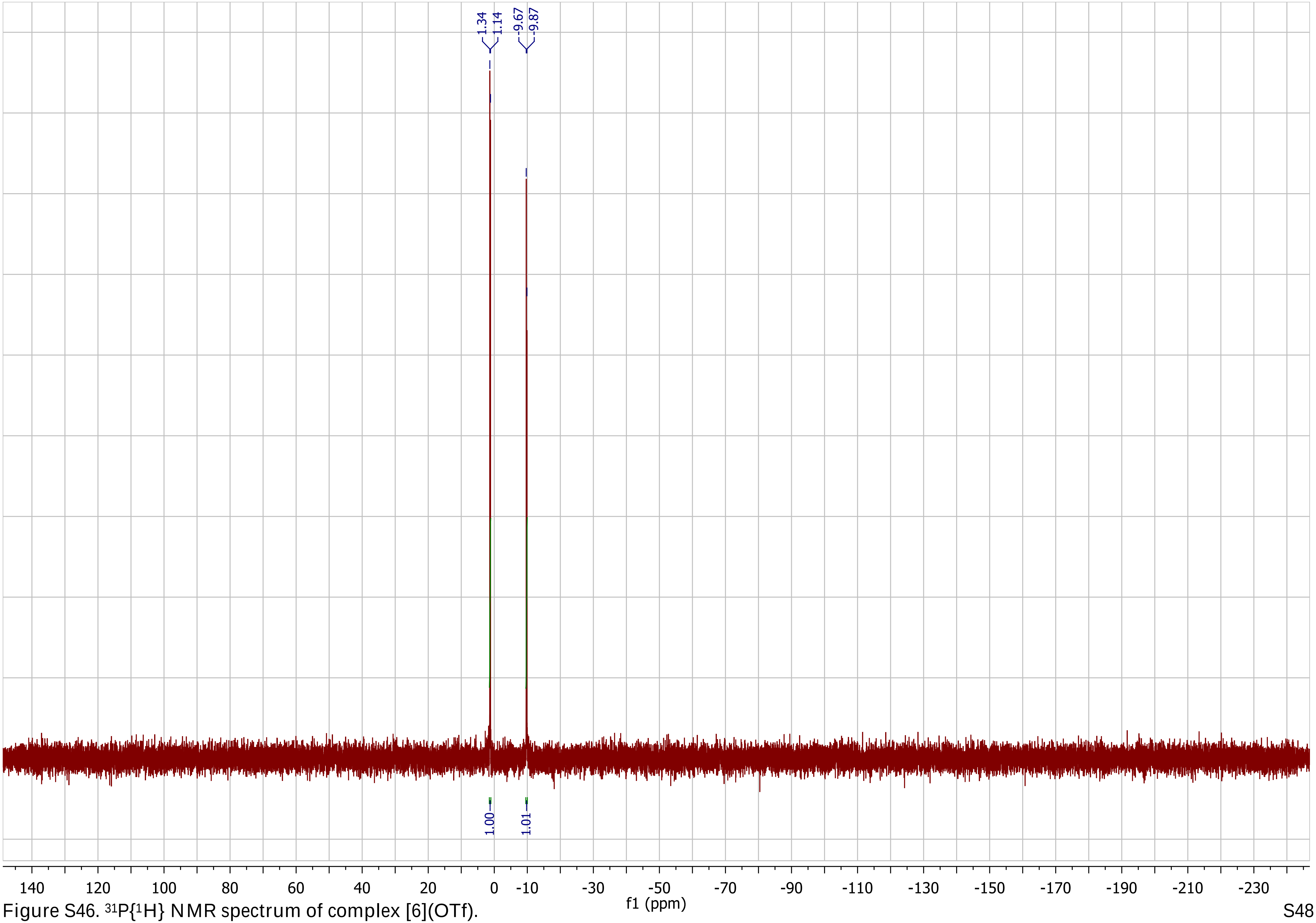

Figure S46. $^{31}P\{^{1}H\}$ NMR spectrum of complex [6](OTf).

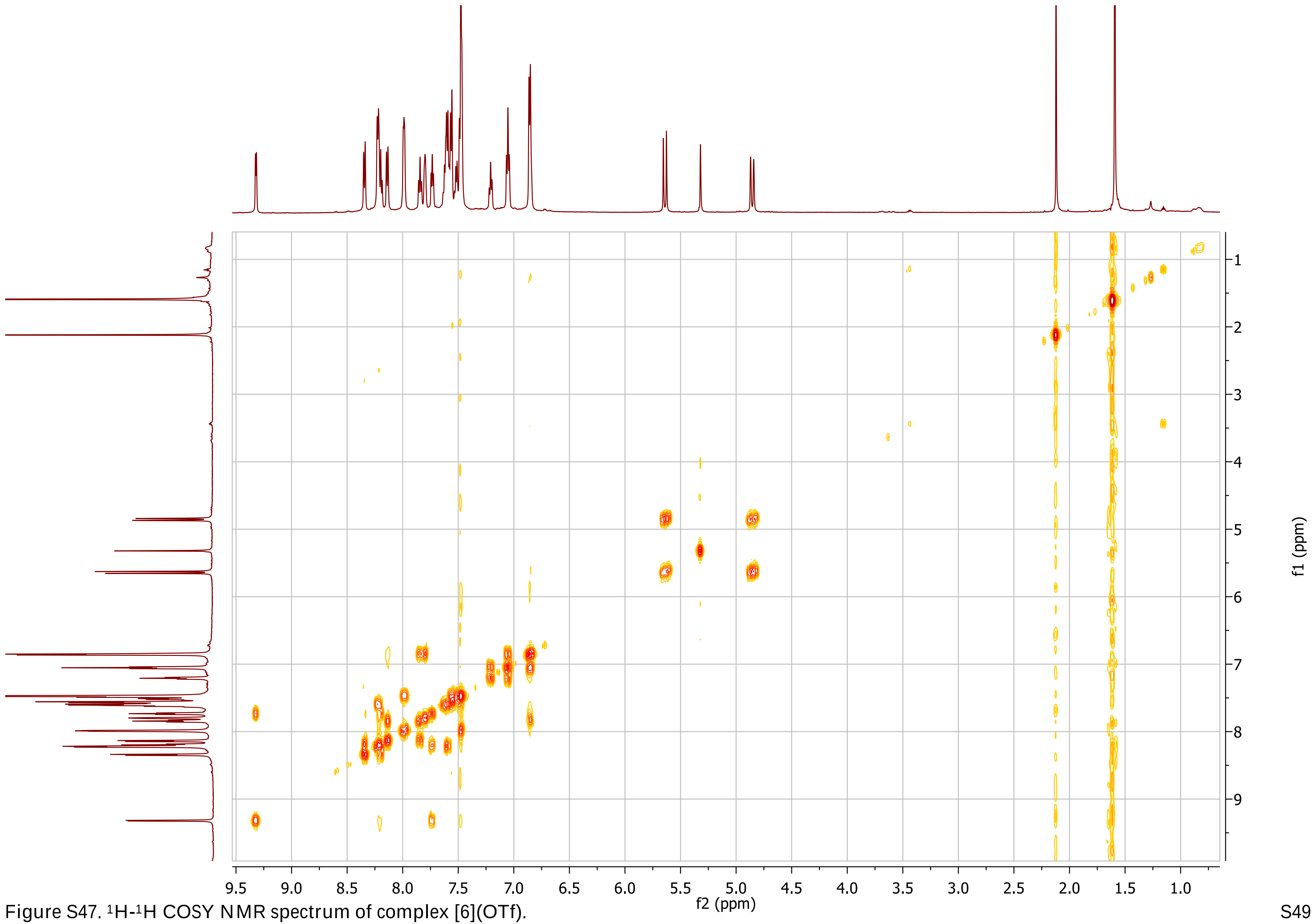

Figure S47. ^{1}H-^{1}H COSY NMR spectrum of complex [6](OTf).

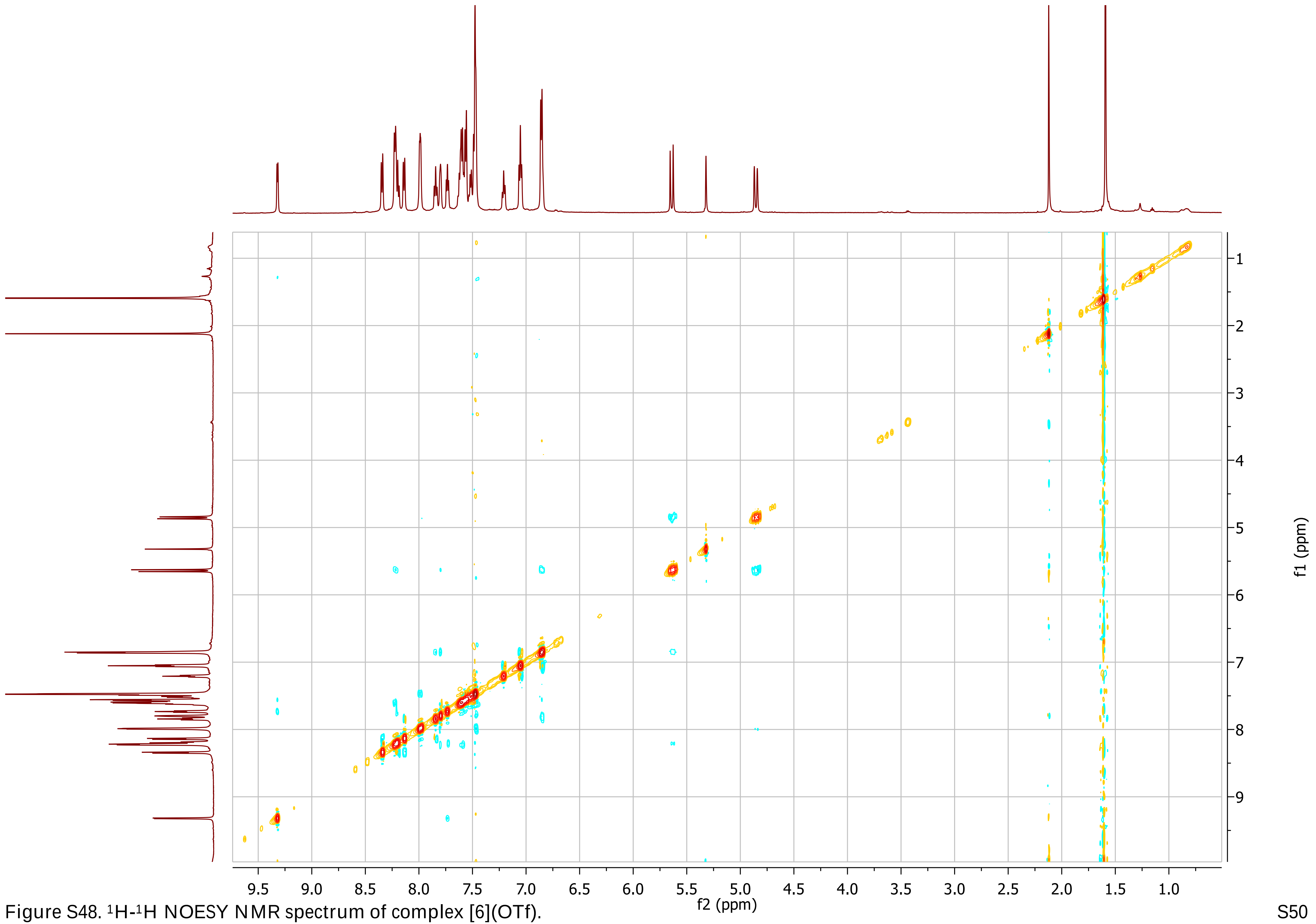

Figure S48. ^{1}H-^{1}H NOESY NMR spectrum of complex [6](OTf).

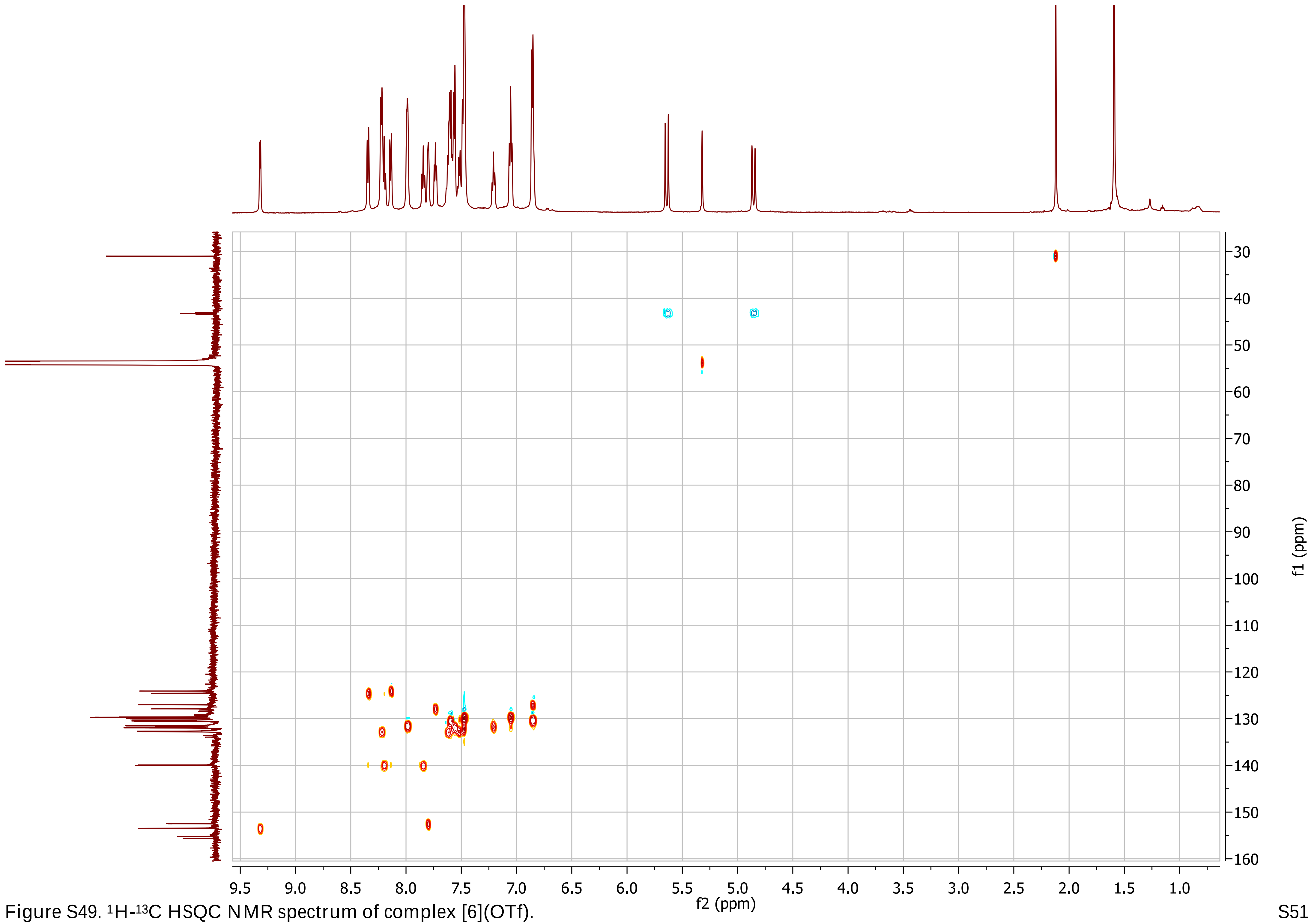

Figure S49. ^{1}H-^{13}C HSQC NMR spectrum of complex [6](OTf).

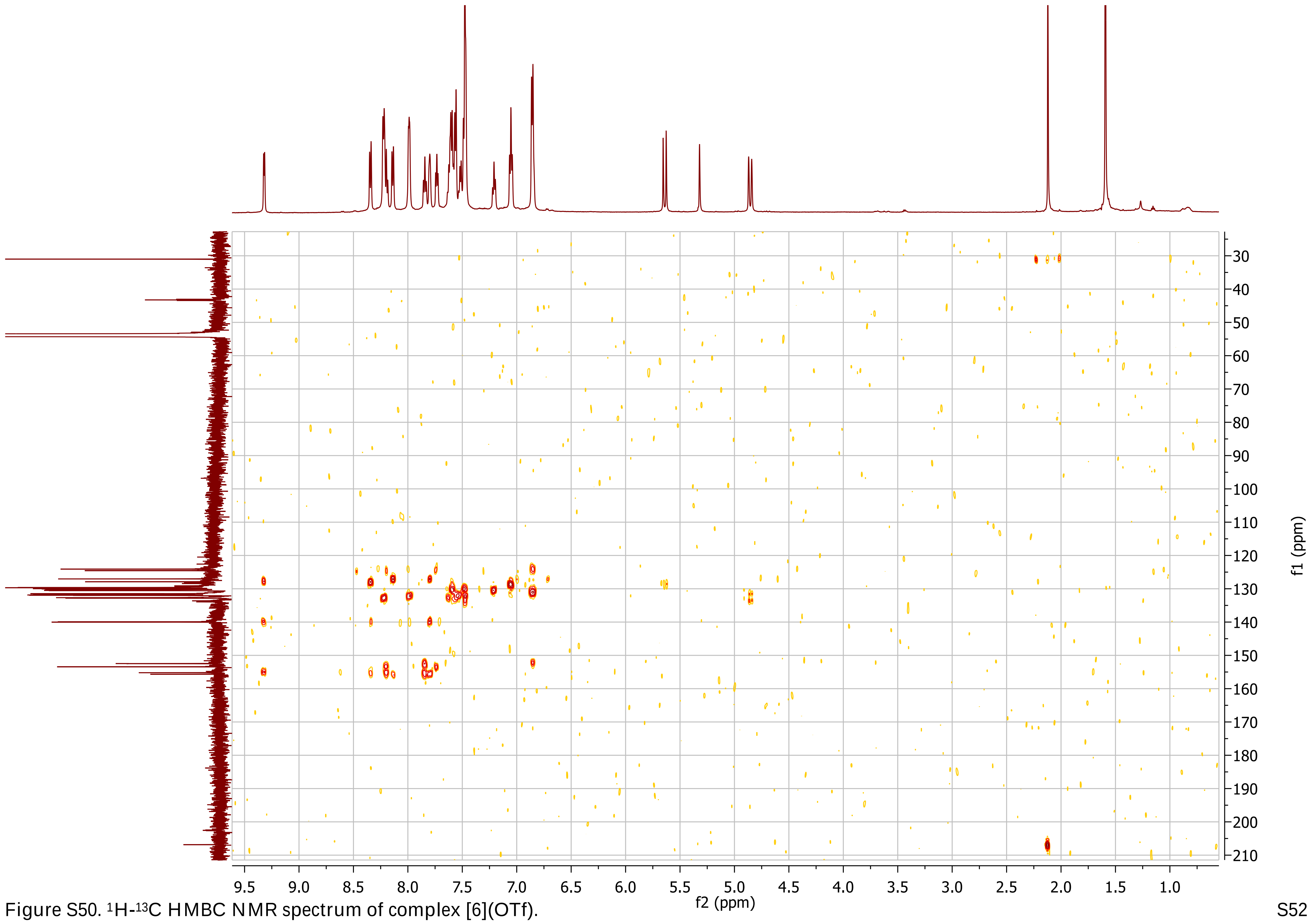

Figure S50. ^{1}H-^{13}C HMBC NMR spectrum of complex [6](OTf).

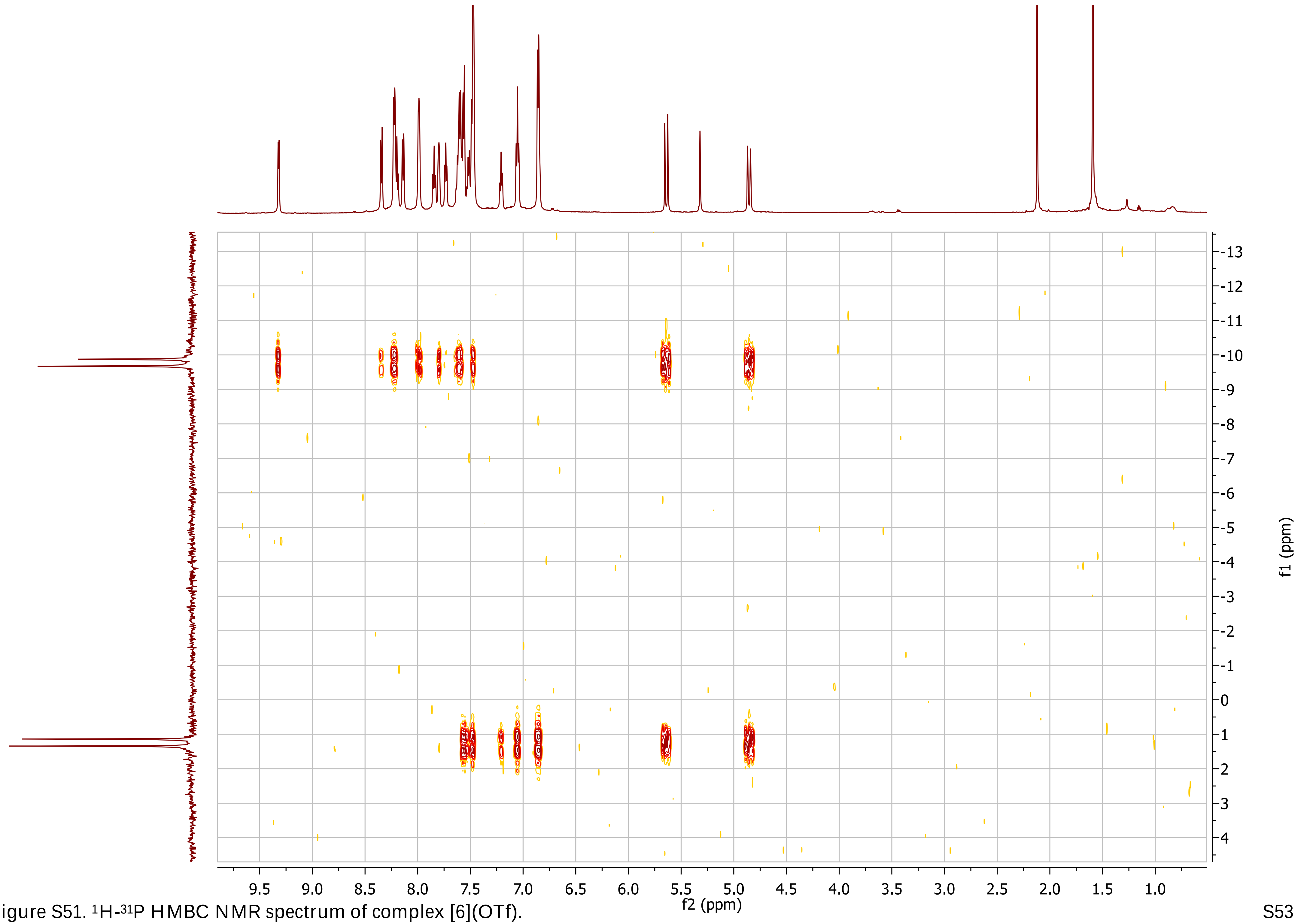

Figure S51. ^{1}H-^{31}P HMBC NMR spectrum of complex [6](OTf).

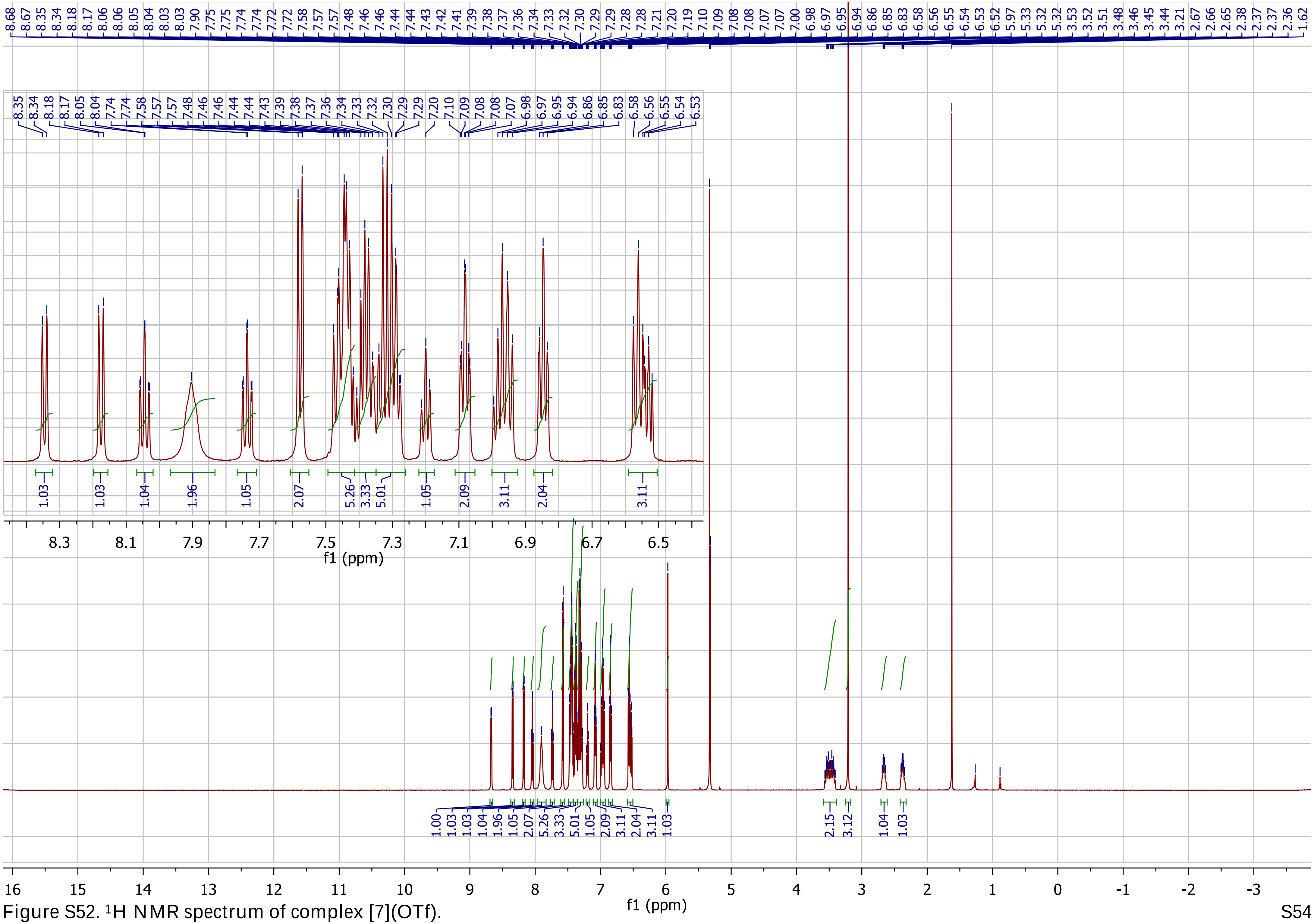

Figure S52. ¹H NMR spectrum of complex [7](OTf).

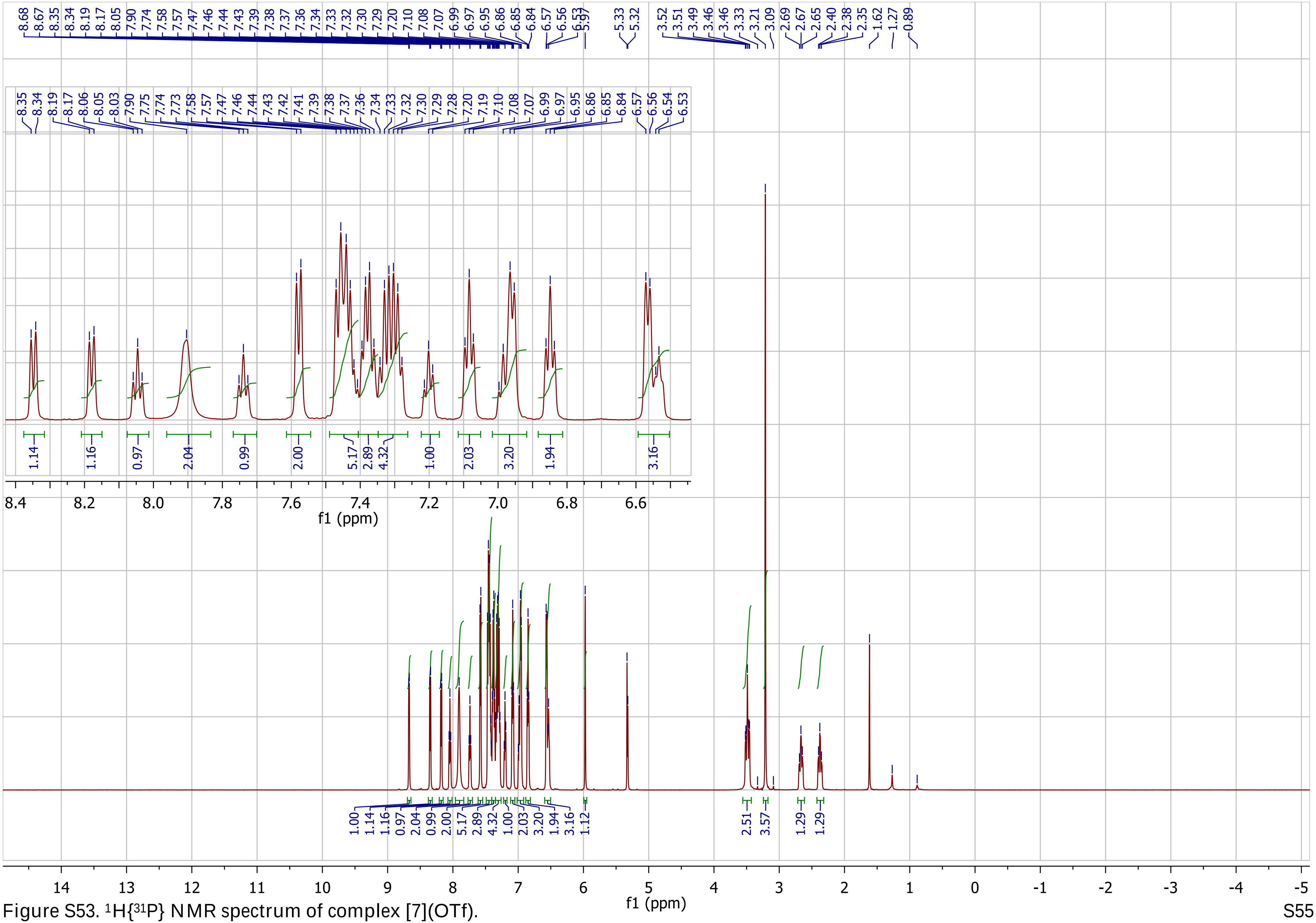

Figure S53. $^{1}H\{^{31}P\}$ NMR spectrum of complex [7](OTf).

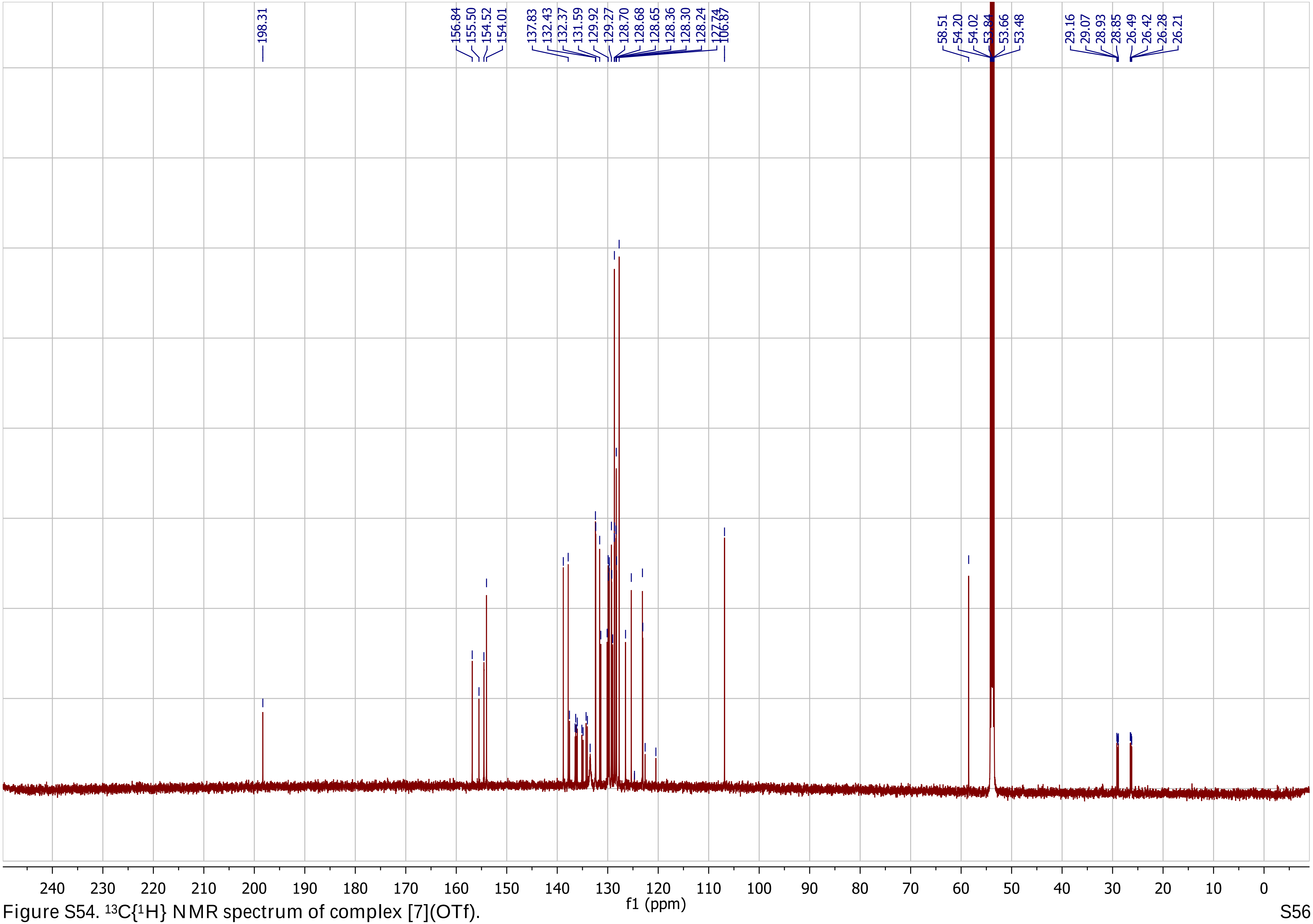

Figure S54. $^{13}C\{^{1}H\}$ NMR spectrum of complex [7](OTf).

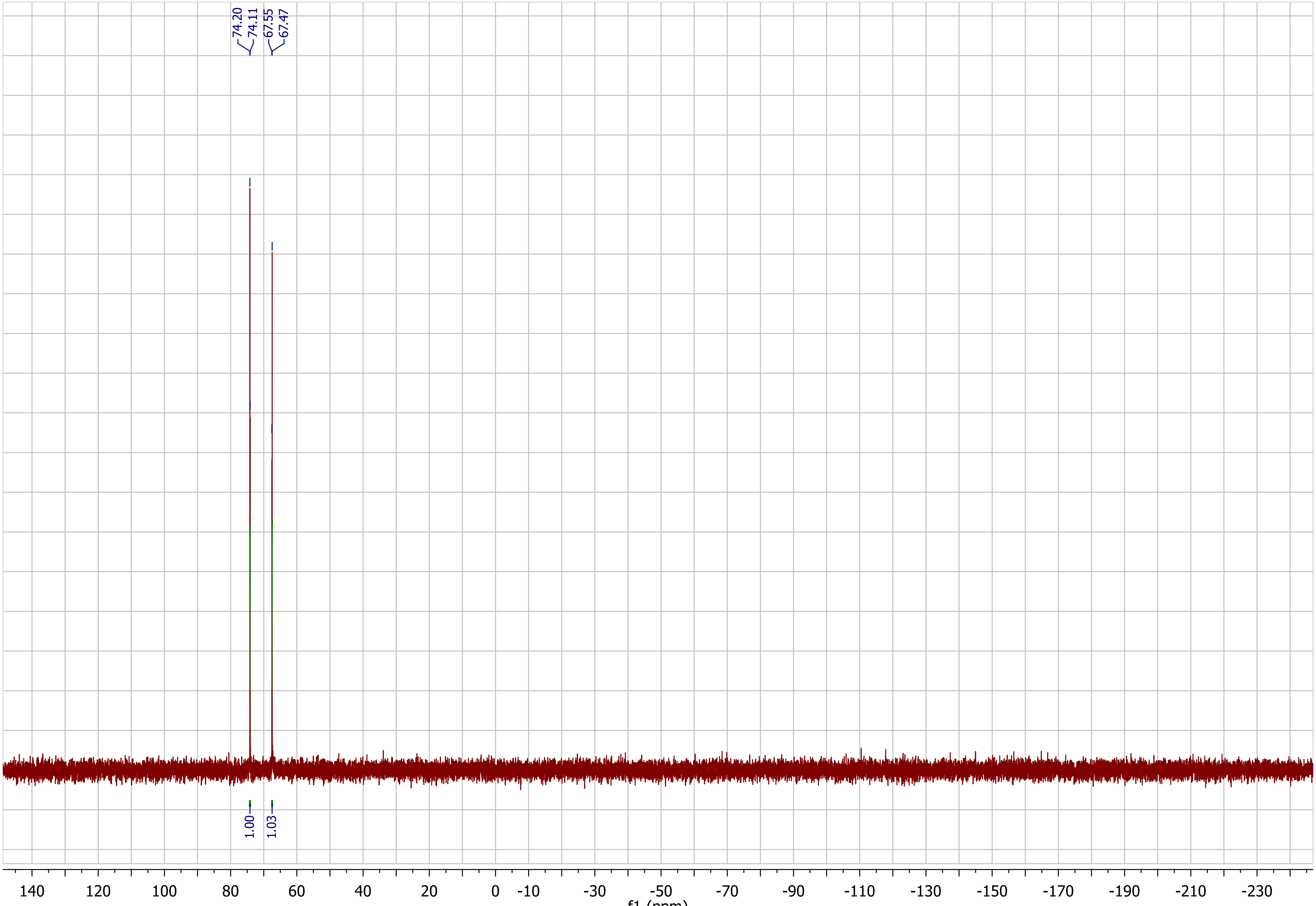

Figure S55. $^{31}P\{^{1}H\}$ NMR spectrum of complex [7](OTf).

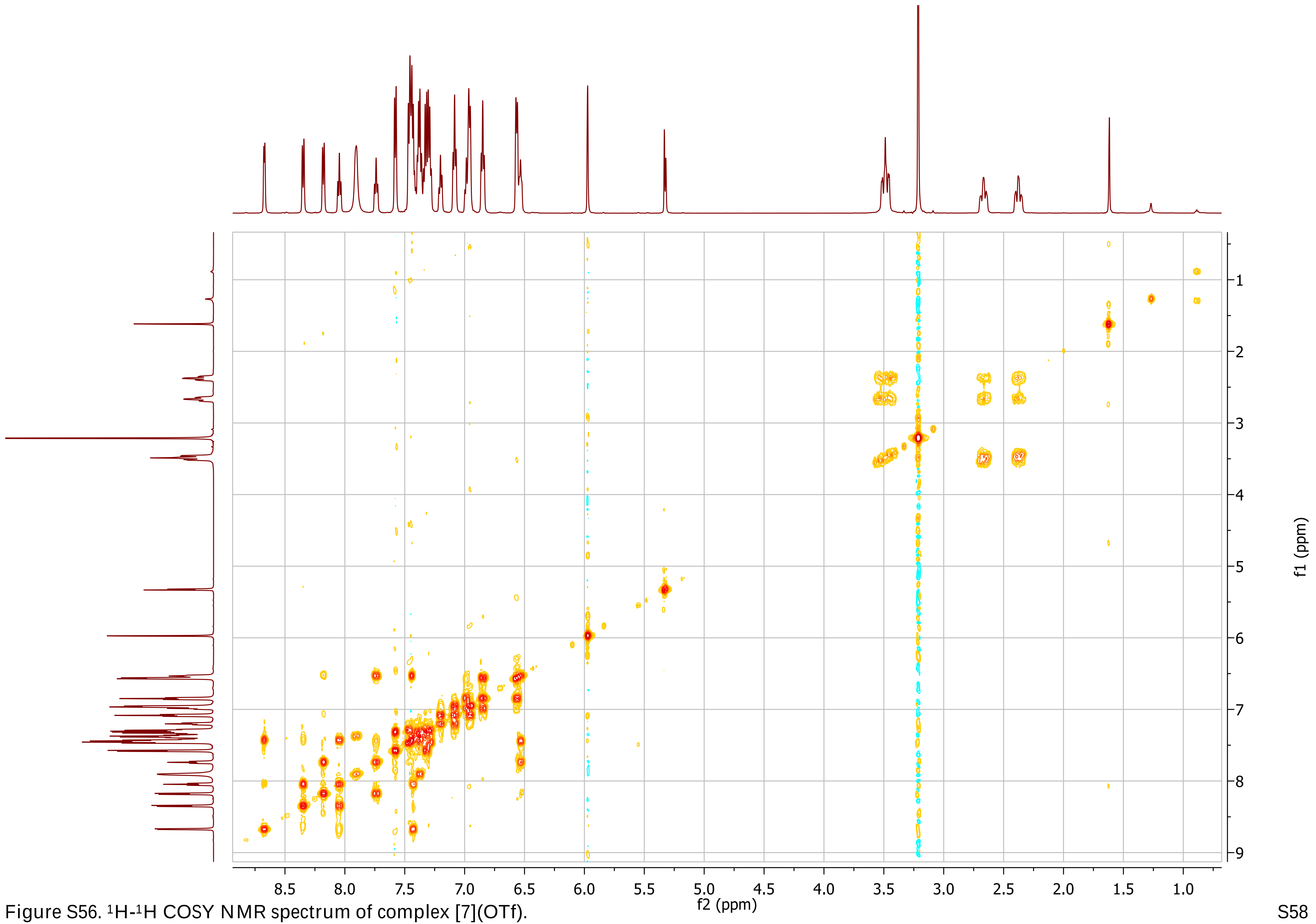

Figure S56. ^{1}H-^{1}H COSY NMR spectrum of complex [7](OTf).

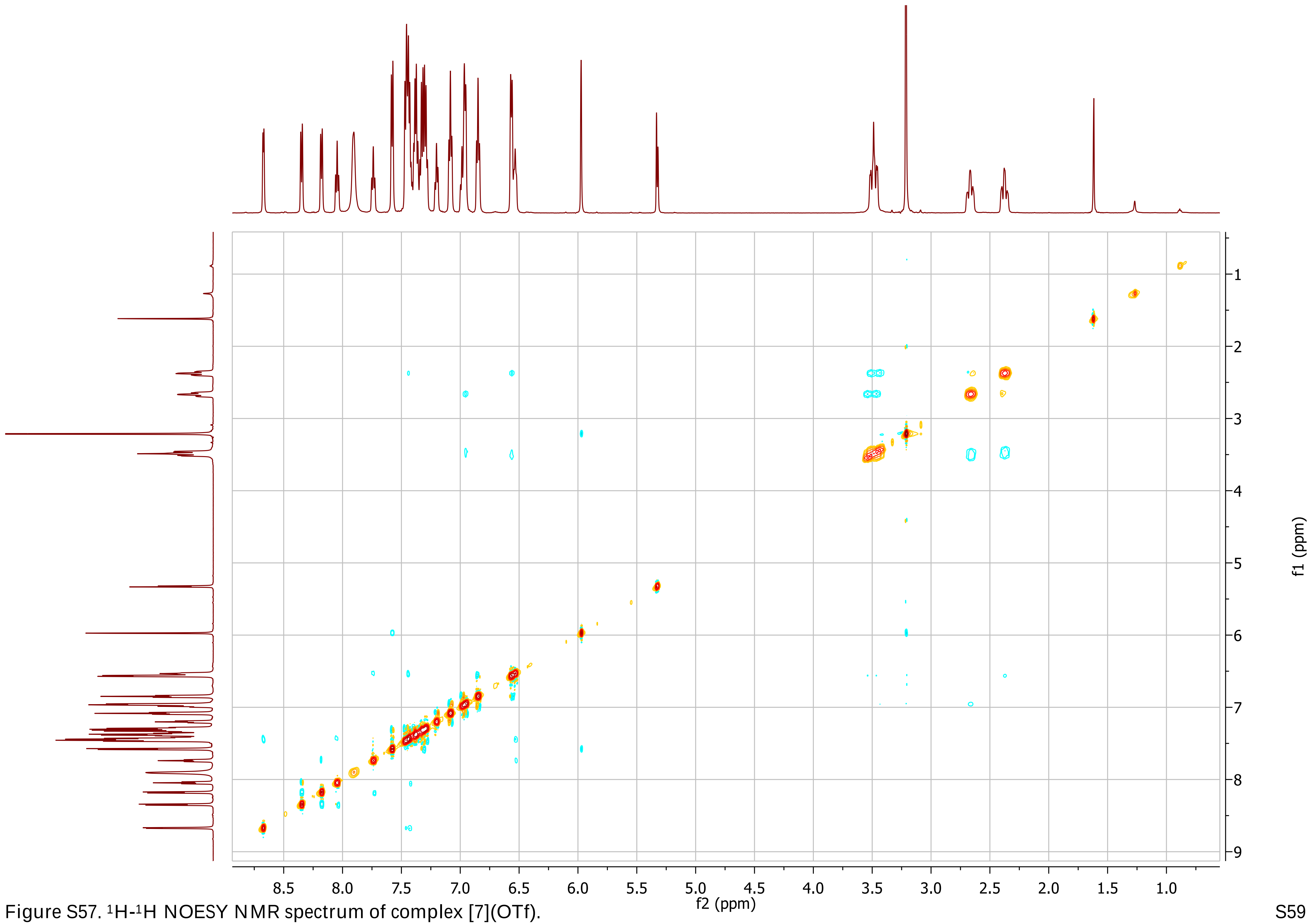

Figure S57. ^{1}H-^{1}H NOESY NMR spectrum of complex [7](OTf).

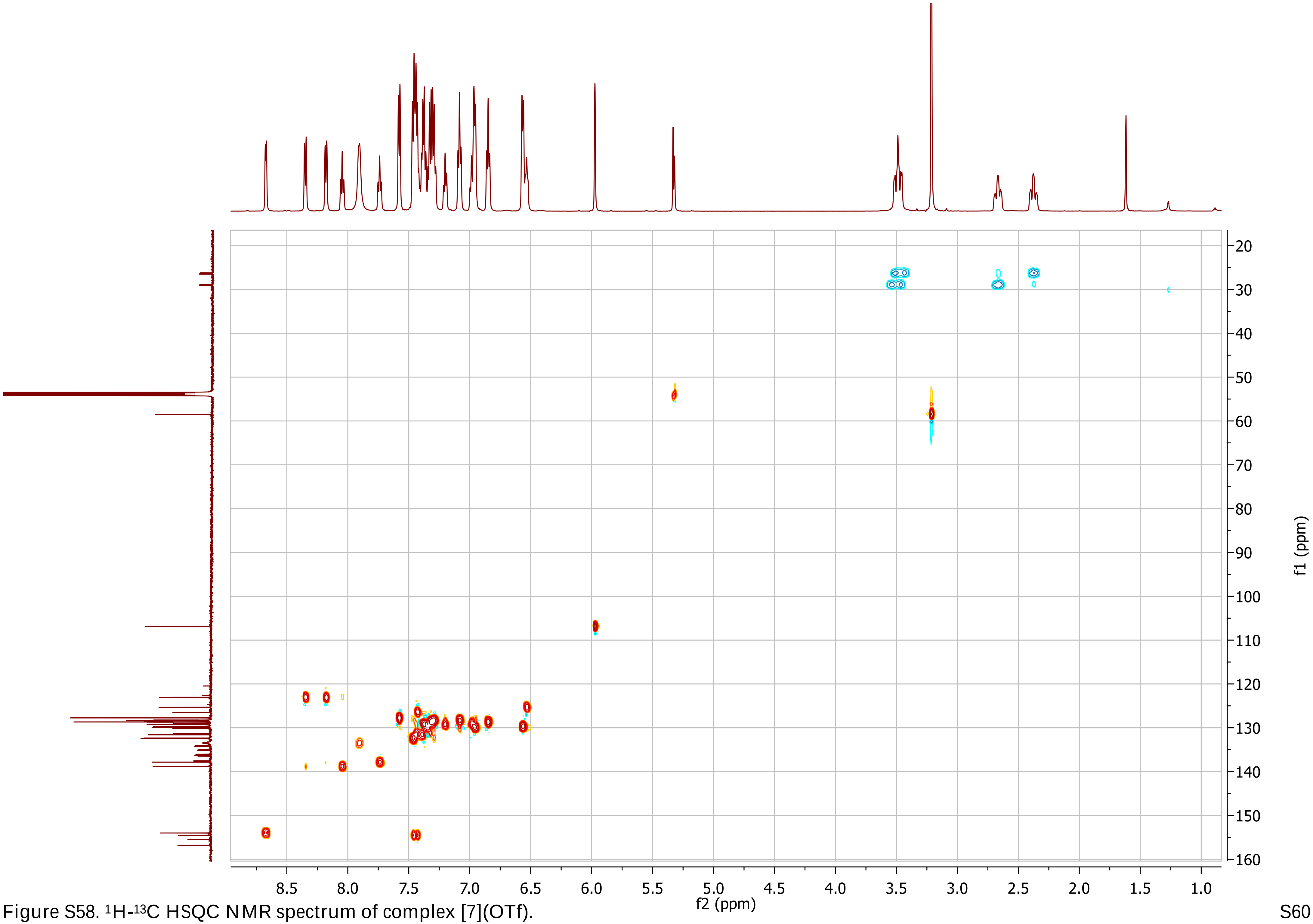

Figure S58. ^{1}H-^{13}C HSQC NMR spectrum of complex [7](OTf).

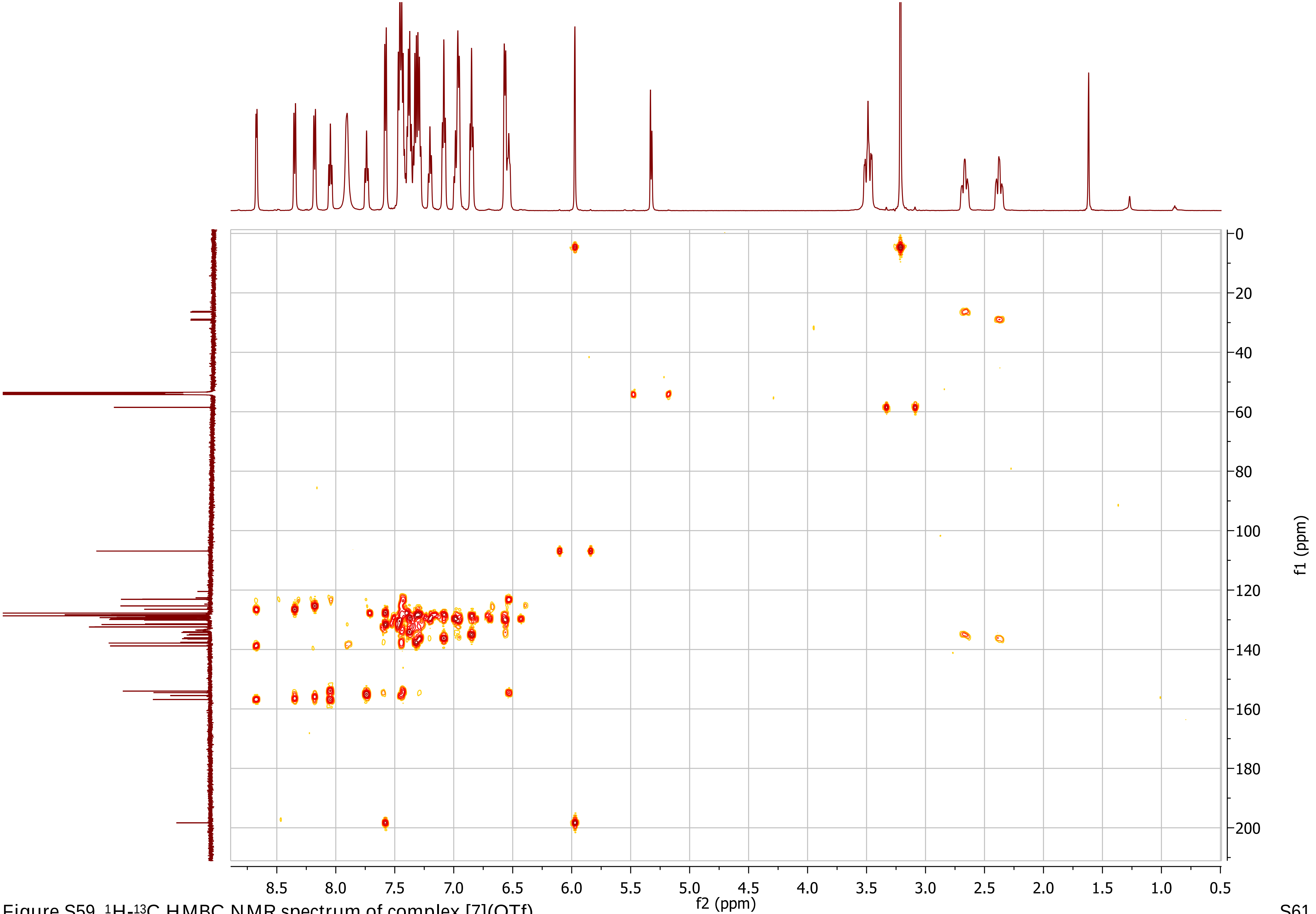

Figure S59. ^{1}H-^{13}C HMBC NMR spectrum of complex [7](OTf).

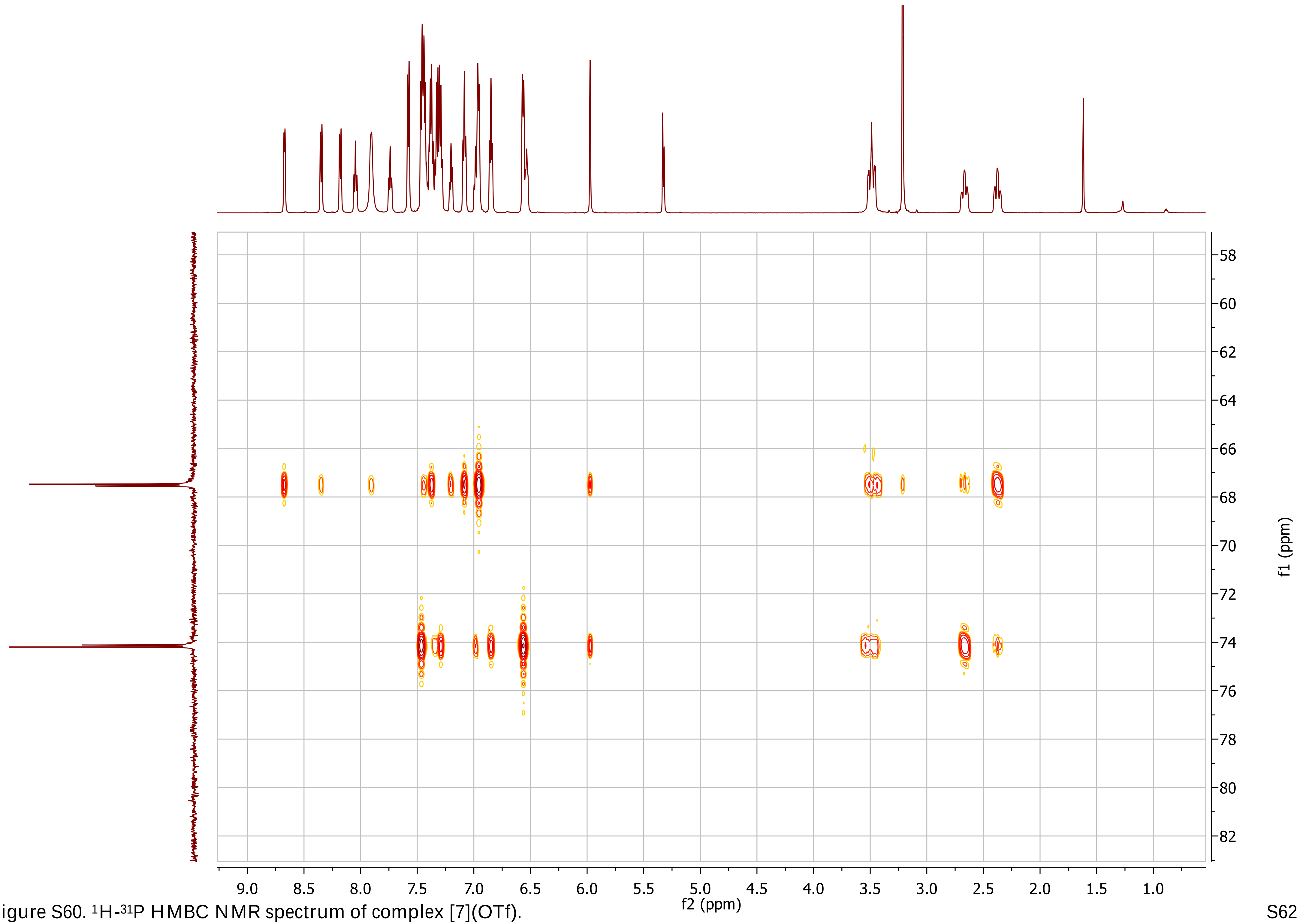

Figure S60. ^{1}H-^{31}P HMBC NMR spectrum of complex [7](OTf).

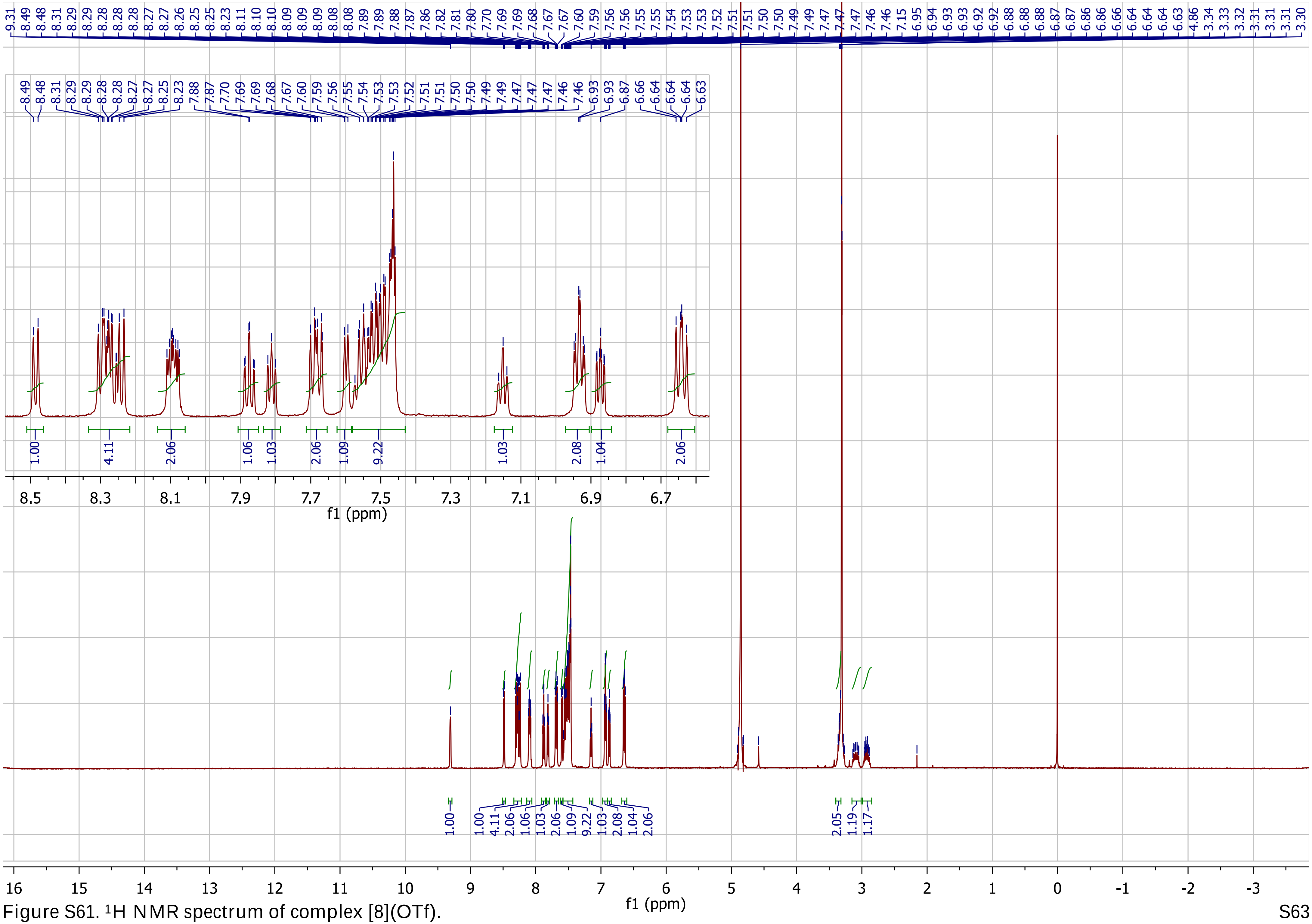

Figure S61. ^{1}H NMR spectrum of complex [8](OTf).

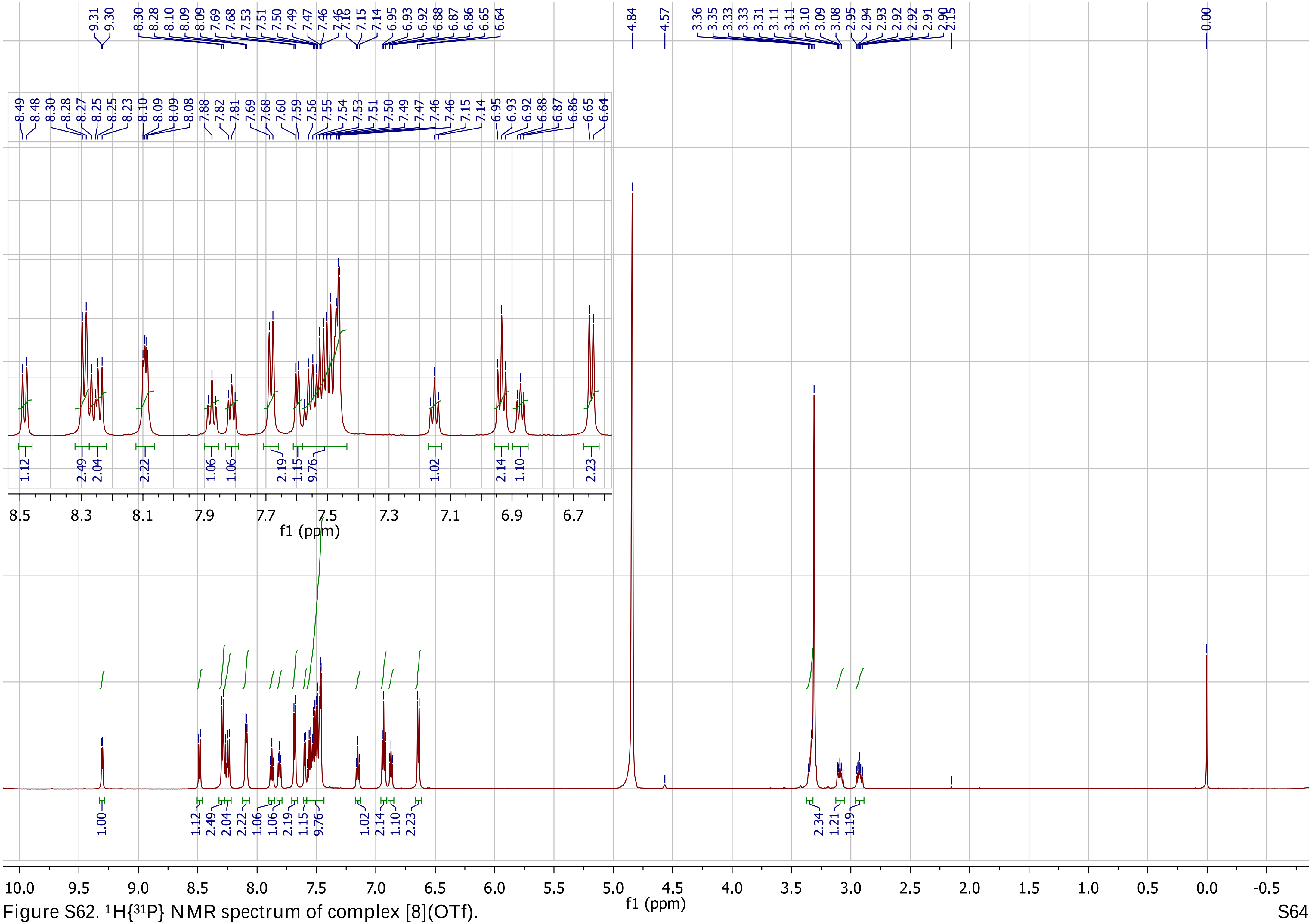

Figure S62. $^{1}H\{^{31}P\}$ NMR spectrum of complex [8](OTf).

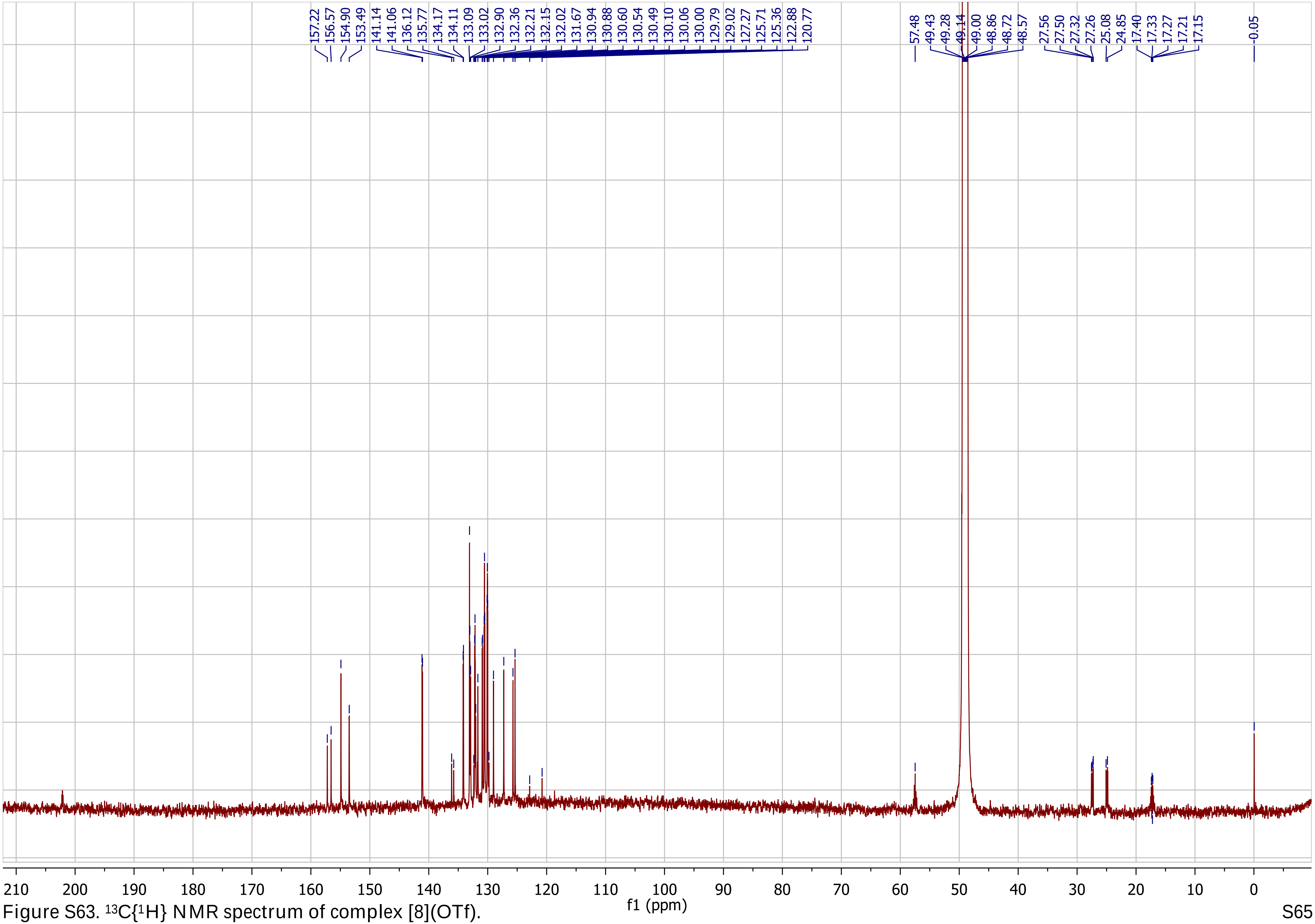

Figure S63. $^{13}C\{^{1}H\}$ NMR spectrum of complex [8](OTf).

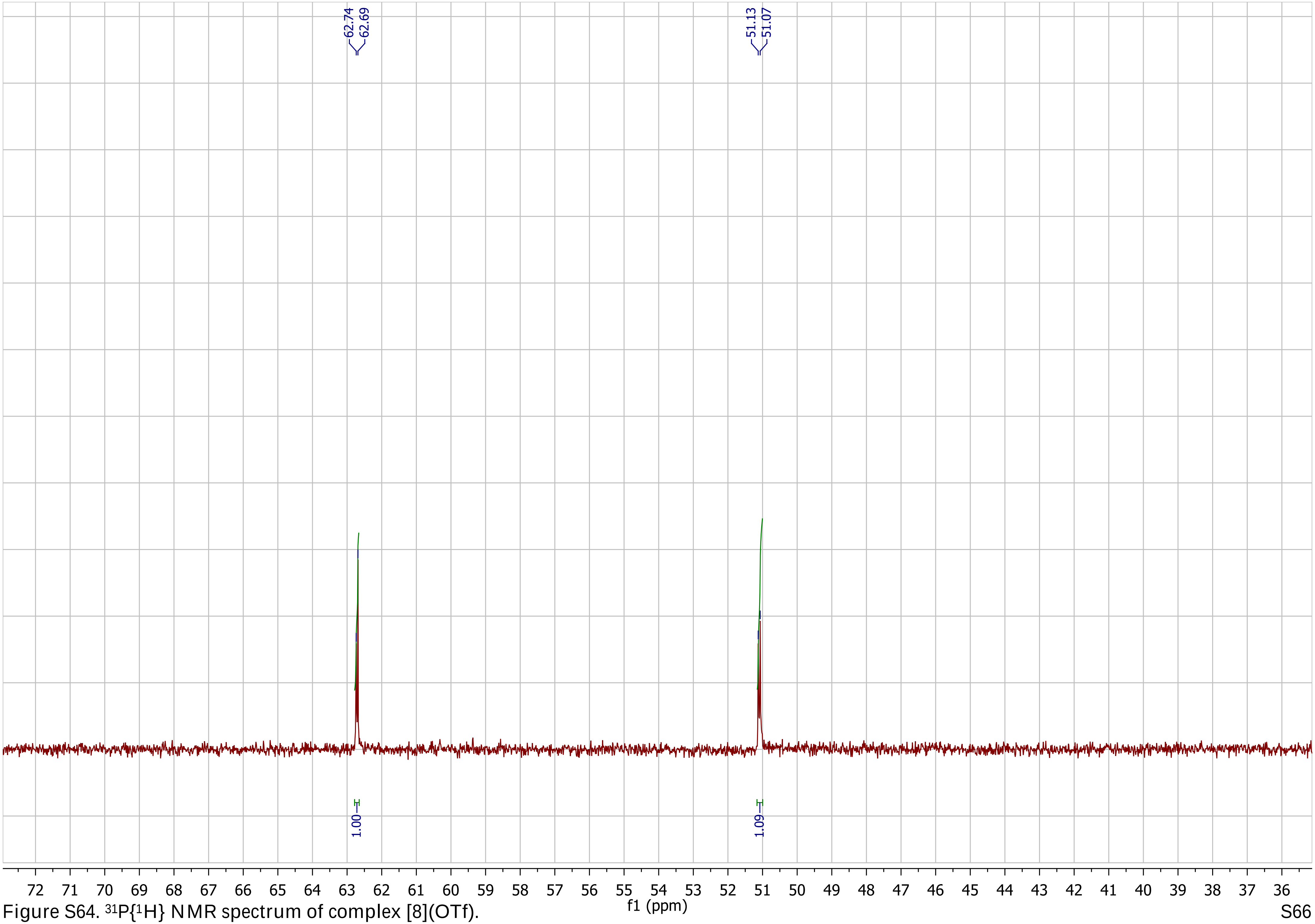

Figure S64. $^{31}P\{^{1}H\}$ NMR spectrum of complex [8](OTf).

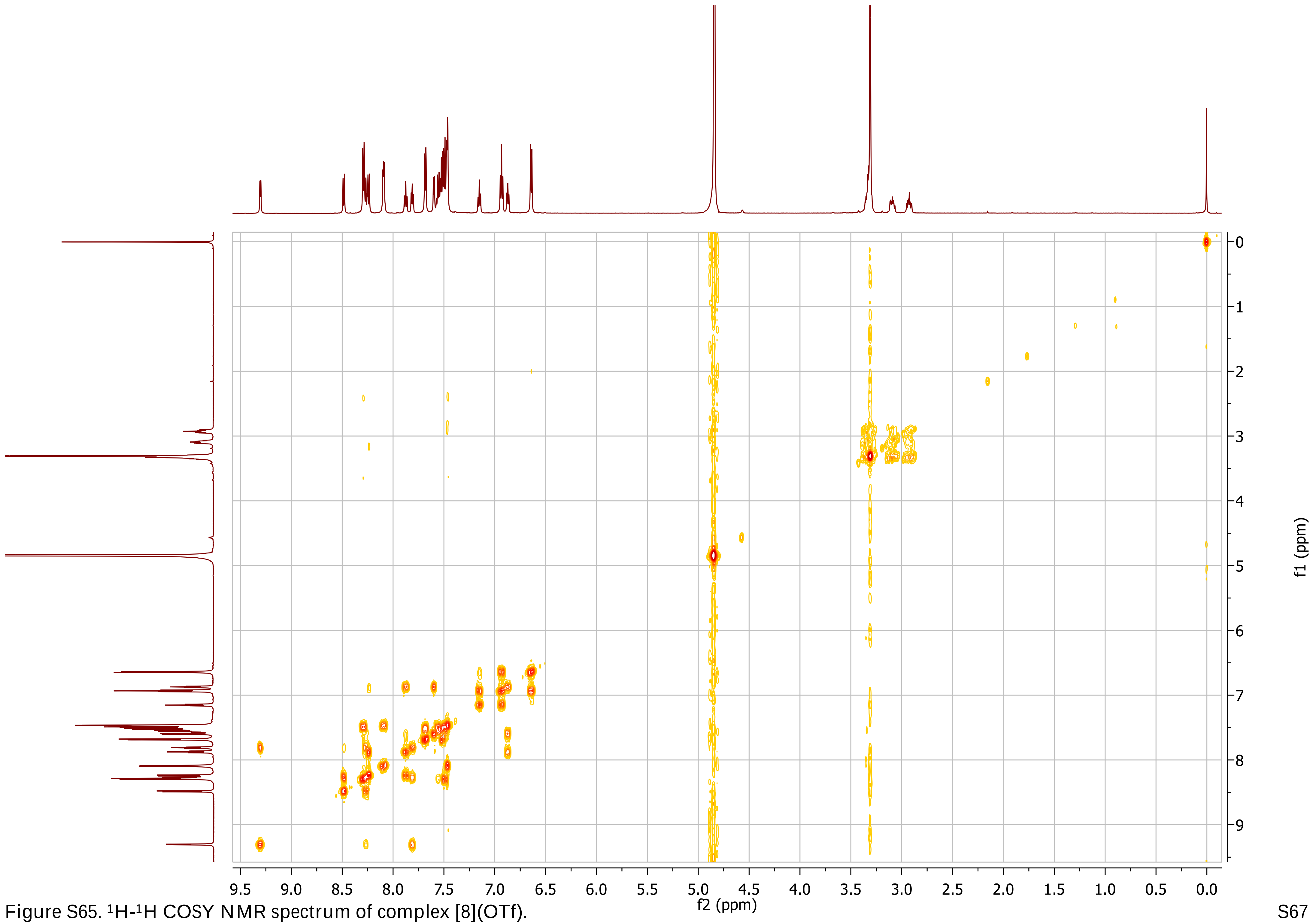

Figure S65. ^{1}H-^{1}H COSY NMR spectrum of complex [8](OTf).

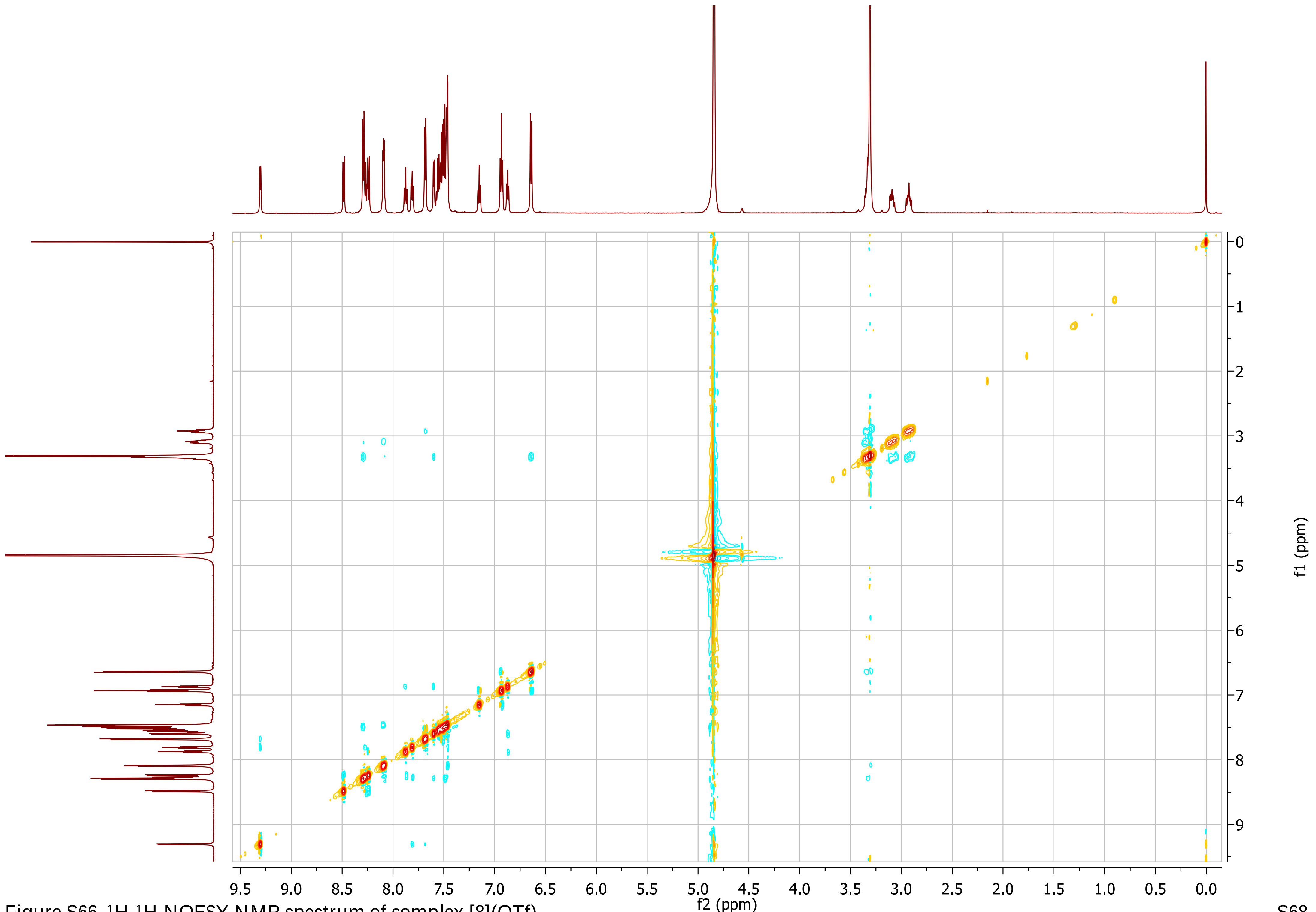

Figure S66. ^1H-^1H NOESY NMR spectrum of complex [8](OTf).

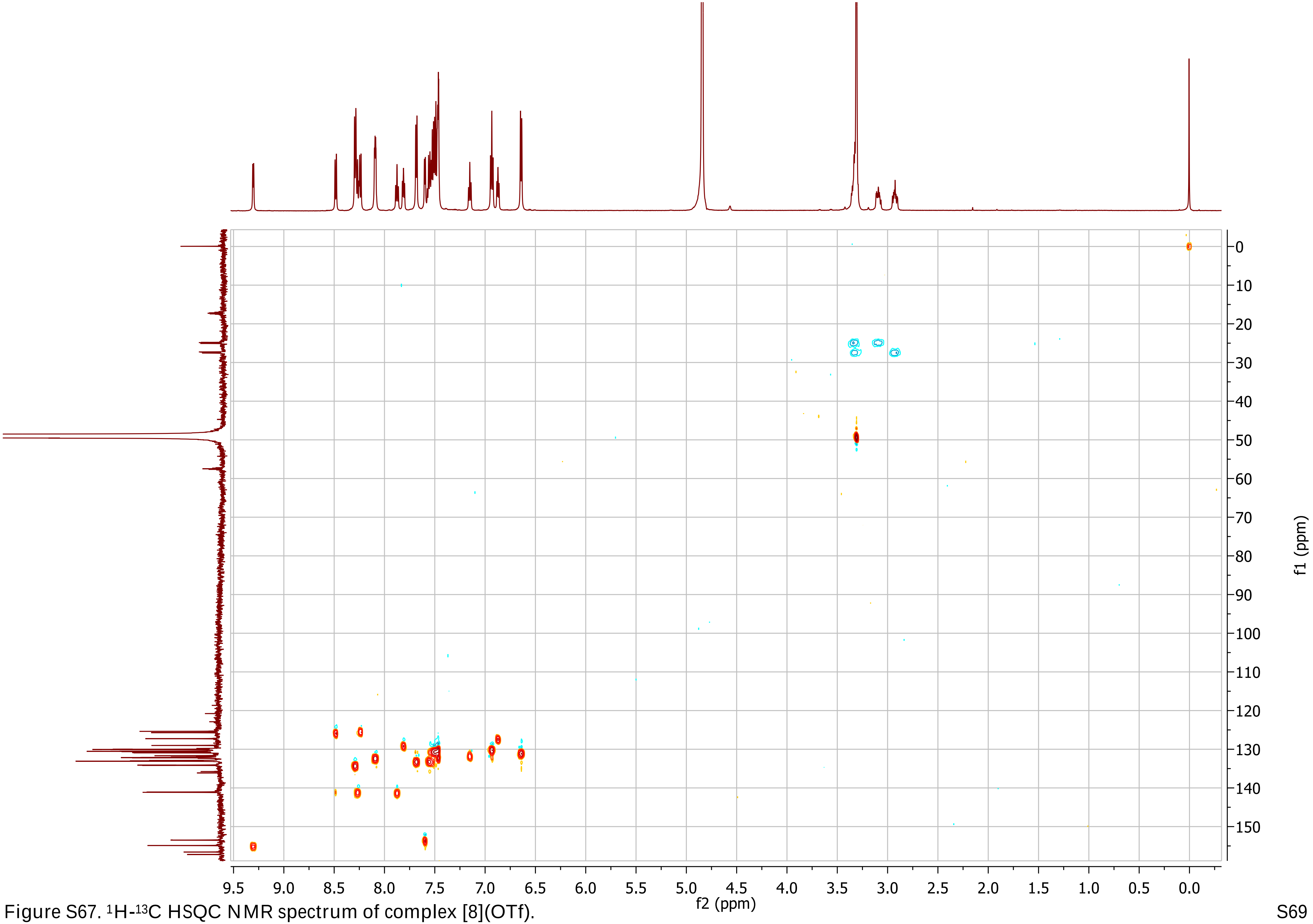

Figure S67. ^{1}H-^{13}C HSQC NMR spectrum of complex [8](OTf).

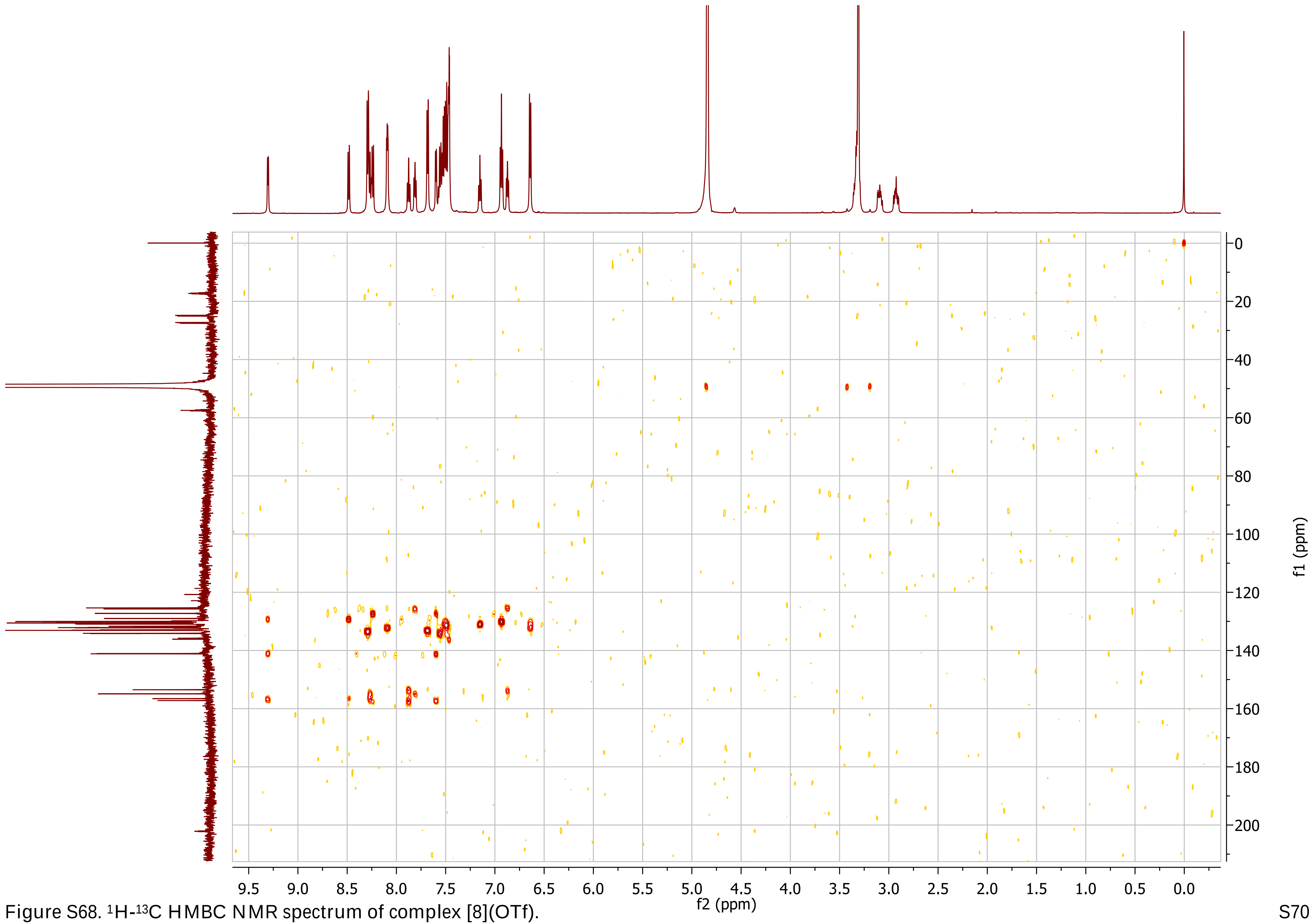

Figure S68. ^{1}H-^{13}C HMBC NMR spectrum of complex [8](OTf).

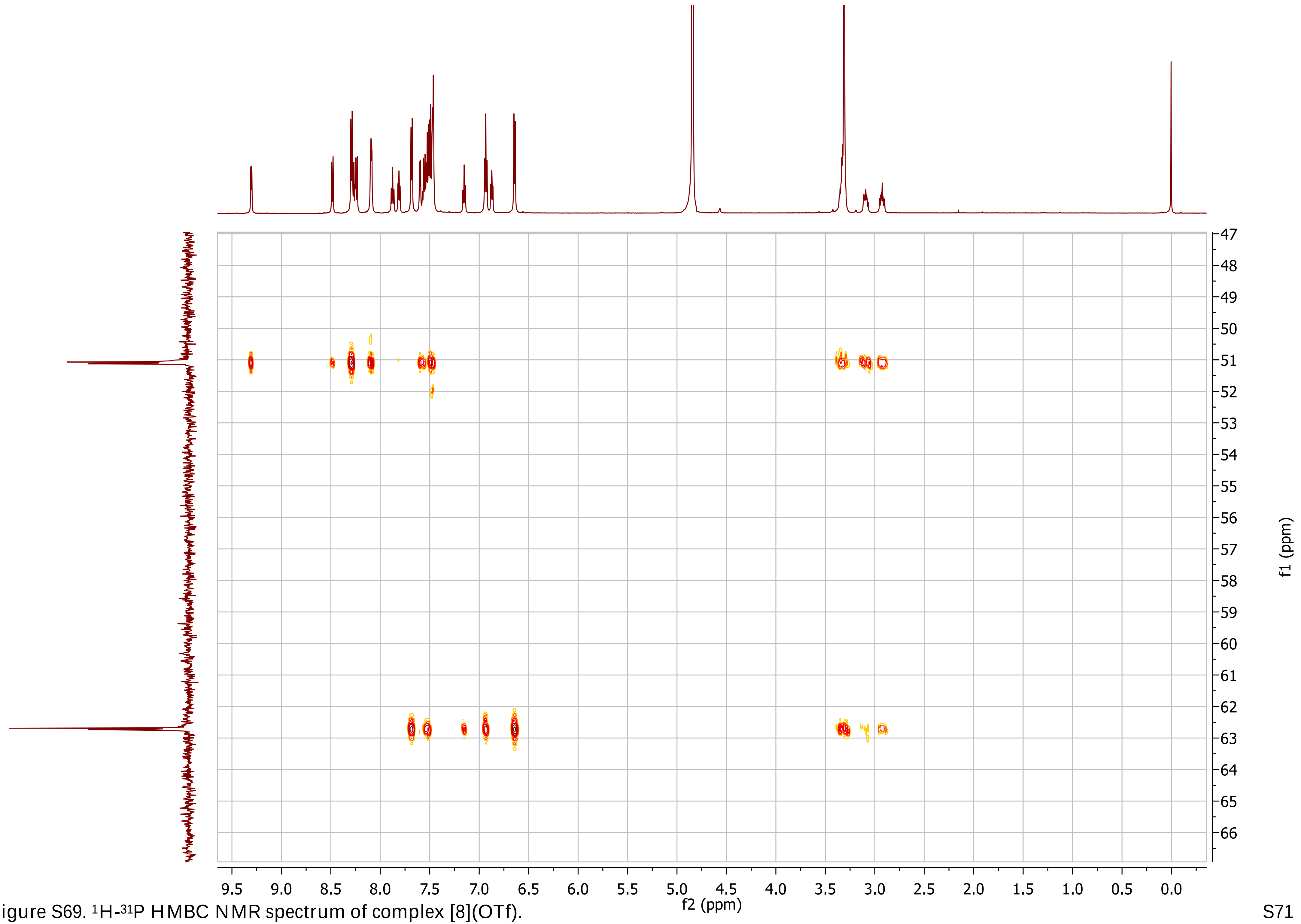

Figure S69. ^1H-^{31}P HMBC NMR spectrum of complex [8](OTf).

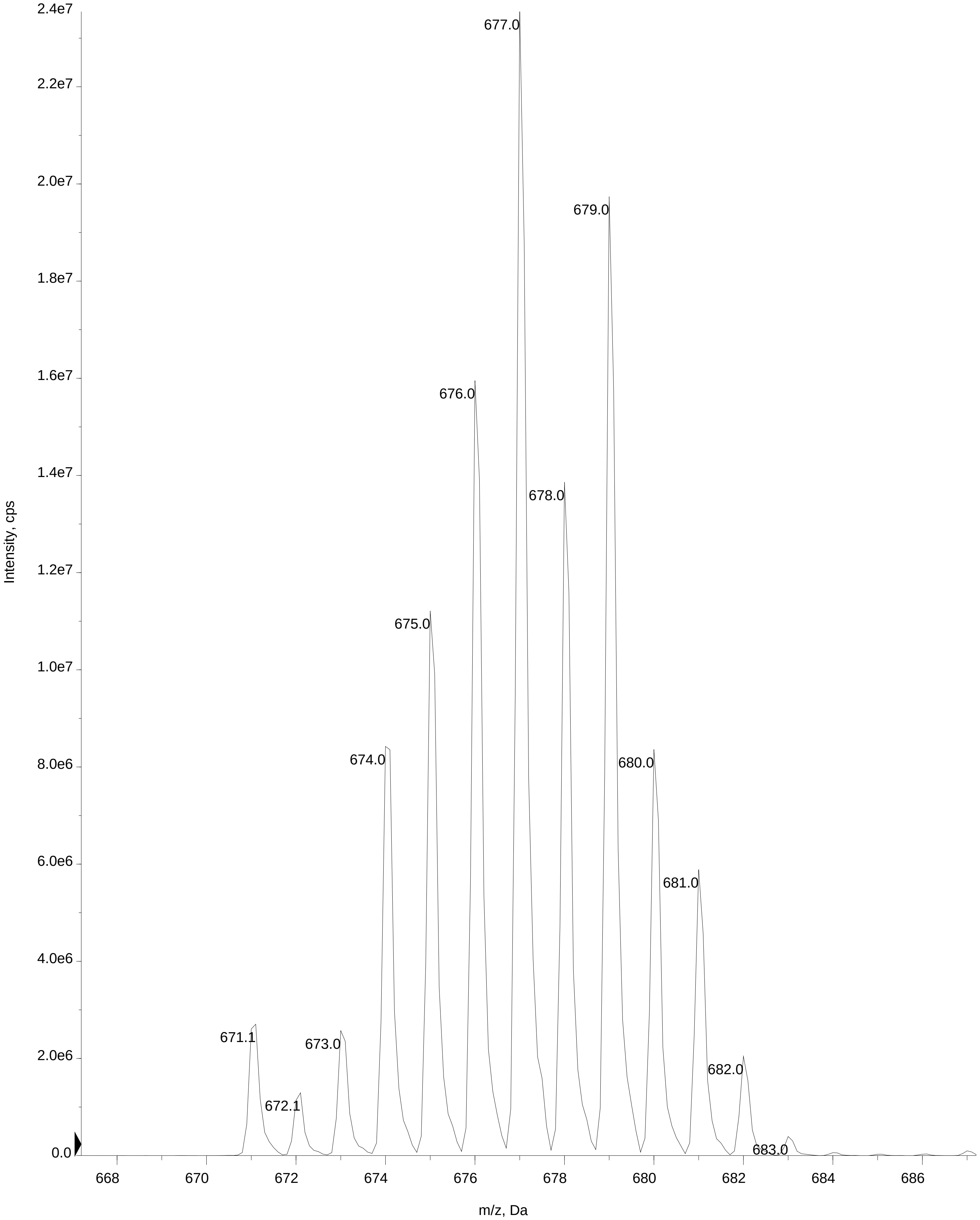

Figure S70. Experimental ESI mass spectrum of $[1{-}Cl]^+$ in methanol (positive mode).

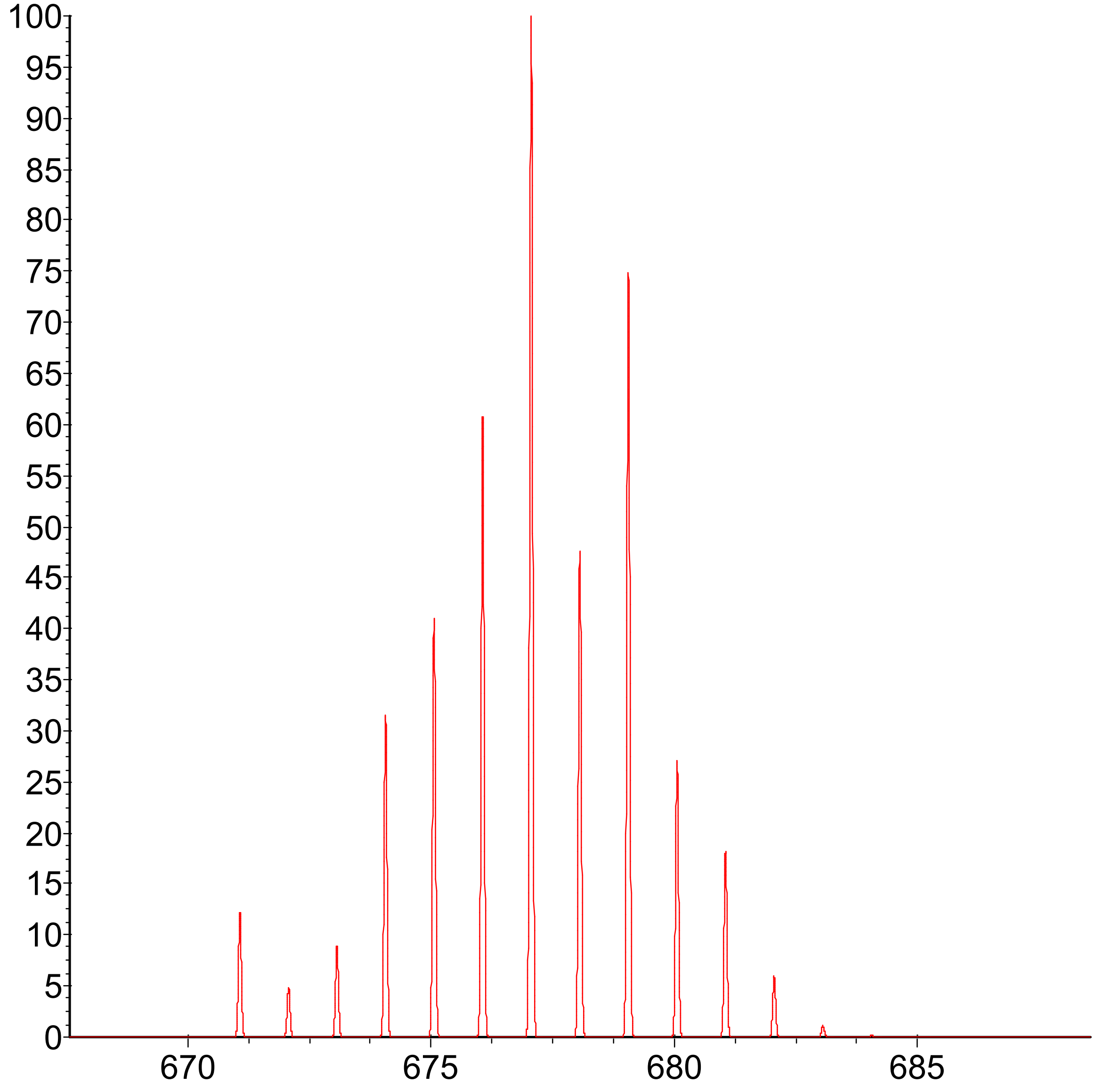

Figure S71. Simulated isotopic distribution pattern of [1–Cl]⁺.

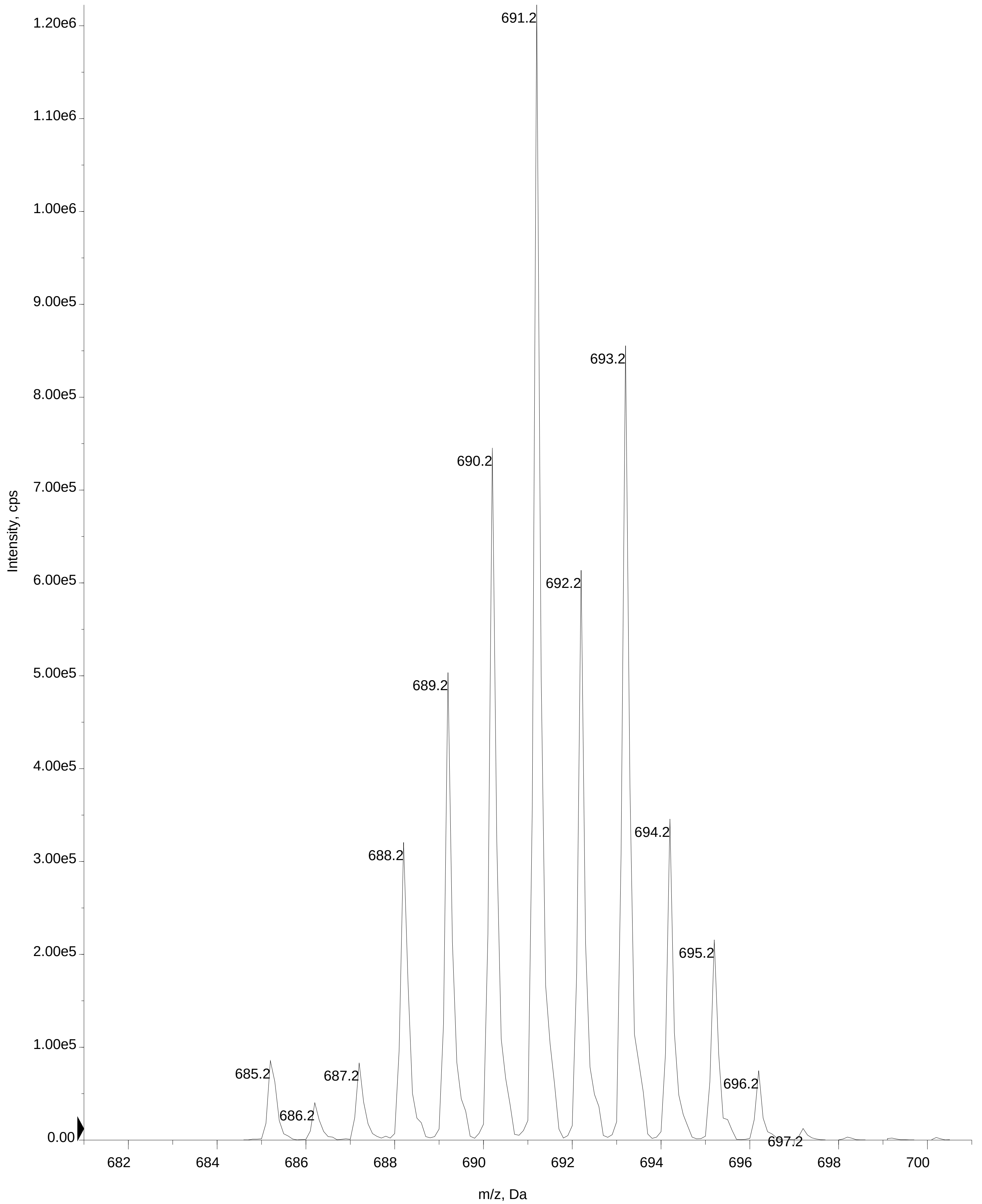

Figure S72. Experimental ESI mass spectrum of [2–Cl]$^+$ in methanol (positive mode).

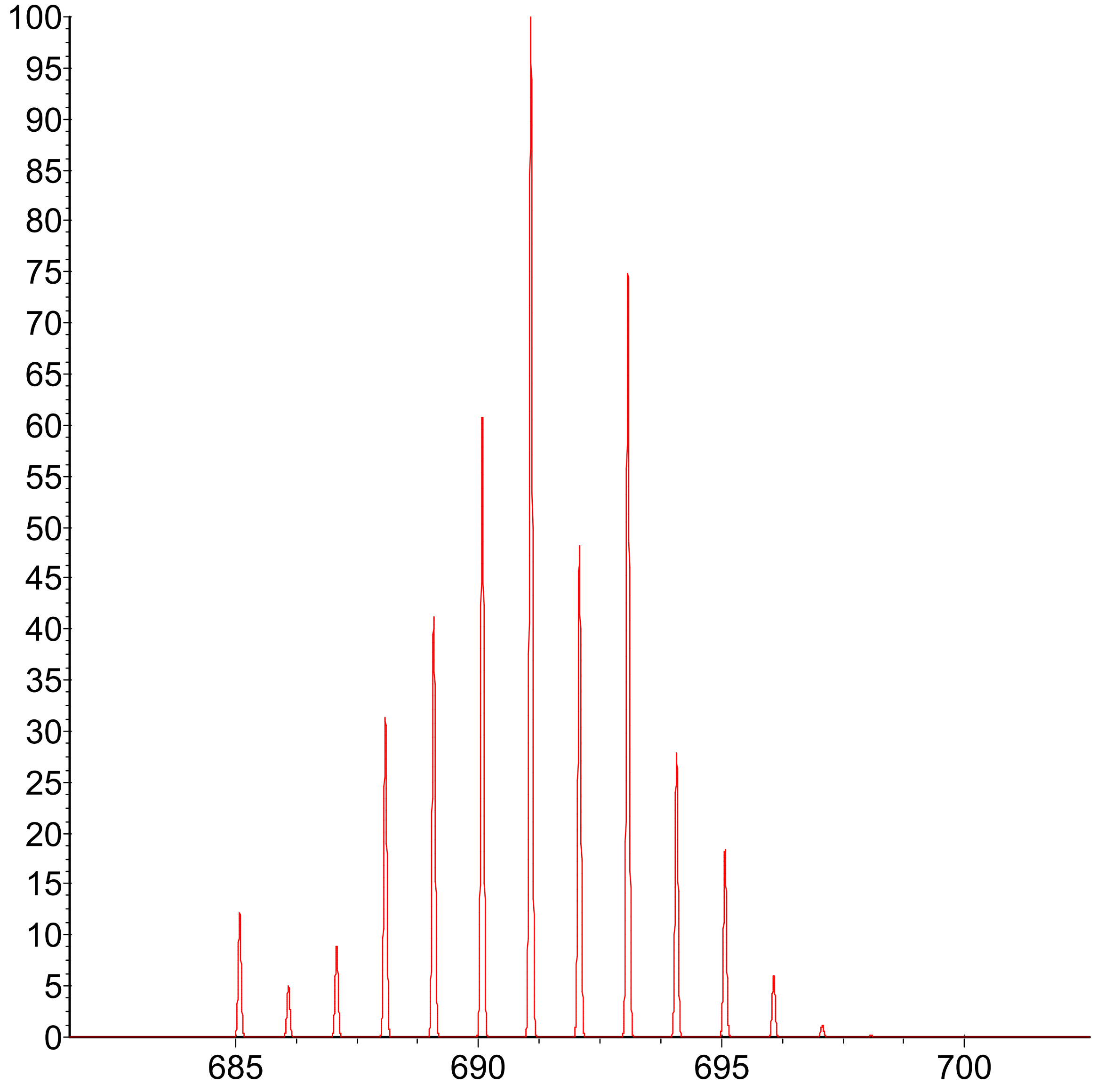

Figure S73. Simulated isotopic distribution pattern of [2–Cl]$^+$.

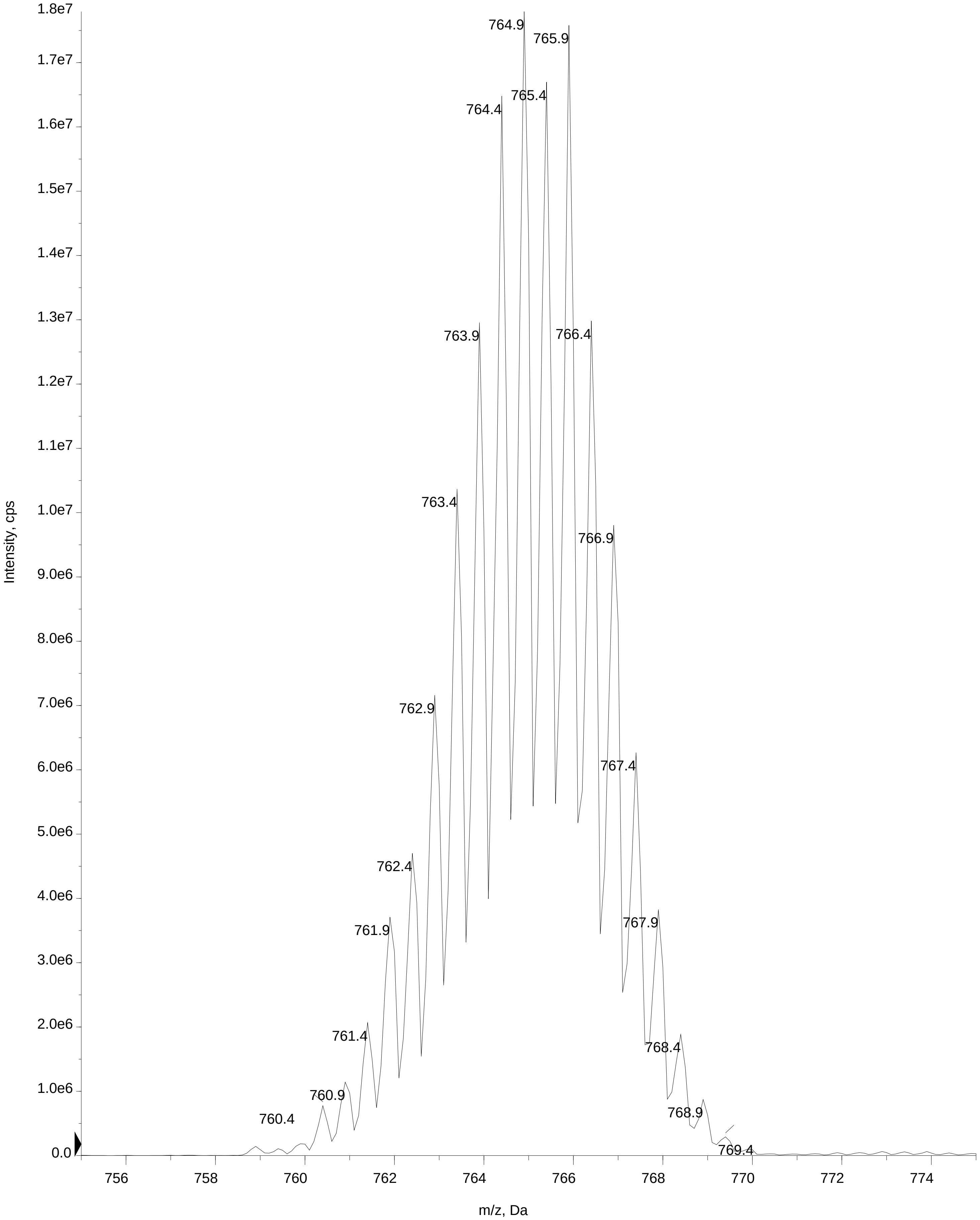

Figure S74. Experimental ESI mass spectrum of $[3]^{2+}$ in methanol (positive mode).

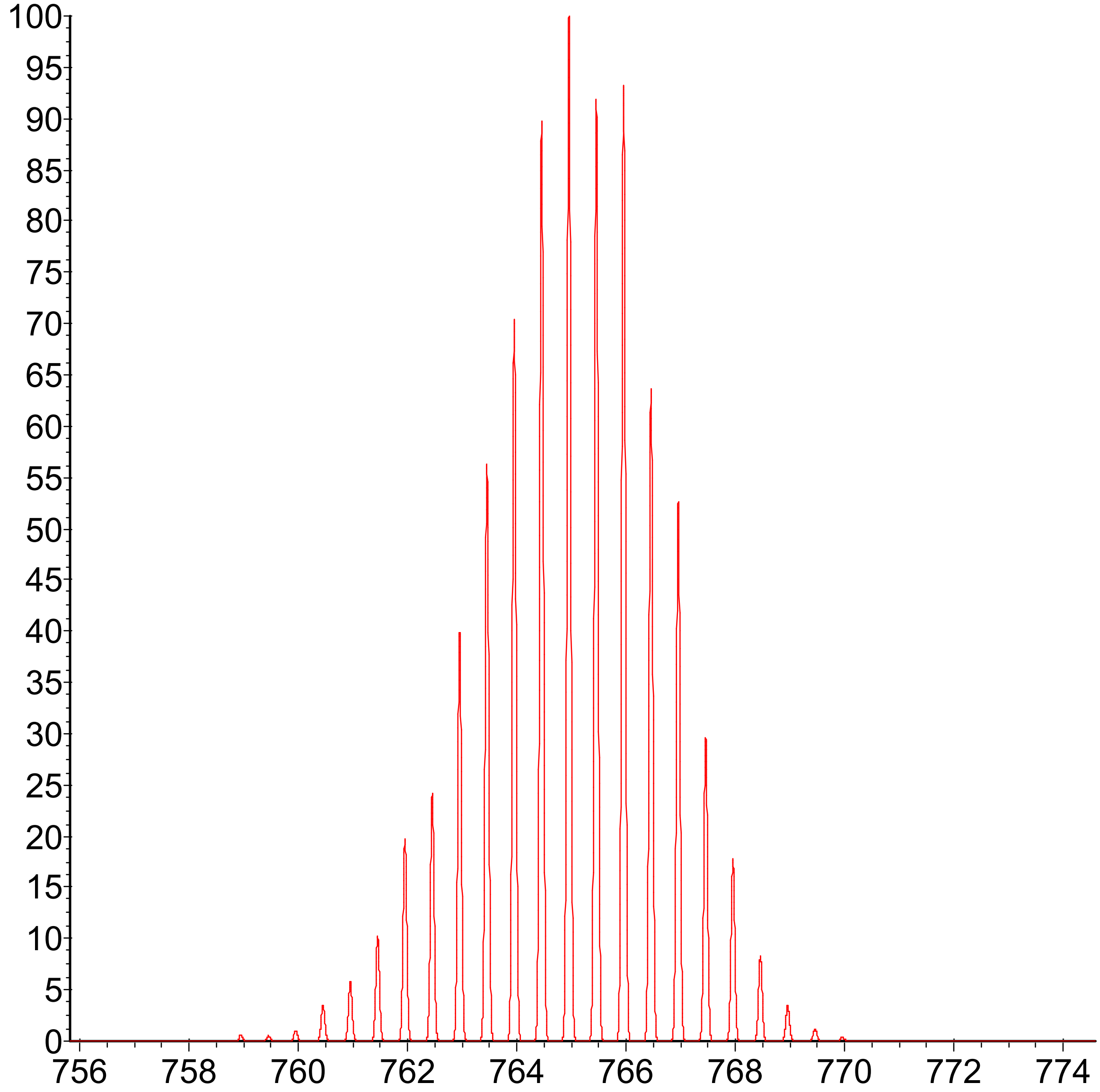

Figure S75. Simulated isotopic distribution pattern of $[3]^{2+}$.

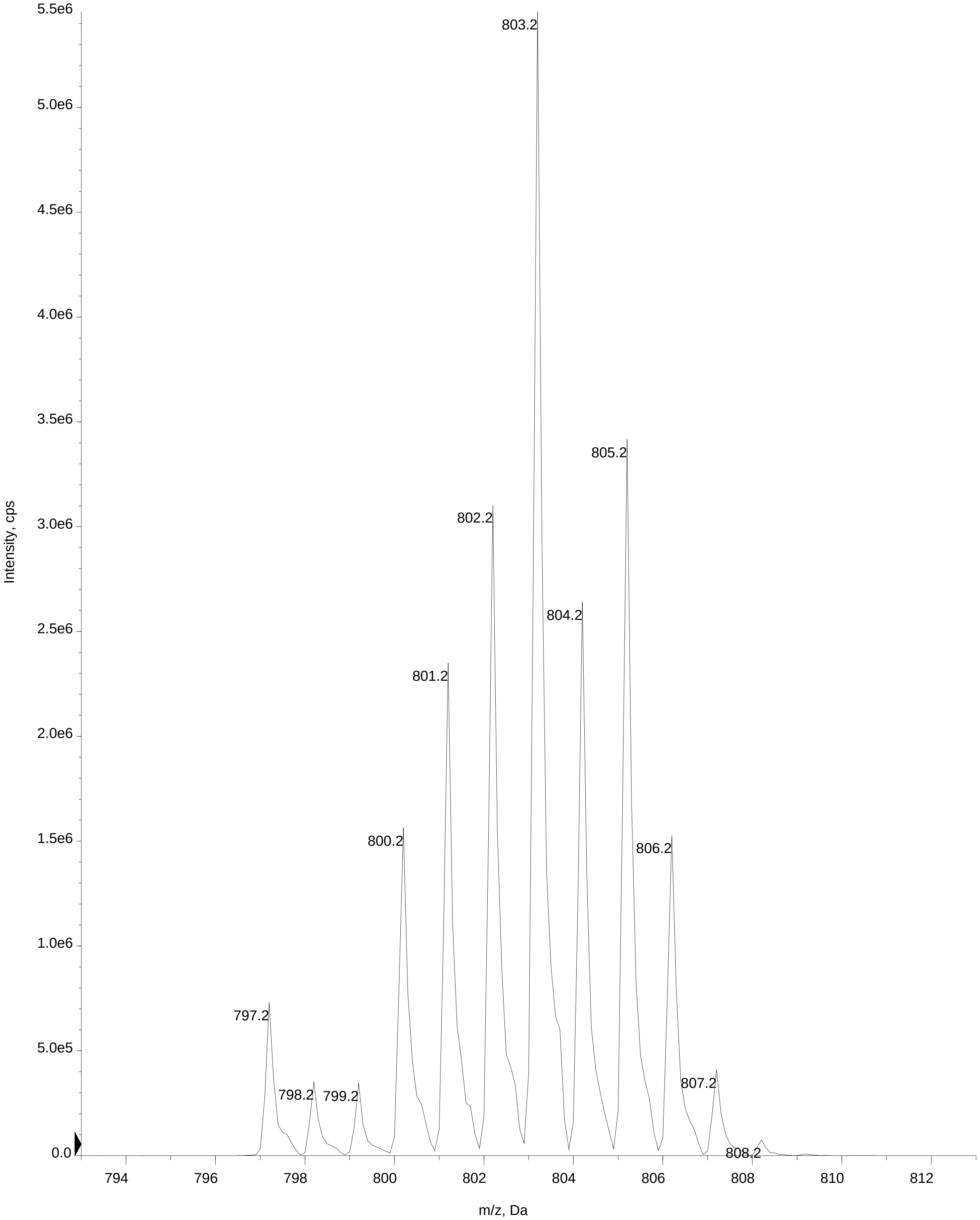

Figure S76. Experimental ESI mass spectrum of $[4]^+$ in methanol (positive mode).

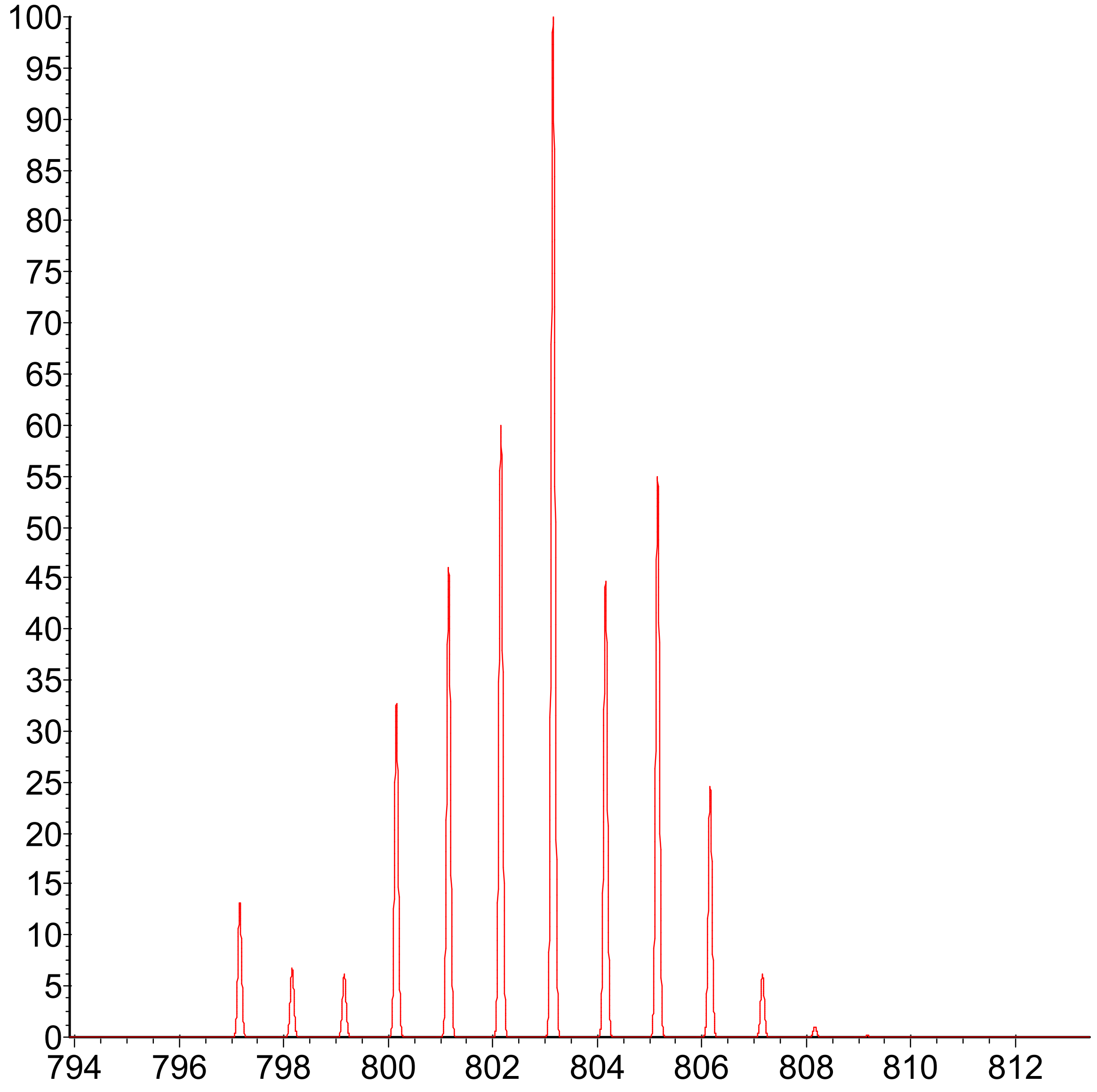

Figure S77. Simulated isotopic distribution pattern of [4]$^+$.

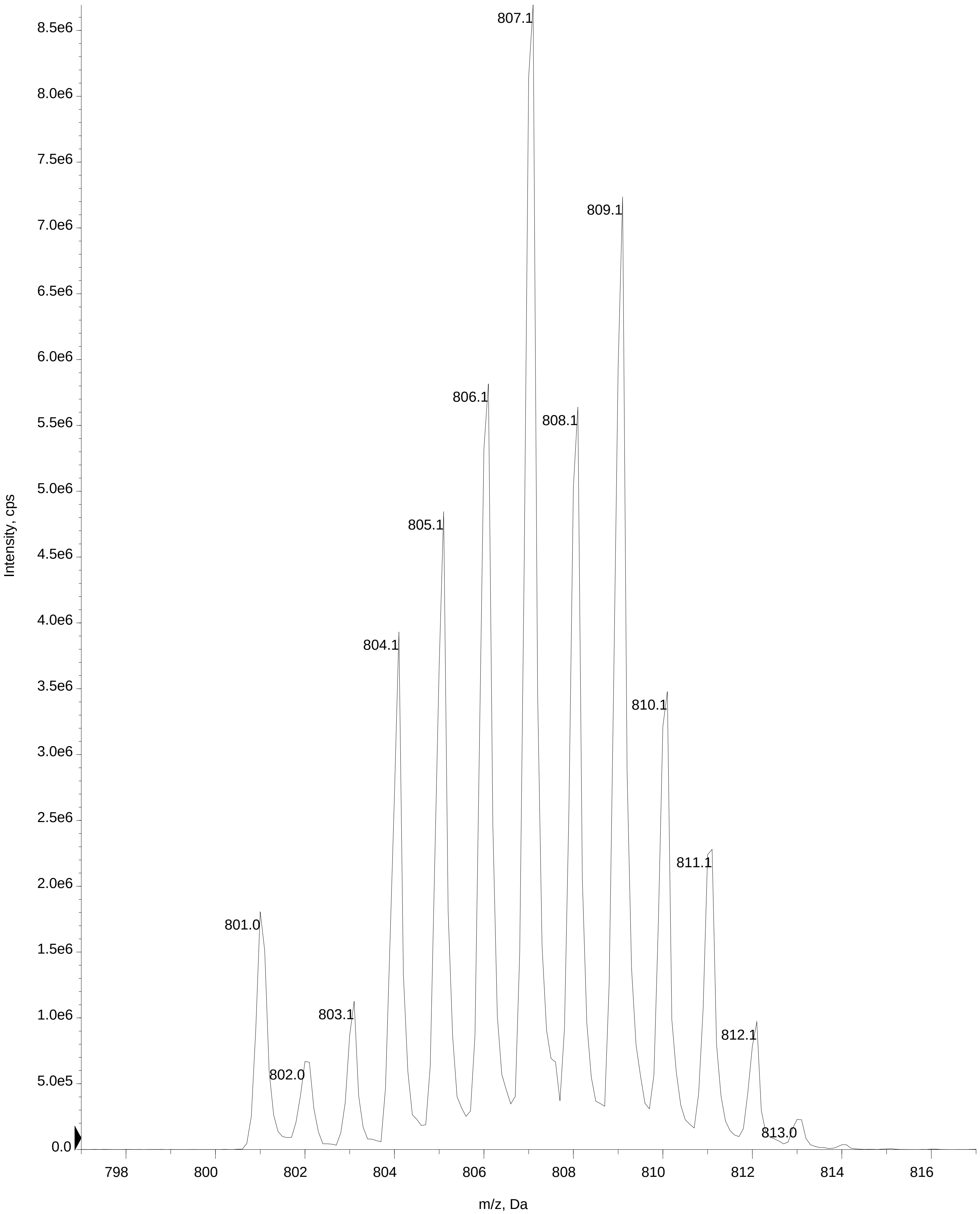

Figure S78. Experimental ESI mass spectrum of $[5]^+$ in methanol (positive mode).

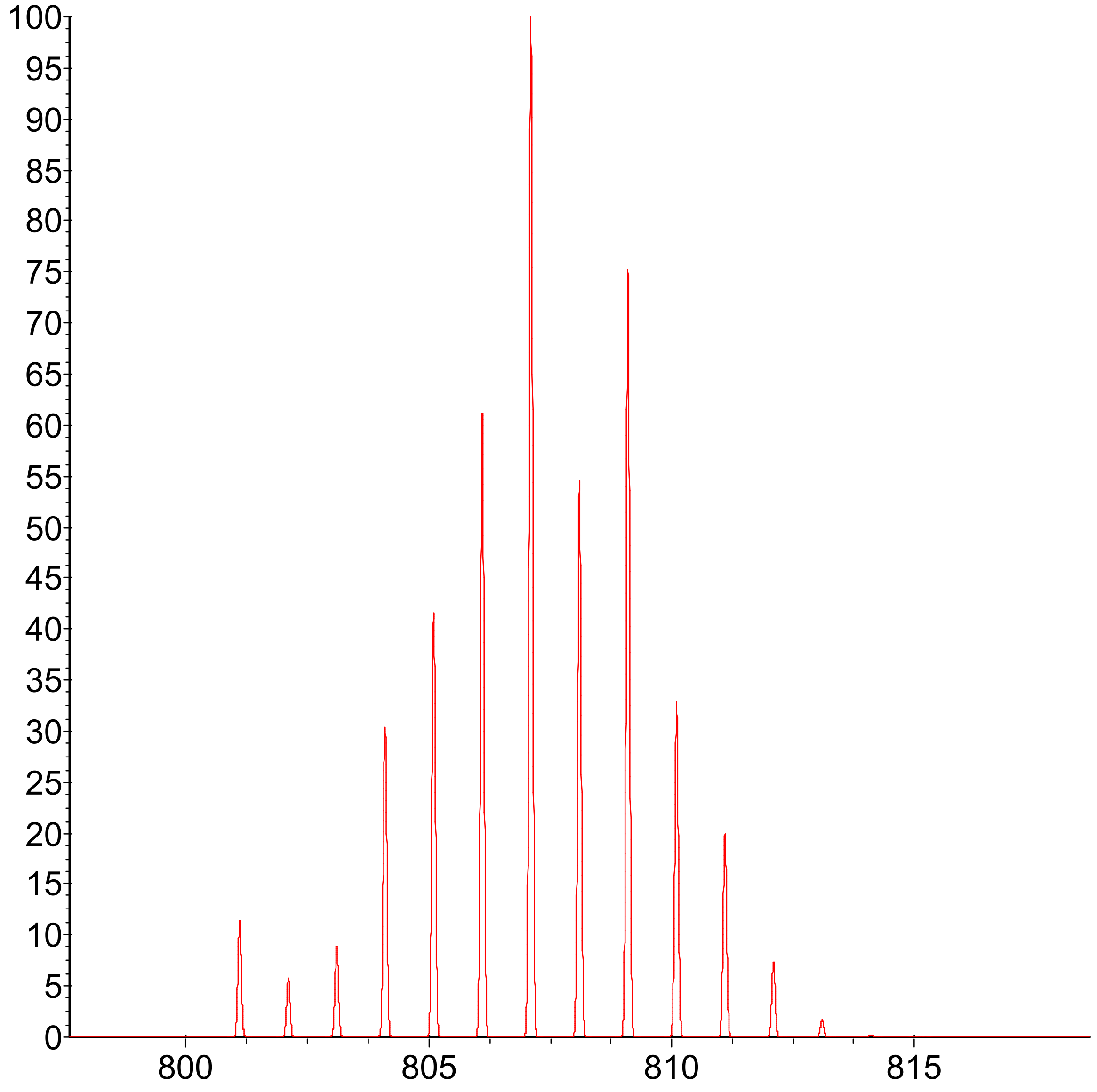

Figure S79. Simulated isotopic distribution pattern of [5]⁺.

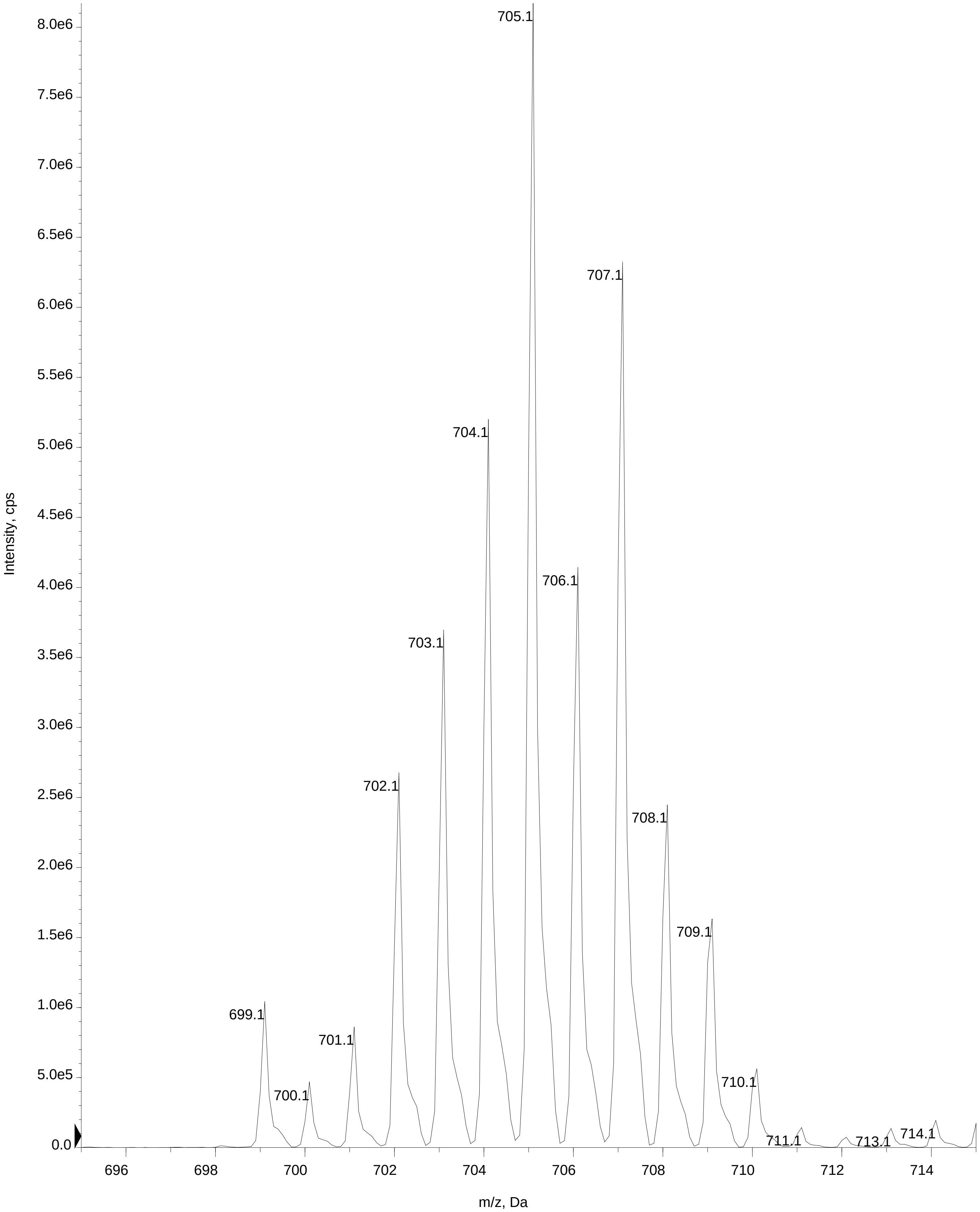

Figure S80. Experimental ESI mass spectrum of [6]+ in methanol (positive mode).

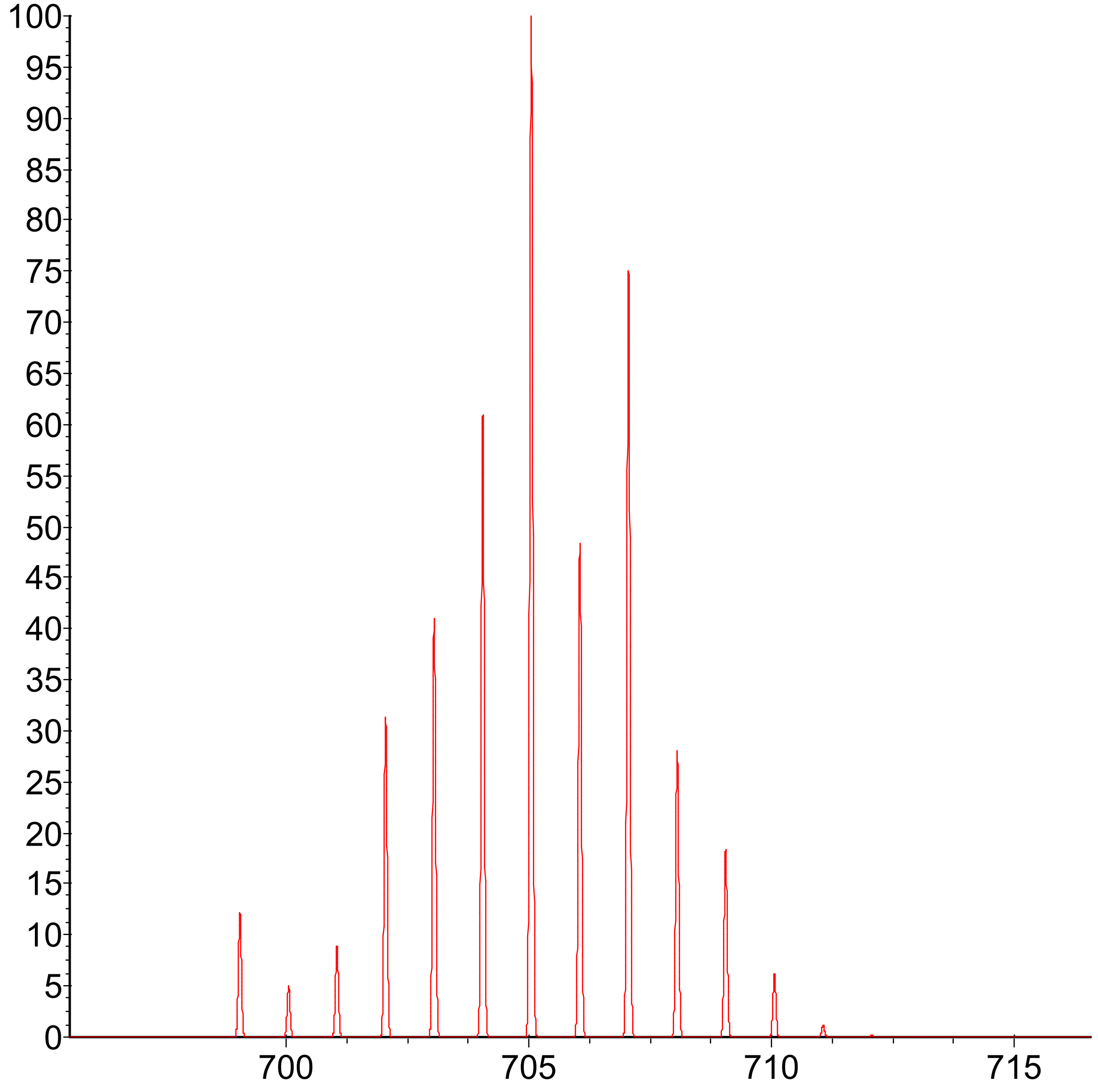

Figure S81. Simulated isotopic distribution pattern of [6]$^+$.

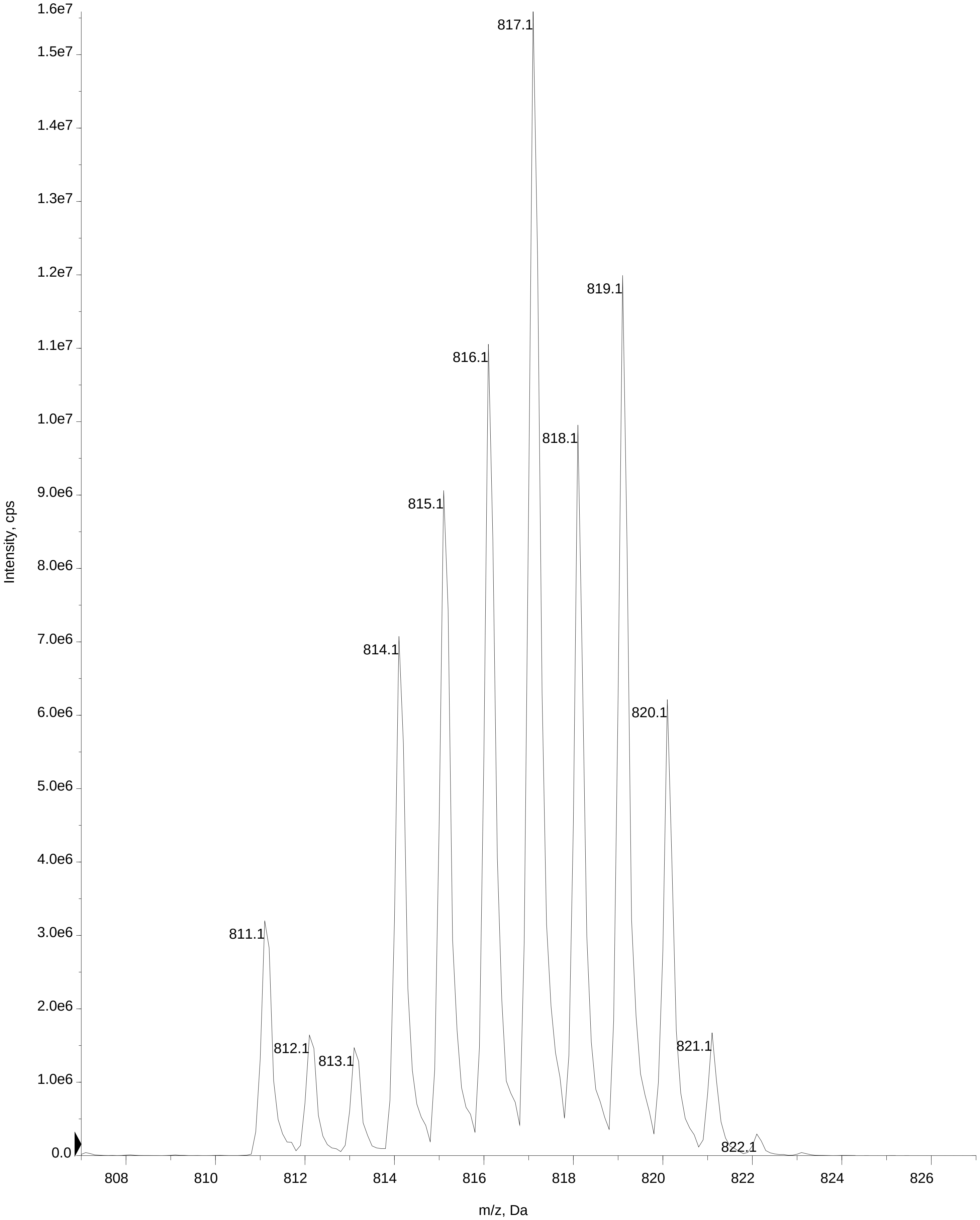

Figure S82. Experimental ESI mass spectrum of [7]$^+$ in methanol (positive mode).

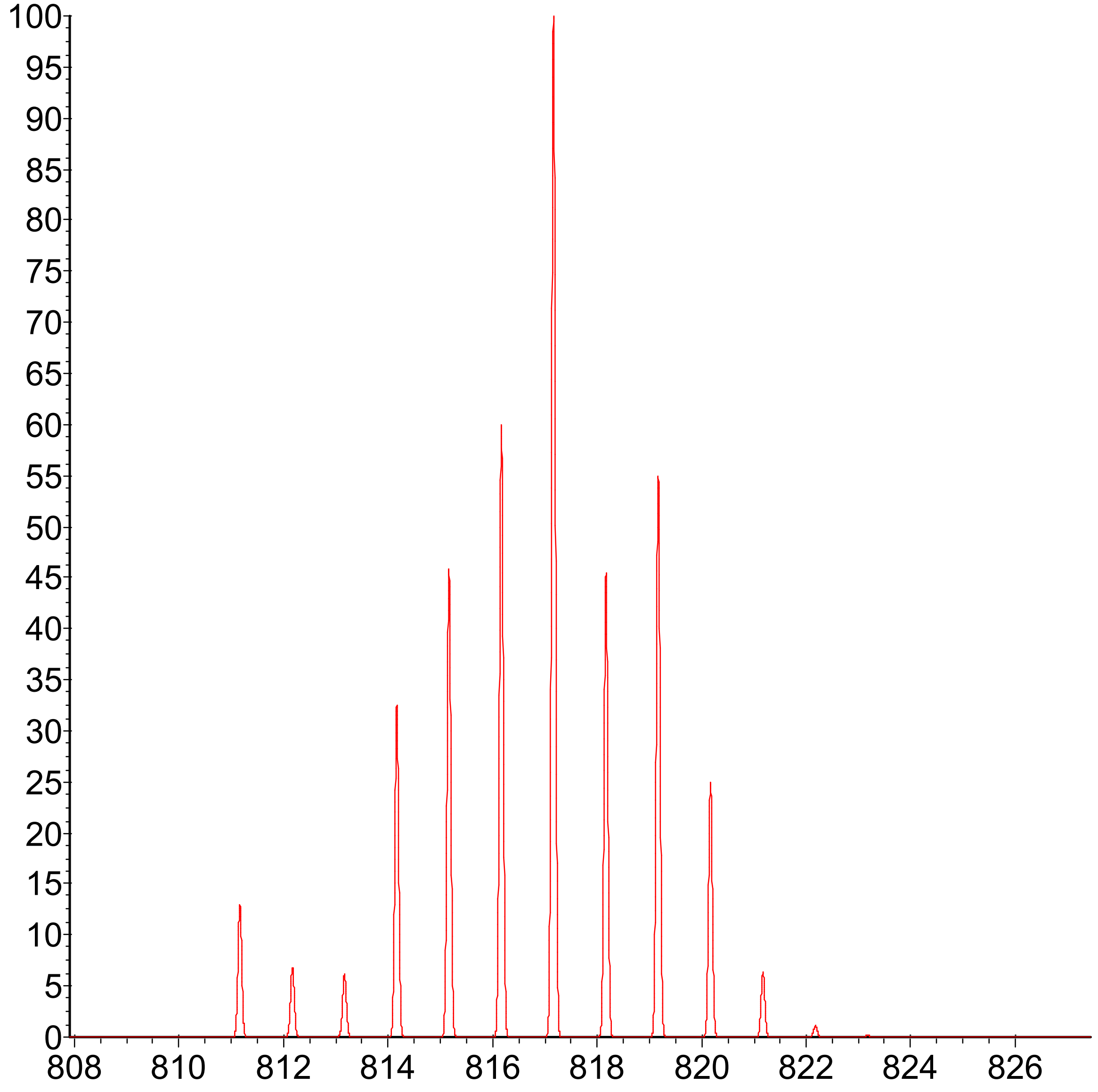

Figure S83. Simulated isotopic distribution pattern of [7]$^+$.

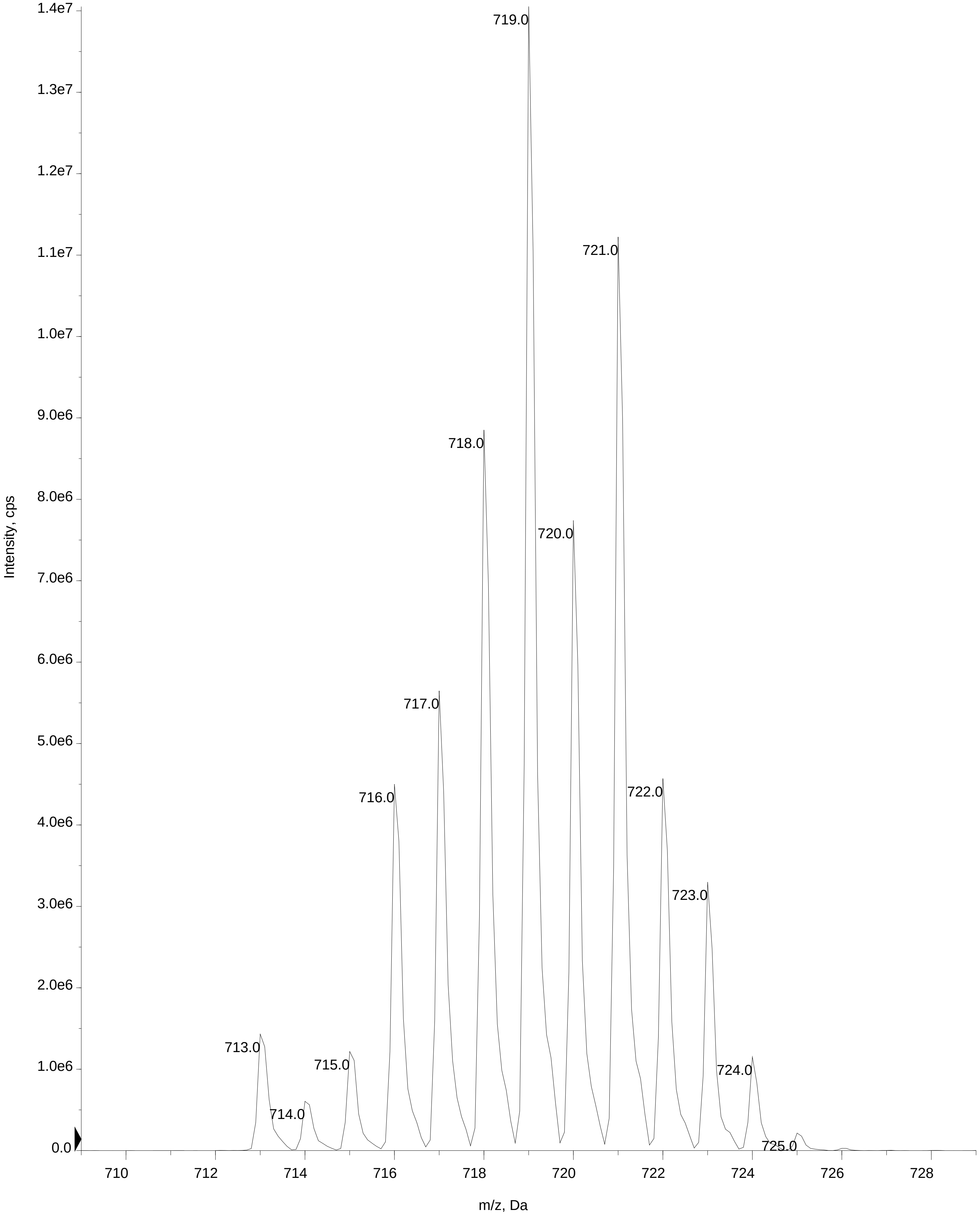

Figure S84. Experimental ESI mass spectrum of [8]+ in methanol (positive mode).

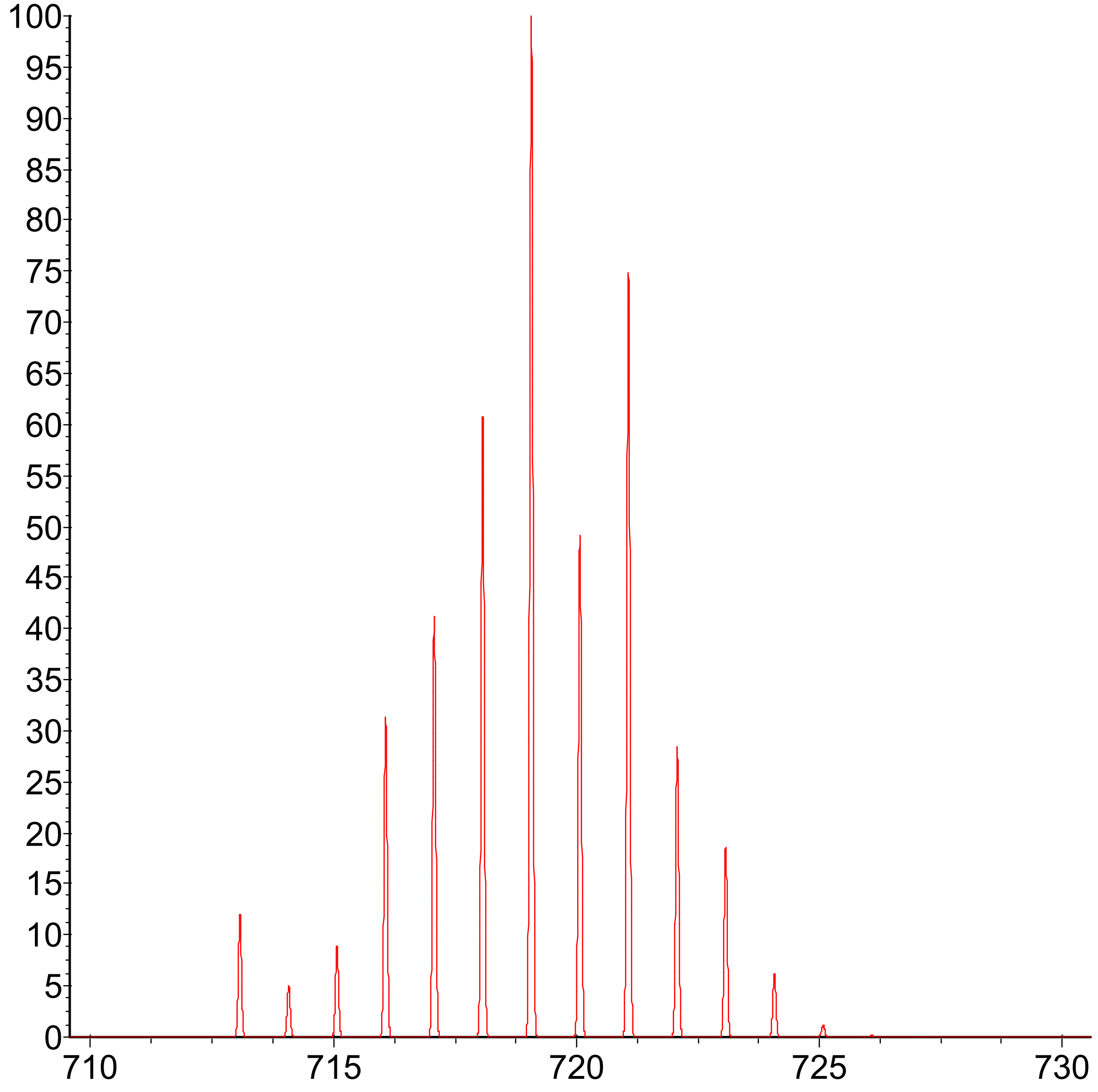

Figure S85. Simulated isotopic distribution pattern of [8]$^{+}$.
